# Delocalized quinolinium-macrocyclic peptides, an atypical chemotype for CNS penetration

**DOI:** 10.1126/sciadv.ado3501

**Published:** 2024-07-10

**Authors:** Valeria Pingitore, Jessica Pancholi, Thomas W. Hornsby, Justin Warne, Gareth Pryce, Laura J. McCormick, Julia Hill, Gauri Bhosale, Jing Peng, Lydia S. Newton, Greg J. Towers, Simon J. Coles, Ah Wing Edith Chan, Michael R. Duchen, Gyorgy Szabadkai, David Baker, David L. Selwood

**Affiliations:** ^1^ Drug Discovery, UCL Wolfson Institute for Biomedical Research, University College London, London WC1E 6BT, UK.; ^2^ Department of Biological and Health Sciences, Universidad Loyola Andalucía, Dos Hermanas, Seville 41704, Spain.; ^3^ Centre for Neuroscience and Trauma, Blizard Institute, Queen Mary University of London, London E1 2AT, UK.; ^4^ EPSRC National Crystallography Service, School of Chemistry, University of Southampton, Highfield Southampton SO17 1BJ, UK.; ^5^ Department of Cell and Developmental Biology, UCL Consortium for Mitochondrial Research, London WC1E 6BT, UK.; ^6^ Division of Infection and Immunity, University College London, London WC1E 6BT, UK.; ^7^ Department of Biomedical Sciences, University of Padua, Padua 35131 Italy.

## Abstract

Macrocyclic drugs can address an increasing range of molecular targets but enabling central nervous system (CNS) access to these drugs has been viewed as an intractable problem. We designed and synthesized a series of quinolinium-modified cyclosporine derivatives targeted to the mitochondrial cyclophilin D protein. Modification of the cation to enable greater delocalization was confirmed by x-ray crystallography of the cations. Critically, greater delocalization improved brain concentrations. Assessment of the compounds in preclinical assays and for pharmacokinetics identified a molecule JP1-138 with at least 20 times the brain levels of a non-delocalized compound or those reported for cyclosporine. Levels were maintained over 24 hours together with low hERG potential. The paradigm outlined here could have widespread utility in the treatment of CNS diseases.

## Teaser

Incorporation of a delocalized quinolinium can get macrocycles into the brain.

## INTRODUCTION

Targeting the central nervous system (CNS) with drugs remains a substantial challenge; drugs must cross the brain microvasculature into the CNS. These vessels are continuous and non-fenestrated with tight junctions limiting any transcytosis and have numerous transporters controlling the entry and exit of nutrients, hormones, and blood products. Small molecule drugs are usually designed to transmit the blood-brain barrier by diffusion across the cell membranes and this has led to several descriptions of CNS drug–like space and optimization rules or algorithms (*1*). In general, optimal CNS small molecule drug space is thought to be characterized by low molecular weight, low polarity, and high permeability, though a robust predictive model is still elusive (*2*). CNS drug–like space is regarded as smaller than the original concept of drug-likeness described by Lipinski (rule of five) (*3*) and the expanded beyond rule-of-five (bRo5) concept which considers many natural products and more modern drugs (*4*). Recent advances in drug discovery have highlighted the mechanistic advantages of larger molecules such as proteolysis targeting chimeras and macrocycles which can address an increased range of protein targets. These molecules often lie in the bRo5 range and may be outside of conventional estimations of CNS drug–like space (*4*).

Cyclosporine (cyclosporin A, CsA) is a macrocyclic undecapeptide that inhibits the mitochondrial cyclophilin D enzyme (CypD); this inhibition produces a notable neuroprotective effect. The molecular properties of cyclosporine lie in bRo5 space with a molecular weight of 1200, PSA of 279, and XLogP 7.3 (measured LogP 1.3 to 4). In addition, the molecule is a P-glycoprotein substrate and is actively transported out of the CNS. The molecule can adjust its conformation depending on its environment, such as when predominantly aqueous or in membranes (lipid-like), and belongs to a group of drugs that have been termed “chameleons” in terms of their physicochemical properties (*5*). Cyclosporine has extremely low CNS levels reported even when delivered by infusion, nevertheless extensive studies on cyclosporine have been reported in traumatic brain injury with some positive results (*6*). There is a pressing need to enable CNS penetration of cyclosporine and other macrocyclic drugs.

Mechanistically, CypD is a regulator of the mitochondrial permeability transition pore (mPTP), a final common path in multiple forms of cell death (*7*). CypD is localized to discrete brain regions including motor nerves (*8*) and γ-aminobutyric acid–releasing (GABAergic) interneurons (*9*). Knockout mouse studies in multiple sclerosis (MS) models have shown that genetic ablation of CypD provides neuroprotection (*10*). MS is a demyelinating and neurodegenerative disease of the CNS. It is a common cause of disability in young adults. Within the disease spectrum, primary progressive MS has been defined as when the disease enters a phase of “steadily increasing objectively documented neurologic dysfunction/disability without unequivocal recovery” (*11*). This is despite treatment with immune-suppressive agents. Inflammation and demyelination leading to axonal injury and neurodegeneration are evident. Demyelination also causes a redistribution of sodium channels, leading to increased sodium influx. This is followed by reversal of the Na^+^/Ca^2+^ exchanger under low ATP conditions and subsequent calcium overload. This increased Ca^2+^ concentration has several consequences but one is to induce the formation of the mPTP. This is now recognized as a key player in the degeneration of axons (*8*).

In previous studies, we identified a biochemical tool based on cyclosporine conjugated to a simple quinolinium cation as a mitochondrial CypD targeting group (JW47). The lower electron potential of mitochondria (−120 mV) compared to the cytosol (−60 mV) drives the accumulation of lipophilic cations. Despite low levels of JW47 detected in the brain, the compound demonstrated highly significant neuroprotection in an experimental model of MS (*12*). We considered that modification of the quinolinium cation to a more delocalized form could lead to a compound with improved CNS penetration.

**Fig. 1.**
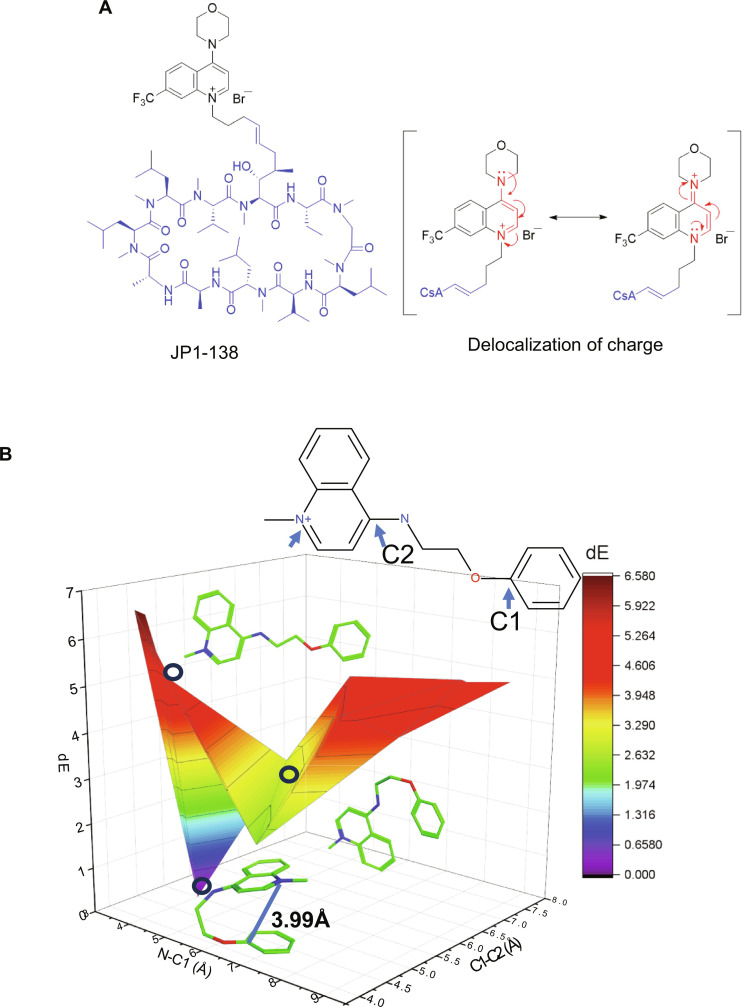
Structures, delocalization, and energy landscape of the CsA quinolinium salts. (**A**) Structure of compound, (JP1-138), the CsA scaffold is indicated (blue); the delocalization of the positive charge is shown (red). (**B**) Energy landscape of an extended quinolinium cation. *dE* is defined as the strain energy of the conformation relative to the lowest energy conformation with the same stereochemistry configuration. The N-C1 and C1-C2 distances are measured between the N and C atoms illustrated above. The lowest energy conformation with an N-C distance of 3.99 Å is shown to demonstrate the face-to-face pi-pi interaction between the two ring systems. One of the higher energy conformation is shown with an extended conformation. An edge-to-face pi-stacking conformation is also shown. *dE* units: kcal/mol.

This study reports the optimization of the quinolinium group for mitochondrial activity, preclinical safety, and improved CNS penetration. Critically, the delocalization of the cation produced improved mitochondria-protective and CNS penetration properties resulting in the identification of compound JP1-138 with 20-fold the CNS levels of cyclosporine (Fig. 1A).

## RESULTS

### Design

We reasoned that delocalization and shielding of the quinolinium cation should be investigated as a means to improve membrane permeability and potentially CNS access (*13*, *14*). Conformational searches with four-substituted quinolinium salts of varied linker chain length demonstrated that a three-atom link to an aromatic group led to an energy-minimized structure with the aromatic ring positioned over the cation (Fig. 1B). Fractional property calculations were also conducted to estimate the contribution to the overall physicochemical properties of the molecules. In this regard, the quinolinium and short linker moieties made relatively small changes, this contrasted with the canonical triphenylphosphonium mitochondrial targeting group (*15*) which has an overall +6.7 contribution to logP and higher molecular weight addition (table S1). Compounds where the cations were shielded with potential pi cation interactions such as JP1-068 gave an increase in molecular weight and logP, whereas those with delocalized cations such as JW76 gave more modest changes to physicochemical properties. We determined to synthesize molecules of both types for biological evaluation.

### Cation delocalization

**Fig. 2.**
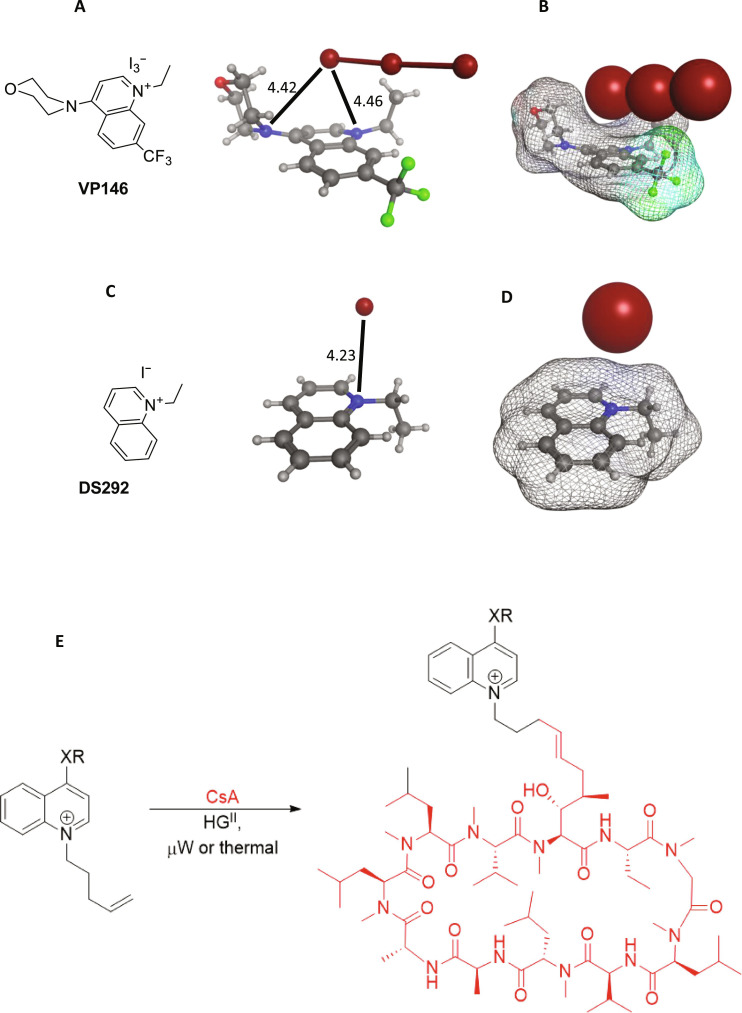
X-ray structures of model cations and general synthesis of the analogs. (**A**) Ball and stick model of the 4-morpholino quinolinium salt showing distances to the N atoms. (**B**) Connolly surface generated for the quinolinium with triiodide anion (space-filling). (**C** and **D**) Similar models generated for unsubstituted quinolinium. Figures were generated using Molecular Operating Environment (MOE). (**E**) A synthesis of quinolinium cations by olefin cross-metathesis. Appropriately substituted quinolinium salts were linked to CsA by cross-metathesis using the Hoveyda-Grubbs second-generation catalyst.

Our design assumed a classical delocalizing effect across the quinolinium ring as depicted (Fig. 1A), but we had no direct evidence for this. Accordingly, we synthesized the simple ethylated quinolinium salts (with or without the 4-morpholino) and obtained their x-ray structures (VP146 and DS292; Fig. 2) (*16*, *17*). We observed that VP146 crystallized with a triiodide counter ion and that the two nitrogens were almost equidistant from the terminal iodine (Fig. 2, A and B). In triiodinated compounds, the bond lengths of I_3_^−^ vary depending on the nature of the counter cation as the anion is easily polarized, and usually, one I─ I bond is shorter than the other. Only in the presence of a large counter cation (e.g., quaternary ammonium) does the triiodide present highly symmetrical bonds (*18*, *19*). In the case of VP146, the presence of a delocalized positive charge on both nitrogen atoms of the conjugated system was further confirmed by the high symmetry observed in the I_3_^−^ bond length values obtained by x-ray diffraction (2.91 and 2.93). In contrast, DS292 crystallized as a monoiodide rather than a triiodide, which may be due to the absence of a positive charge delocalization effect. The anion was not located above the quinolinium ring (Fig. 2, C and D). Together, the results indicate classical delocalization of the positive charge.

### Synthesis

The general synthetic route for quinolinium derivatives of CsA involved late-stage functionalization using olefin cross-metathesis (Fig. 2E). The intermediate quinolinium salts were prepared by alkylation with various electrophiles depending on the exact structure. The synthesis of 4-oxa–substituted quinolines was achieved as outlined (see Supplementary Materials and Methods and fig. S3). O-alkylated quinolines were synthesized from 4-quinolinol by treatment with the corresponding alkyl bromide, silver(I) oxide, and tetrabutyl ammonium iodide in dichloromethane (*20*). The 4-methoxyquinoline was synthesized by nucleophilic aromatic substitution of 4-bromoquinoline with sodium methoxide. The treatment of both products with 5-bromopent-1-ene in acetonitrile (MeCN) allowed the alkylation of the endocyclic nitrogen obtaining the final desired quinolinium salts (fig. S3 and table S2). Two *O*-linked quinolinium salts with the alkene linker on the oxygen atom were synthesized by refluxing 4-(pent-4-en-1-yloxy)quinoline with either (2-bromoethyl)benzene or 1-bromo-3-phenylpropane in MeCN (fig. S4 and table S2). Last, quinolinium derivatives 4-*N*–substituted were synthesized by substitution of 4-chloroquinolines with the corresponding secondary amine followed by quaternarization of the endocyclic nitrogen with 5-bromopent-1-ene (fig. S5 and table S3).

All the quinolinium cations were conjugated to cyclosporine by olefin cross-metathesis using the Hoveyda-Grubbs second-generation catalyst as outlined (Fig. 2E) (for the synthetic details see Supplementary Information). For larger scale preparations an inhibitor of catalyst breakdown 2,6-dichloroquinone was added (*21*, *22*, *23*). As expected the NMR spectra of the products were highly complex but most of the quinolinium structural features could be clearly observed (Supplementary information). Cyclosporine is known to adopt different conformations by NMR: For our molecules, we observed either broad signals (JP1-138) with a 4-morpholino quinolinium or sharp signals (JW76) 4-*N*,*N*′-dimethylaminoquinolinium which likely correlated with the flexibility of the additional functionality.

### Biological evaluation

#### Cyclophilin binding

**Fig. 3.**
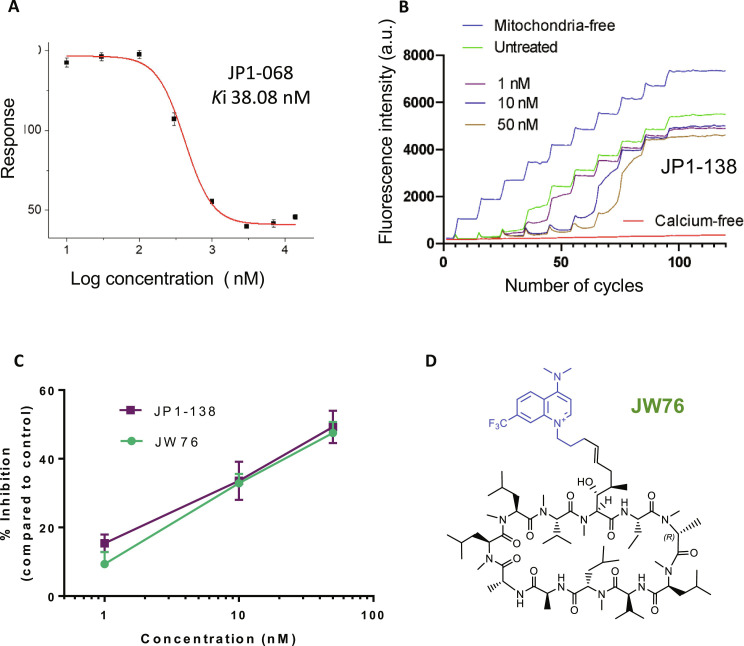
Evaluation of CypD protein binding and mitochondrial Calcium Retention Capacity for selected analogs together with the full chemical structure of JW76. (**A**) CypD binding of JP1-068. Determined using a fluorescence polarization assay with a biotinylated-CsA probe. (**B**) Evaluation of the effect of JP1-138 on mitochondrial Ca^2+^ retention capacity (CRC). Representative traces of the CRC assay in isolated rat liver mitochondria. Fluo-5 N fluorescence was measured in the extramitochondrial solution following repeated additions of Ca^2+^ (10 μM; for details, see Materials and Methods). An increase in fluorescence indicates loss of Ca^2+^ retention due to permeability transition (PT) pore opening. (**C**) Dose response of JP1-138 compared to JW76 on PT inhibition [expressed as a % increase in CRC compared to dimethyl sulfoxide (DMSO) treatment]. (**D**) Full structure of JW76.

This was determined using a fluorescence polarization assay (*12*). The binding sites for cyclosporine are tightly conserved (*24*). Cyclophilin A (CypA) binding was used as a convenient model (the recombinant protein being more stable), and CypD binding was evaluated for selected analogs. Evaluation of the 4-oxa–substituted analogs (table S2) revealed that only the 4-methoxy compound TWH32 retained potent affinity but still lost fourfold binding over cyclosporine. The inverted substitution with the attachment of the quinolinium via the 4-oxa position (table S2) showed similarly poor activity showing approximately 20-fold weaker activity than CsA. In contrast, a series of 4-aza–substituted compounds demonstrated potent cyclophilin binding with some showing superior binding affinity or equivalent affinity to cyclosporine (table S3). Notably, JP1-068 demonstrated consistent activity and was evaluated for CypD alongside CypA. The compound showed good activity for both proteins with *K*_i_ (inhibition constant) values of 38.08 nM for CypD compared to 10 nM for CypA (Fig. 3A and fig. S1). In this assay, cyclosporine also gives a small CypA selectivity (*K*_i_ 22.6 nM for CypA versus 73 nM for CypD). The simplest analog JW76 with a NMe_2_ substituent and 7-CF_3_ substituent showed a twofold loss of activity over cyclosporine for CypA binding (table S3).

#### Permeability transition pore blocking effect in mitochondria

A calcium retention capacity (CRC) assay assessment of the mPTP activity was used (*25*). In this assay, isolated mitochondria from rat or mouse liver or hippocampal neurons are prepared. The graphics show measurements of extramitochondrial calcium reported by a low-affinity calcium indicator (calcium green) made in a plate reader in response to sequential additions of aliquots of calcium (10 μl of 100 μM CaCl_2_) to preparations of isolated mitochondria (Fig. 3B). mPTP opening is indicated by the failure of the mitochondria to take up and buffer the calcium additions. Data are expressed as the percentage inhibition of pore opening. We evaluated our molecules for activity at 500 nM; there was no obvious correlation between potency (*K*_i_) and the level of inhibition presumably because CRC is a complex property requiring not just CypD inhibition but also penetration to the inner mitochondrial membrane location. That said, only the more potent analogs give improved CRC inhibition (fig. S2 and table S2). Notably, the 4-amino–substituted compounds JP1-068 and JW76 maintained their ability to protect mitochondria from increasing calcium concentrations at concentrations as low as 10 nM.

To test the potential of these early molecules for brain penetration, we conducted a simple pharmacokinetic study in mice measuring plasma and brain levels by liquid chromatography–tandem mass spectrometry and compared these to our earlier prototype compound JW47. Both JW76 and JP1-068 showed high plasma levels persisting at 4 hours (50,800 and 6410 ng/ml, respectively) and both showed much higher brain levels (668 and 110 ng/ml, respectively, at 1 hour) than the biochemical tool JW47 (18 ml at 1 hour) indicating that delocalization of the cation was favorable for brain penetration (table S5). hERG activity of JP1-068 was evaluated and showed an IC_50_ (median inhibitory concentration) of 1 to 2 μM which compared unfavorably to cyclosporine at 3 to 4 μM (table S6). Further analysis showed that molecules of the JP1-068 type were consistent with a known hERG pharmacophore (*26*) in the 4-alkylaryl substituent of the quinolinium, and despite being presented in the context of the much larger cyclosporine unit, this was still able to produce an hERG effect. In contrast, the *N*,*N*′-dimethyl analog JW76 showed little hERG activity (table S6) and was selected as a lead molecule for a focused structure activity set.

**Fig. 4.**
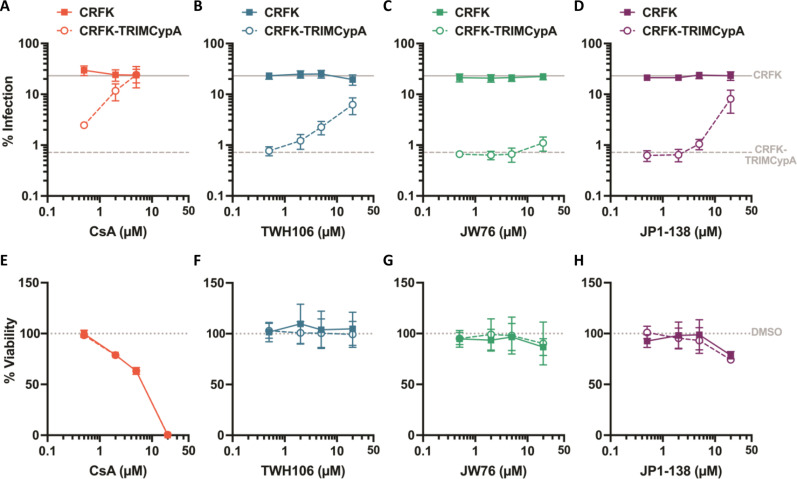
Cellular CypA selectivity of JW76 and JP1-138 compared to known CypA inhibitors CsA and TWH106. (**A** to **D**) Crandell-Rees Feline Kidney (CRFK) cells transduced with either empty vector (filled squares) or TRIM-CypA (open circles) were infected with a green fluorescent protein (GFP)–encoding HIV-1 vector (MOI 0.2) in the presence of DMSO or serial dilutions of compounds: CsA (A), TWH106 (B), JW76 (C), and JP1-138 (D). Viral infection (% GFP-positive cells) was measured by flow cytometry 48 hours after infection (means ± SD of two independent experiments each performed in duplicate). (**E** to **H**) Viability of CRFK and CRFK-TRIMCypA cells following treatment with compounds for 48 hours measured by MTT assay and presented as % DMSO (means ± SD of two independent experiments each performed in duplicate).

In the focused set we maintained the 4-aza quinolinium but cyclized the *N*,*N*′-dimethylamino group as piperidine, pyrrolidine, or morpholine to give a range of polarities and electron donating ability. We also varied the overall electron density of the quinolinium system by inclusion of the trifluoromethyl group in the benzo ring (table S7). The biological evaluation demonstrated that potent cyclophilin binding was maintained throughout this focused set, and CypD evaluation showed better activity than for the diverse analogs. The two pyrrolidine analogs showed less cyclophilin binding than the others and were not evaluated further. Detailed profiling of the remaining compounds revealed that all were highly potent in the CRC mitochondria assay at 10 nM (table S7), and JP1-138 was approximately more equipotent than JW76 in CRC (Fig. 3, C and D). To evaluate the cellular selectivity over CypA, we used an HIV-1–based cellular assay responsive to CypA inhibition as previously described (*12*). HIV-1 infection of cell lines can be inhibited by the expression of an artificial antiviral protein, comprising the RBCC domains of owl monkey tripartite motif-containing protein 5 (TRIM5) fused to human CypA (TRIM-CypA). TRIM-CypA inhibited viral infection by 32-fold in the absence of a compound (Fig. 4, A to D, gray lines). Both CsA and TWH106 [a CypA inhibitor structurally distinct to CsA (*27*)] rescued infectivity of restricting cells through CypA inhibition (Fig. 4, A and B, open circles). JW76 rescued infectivity poorly and only at 20 μM (Fig. 4C), suggesting minimal CypA binding in cells. JP1-138 showed CypA inhibition evidenced by the rescue of infectivity at 20 μM and very minimally at 5 μM (Fig. 4D). CsA was highly toxic to both non-restricting and restricting cells at 20 μM (Fig. 4E), and therefore, the infection could not be measured at this concentration. TWH106 and JW76 were not toxic at any concentration tested (Fig. 4, F and G), while JP1-138 showed some toxicity at 20 μM (Fig. 4H).

**Fig. 5.**
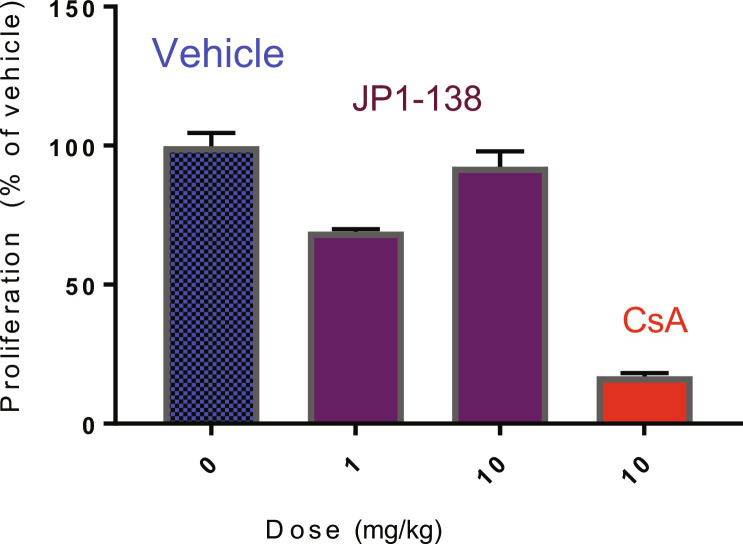
Immune suppression data on JP1-138. Animals (*n* = 3) received daily CsA or JP1-138 in dimethyl sulphoxide (days 0 to 3) before the epicutaneous application of 2.5% oxazolone in acetone olive oil (4:1) onto the dorsum of the ear. The draining auricular lymph nodes were removed and pooled (*n* = 3 to 4 animals per group) on day 3 and cultured overnight. Proliferation from 5 × 10^5^ lymph node cells was assessed using CellTitre 96 nonradioactive cell proliferation assay and represents means ± standard error of replicate cultures.

Given this encouraging dataset, we next evaluated the compounds in a range of conventional lead-like in vitro assays (Table 1). The compounds displayed highly variable binding to plasma proteins and evidence of tight binding to brain tissue binding. Pleasingly hERG inhibition was weak with only modest inhibitions at a supraphysiological concentration of 25 μM except for JP1-141 which displayed 65% inhibition at 25 μM compared to 25% for cyclosporine. Metabolism in human and mouse hepatocytes was generally low, though JP1-164 showed higher relative clearance in human hepatocytes (Table 1).

**Table 1.** Key data for lead optimization. SD, standard deviation of the mean; SE, standard error of the mean; fu, fraction unbound; ND, not determined.

Compound	Plasma protein (fu)	SE	Brain tissue (fu)	hERG (% inhibition at 25 μM)	SD	Hepatocytes (human) clearance	SE	Hepatocytes (mouse) clearance	SE
CsA	0.29 ±	0.19	ND	19.8 ±	4.84	6.73 ±	4.84	20 ±	3.68
JW76	0.0039 ±	0.00019	ND	16.3 ±	1.68	5.62 ±	1.68	6.81 ±	1.6
JP1-180	0.17 ±	0.0023	ND	9.75 ±	1.14	4.65 ±	1.14	4.07 ±	1.56
JP1-138	*		0.00084	37.7 ±	1.65	7.62 ±	1.65	9.61 ±	1.94
JP1-141	*		0.01	63.9 ±	0.59	3.62 ±	0.59	3.59 ±	1.25
JP1-166	0.43 ±	0.23	ND	31.4 ±	1.61	8.01 ±	1.61	9.47 ±	1.52
JP1-164	0.69†		0.01	14.2 ±	0.85	22.4 ±	0.85	5.89 ±	2.29

*Variable results.

†Single assay only.

The remaining five compounds were evaluated for plasma and brain levels in a pharmacokinetic experiment in mice. Results are summarized in Table 2. The compounds showed long plasma half-lives and high area under the curves (AUCs) with low clearance. JP1-166 had relatively low plasma and brain levels, while JP1-141 exhibited some toxicity at the 10 mg/kg dose and these compounds were dropped from further evaluation as was JP1-164. Brain levels at hundreds of nanomolar are at least 20-fold greater than our prototype compound JW47 (Table 2) or cyclosporine (*28*). The compound JP1-138 was selected for further studies in comparison with JW76.

**Table 2.** Pharmacokinetic parameters of the focused set in mice.

Compound	Half-life	AUC 0-t	AUC 0-inf_obs	Cl_obs	Brain concentrations at 24 hours*	
	hour	ng/ml.hour	ng/ml.hour	(mg/kg)/(ng/ml).hour	nM	ng/ml
JP1-180	5.06	59,320	62,120.99	1.61 × 10^−04^	142	196
JP1-138	5.81	215,812.5	225,643.3	4.43 × 10^−05^	391	592
JP1-141	4.04	102,733.3	104,527.9	9.57 × 10^−05^	642	927
JP1-166	6.75	54,976.67	59,949.68	1.67 × 10^−04^	86	124
JP1-164	5.68	133,902.5	140,980.1	7.09 × 10^−05^	380	574

*Dosed at 10 mg/kg, ip. AUC, area under the curve; Cl, clearance.

A longer-term pharmacokinetic study demonstrated that JP1-138 has higher AUC than JW76 in plasma (198,000 ng/ml.hour compared to 67,000 ng/ml.hour. In the brain, JP1-138 is still detectable at 48 hours with about 2% of the plasma AUC observed (table S8). JW76 was not detectable in the brain at 48 hours.

Immune suppression was assessed in vivo using the oxazolone-induced contact sensitivity immunosuppression assay. JP1-138 was compared to cyclosporine at 1 and 10 mg/kg (Fig. 5D). While the 10 mg/kg dose elicited a slight immune suppressive effect, the 1 mg/kg dose was not significantly different from the control; in contrast, cyclosporine at 10 mg/kg potently suppressed contact sensitivity.

## DISCUSSION

The applications for CypD inhibitors are not limited to MS (*29*), other CNS indications include Parkinson’s disease where genetic ablation of CypD delays disease onset and extends the life span of mutated α-synuclein Parkinson’s model mice (*30*). In amyotrophic lateral sclerosis, CypD knockout similarly delays disease onset and protects motor neurons (*31*). Similar protective effects are noted in Alzheimer’s disease models (*32*). These findings reflect a common mechanism of mitochondrial dysfunction in these otherwise disparate conditions. In traumatic brain injury, CypD knockout or inhibition by cyclosporine helps to ameliorate the increased sensitivity to increased Ca^2+^ induced by the injury (*6*). Specific CypD localization has been noted in the brain, especially in the mitochondria of GABAergic neurons and motor neurons. In astrocytes, CypD was noted in NG2-expressing cells, which include oligodendrocyte precursor cells (*9*). In non-CNS indications, CypD inhibition is also protective in animal models of liver fibrosis (*33*) and muscular dystrophy (*29*). Our molecules are selective for CypD over CypA at the cellular level, produced by the mitochondrial concentration but not the protein level as assessed by our binding assays.

The quinolinium-cyclosporine CNS agents described here are outside of normal CNS physicochemical space as defined by the multiparameter score. Quaternary salts were specifically excluded by Lipinski *et al*. (*3*). Our molecules have low or undetectable permeability in Caco-2 cells yet penetrate mitochondrial membranes rapidly and induce their biological effects in seconds. Similarly, mitochondrial-specific dyes enter cells and quickly stain the organelles indicating a disconnect between the permeability assays and action in cells. Historical ideas of the Lipinski rule of five (which specifically excluded many natural products and quaternary salts) have been modified to include many large macrocyclic natural products, as described in the bRo5 concept (*4*). For passive diffusion, conformational flexibility allows macrocycles to adjust to changing environments in membranes and plasma or intracellular space (*34*).

For our delocalized quaternary salts, the mechanism of translocation across membranes remains to be determined, endocytosis, transporter-mediated access, or specific carrier molecules could be involved. The organic cation transporters are well known as transporters of cationic drugs (*35*) but no delocalized systems have been reported to our knowledge. Delocalization enabled greater mitochondrial activity and better CNS penetration, note the difference between JW47, where the charge is not delocalized, and the delocalized versions JW76 and JP1-138. Although we achieved higher levels in the brain than those reported for cyclosporine in mice at similar doses, we recognize that P-glycoprotein expression limits the brain penetration of cyclosporine (*28*, *36*). We assume the electromotive force to be important for activity as the compounds had low passive permeability. A flip-flop mechanism has been suggested for triphenylphosphonium cations with a dependence on the voltage difference (*37*).

Some natural products such as the alkaloid berberine are quaternary ammonium salts but have been observed to distribute into the brain and induce CNS effects (*38*). Berberine and some related analogs have low but detectable PAMPA permeability (1.0 × 10^−6^ to 1.2 × 10^−6^ cm s^−1^) and Caco-2 cell line permeability (AP-BL 2.59 × 10^−6^ cm s^−1^ and BL-AP 25.16 × 10^−6^ cm s^−1^) (*39*). Other quaternary salts are useful as orally dosed drugs such as the PCSK9 inhibitors developed by Merck exemplified by MK-0616, a tricyclic peptide macrocycle with high polarity and a molecular weight of approximately 1600. Although permeation enhancers were required to improve oral absorption, the picomolar potencies of these drugs aid efficacy (*40*).

In summary, we investigated charge delocalization of the quinolinium moiety in cyclosporine macrocyclic peptide analogs. These studies identified JW76 and JP1-138 as potent inhibitors of CypD, highly active in blocking permeability transition pore opening in isolated mitochondria. The pharmacokinetic and metabolism studies showed that JP1-138 achieved measurable brain levels for 48 hours following a single dose with brain AUCs of approximately 2% of plasma levels and approximately 20-fold greater brain concentrations than our prototype compound or those reported for cyclosporine. We anticipate that application of this technology to other macrocyclic drugs could also enable their passage into the CNS.

## MATERIALS AND METHODS

Complete details of materials and methods are provided in the Supplementary Materials.

## Supplementary Materials

### This PDF file includes:

Figs. S1 to S6 Tables S1 to S7 Supplementary Materials and Methods References
