# Supplementary material for: Delocalized quinolinium-macrocyclic peptides, an atypical chemotype for CNS penetration
Source: Sci Adv. 2024 Jul 10;10(28):eado3501. doi: 10.1126/sciadv.ado3501 (PMC11235165; doi:10.1126/sciadv.ado3501)
Supplement: Supplementary file 1 — Figs. S1 to S6 Tables S1 to S7 Supplementary Materials and Methods References [file sciadv.ado3501_sm.pdf]

Science Advances
AAAS

Supplementary Materials for

Delocalized quinolinium-macrocyclic peptides, an atypical chemotype for CNS penetration

Valeria Pingitore *et al.*

Corresponding author: David L. Selwood, d.selwood@ucl.ac.uk

Sci. Adv. **10**, eado3501 (2024)
DOI: 10.1126/sciadv.ado3501

This PDF file includes:

Figs. S1 to S6
Tables S1 to S7
Supplementary Materials and Methods
References

Supplementary Figures

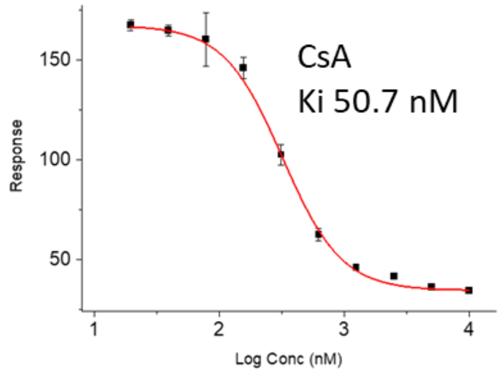

Figure S1 CypD binding of Cyclosporin A. Determined using a fluorescence polarization assay with a Biotinylated-CsA probe.

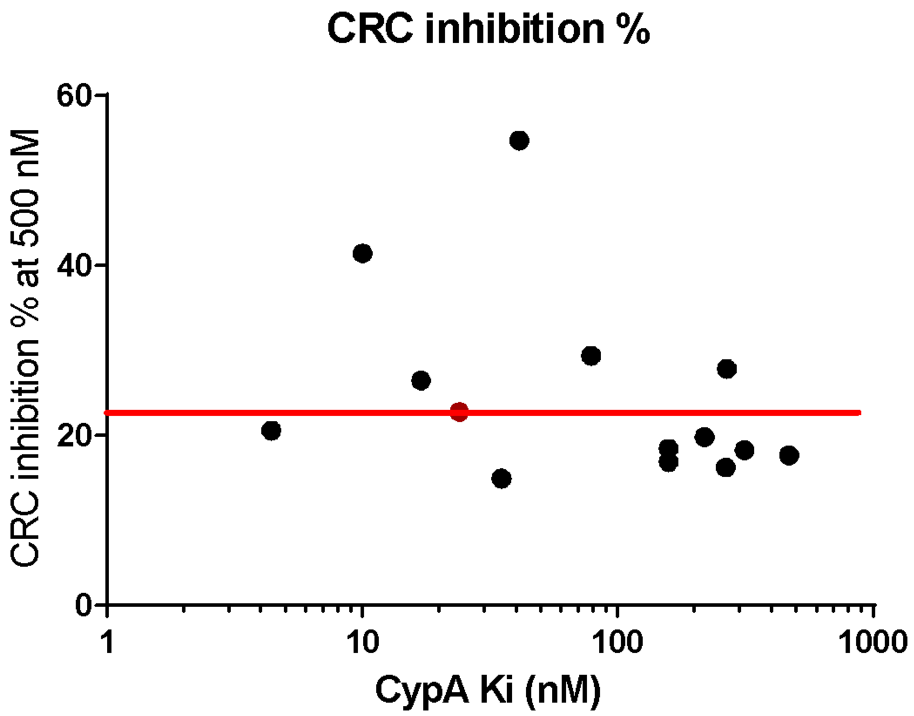

Figure S2. CRC % inhibition in rat liver mitochondria relative to CsA. Treated-NonTreated)/ NonTreated)*100

Supplementary Tables

Supplementary tables

Table S1. Fractional contributions of substituents to physicochemical properties of cyclosporin A.

ID	ID	Structure	Molecular weight	tPSA	cLogP
	CsA[a]	_	0 [1202]	0 [280]	0 [3.6]
	JP1-068	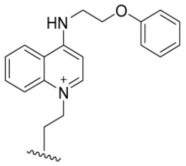	361	24.3	1.3
	JW76	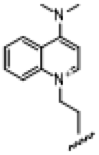	201	6.3	-1.33
	JW28	P^+Ph_3	291	0	6.7
	JW47	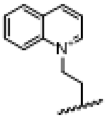	158	3	-2.2

[a] Properties for cyclosporin A given in parenthesis.

Table S2. Cyclophilin A binding affinity for the 4-oxa substituted quinolinium salts and O-linked quinolinium salts using fluorescence polarization.

Quinolinium salt generic structure	ID	R	Ki (nM)
	CsA		22.6 ± 2.6
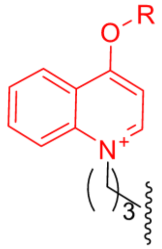	TWH32	Me	78.6 ± 5.1
	TWH30	CH2CH2Ph	267 ± 16
	TWH36	CH2CH2CH2Ph	313 ± 109
	TWH44	CH2CH2CH2(4-OMePh)	158 ± 17
	TWH53	CH2Ph	158 ± 43
	TWH68	CH2CH2OPh	467 ± 85
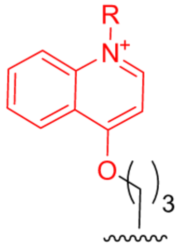	TWH46	CH2CH2Ph	265 ± 111
	TWH43	CH2CH2CH2Ph	218 ± 57

Table S3. CypA binding affinity for 4-aza substituted quinolinium salts using Fluorescence polarization.

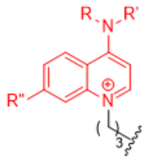

ID	R	R’	R”	K_i (nM) CypA	K_i (nM) CypD
CsA	-	-	-	22.6 ± 2.6	73 ± 3
JP1-068	CH_2CH_2OPh	H	H	10 ± 4	38.08 ± 3
JP1-028	CH_2Ph	Me	H	35 ± 3	NT
JP1-037	CH_2CH_2Ph	Me	H	4.4 ± 2	NT
JP1-037H	CH_2CH_2Ph	H	H	17 ± 5	NT
JW76	Me	Me	CF_3	41 ± 2	60 ± 5

Table S4. CypA Ki versus percent CRC inhibition, Raw data for plot of Figure S2

	**CypA Ki**	**CRC inhibition %**
CsA	22.6	22.81
JP 1 028	35	14.97
JP 1 037	4.4	20.61
JP 1037H	17	26.53
JP 1068	10	41.45
TWH 030	267	27.89
TWH 032	78.6	29.42
TWH 036	313	18.34
TWH 043	265	16.28
TWH 044	158	16.95
TWH 046	218	19.88
TWH 053	158	18.46
TWH 068	467	17.71
JW76	41	54.7

Table S5. Pharmacokinetic study of selected compounds in mice at two timepoints

**Compound**		**Plasma**			**Brain**			
	**Time (h)**	**(ng/mL)**			**Time (h)**	**ng/mL**		
JW76	1	50800	±	3044.7a	1	668.1	±	276.3
	4	25033.3	±	10263	4	545.3	±	98.1
JP1-068	1	6410	±	702.4	1	110.1	±	48.2
	4	6253.3	±	2162.7	4	76.0	±	22.0
JW47	2	1374	±	384	2	18	±	1.9
	4	4900	±	850	4	17	±	1.6

Table S6. hERG activity, for selected analogues

Compound	hERG (IC_{50})
Cyclosporin A	3-4 µM (lit)
JP1-068	1-2 µM
JW76	16.3% at 25µM

Table S7. Focused SAR set based on compound JW76.

Compound	R	R”	CypA	CypD	CRC
			K_i (nM)	K_i (nM)	% inhibition at 10 nM
JW76	NMe_2	CF_3	41 ± 2	60 ± 5	32.9
JP1-180	NMe_2	H	21 ± 1.5	77 ± 46	37.8
JP1-138	morpholine	CF_3	42 ± 2.2	64 ± 16	33.6
JP1-141	morpholine	H	29 ± 1.5	93 ± 2.4	33.5
JP1-159	pyrrolidine	H	104 ± 11	96 ± 4	-
JP1-140	pyrrolidine	CF_3	58 ± 5	76 ± 8	-
JP1-166	piperidine	H	31 ± 3	75 ± 1.4	46.0
JP1-164	piperidine	CF_3	45 ± 5	85 ± 5.5	41.6

Chemistry. Synthesis and characterization of the new compounds

Commercially available starting materials were used as supplied without further purification. Reactions were carried out in dry solvents (dichloromethane (DCM), N,N-dimethylformamide (DMF), THF, cyclobutanol, cyclopentanol, isopropanol) unless otherwise noted. Reactions were monitored by thin-layer chromatography (TLC) and an Agilent 6100 single quadrupole LC 1200 series mass spectrometer. Purification was carried out on either a Biotage Isolera one or Biotage Isolera four system, with either C4, C18 or silica gel prepacked columns. ^{1}H and ^{13}C NMR data were recorded on a 600 MHz Bruker Avance III with a 5 mm helium-cooled cryoprobe. Chemical shifts for ^{1}H and ^{13}C spectra were referenced

to residual solvent. Mass spectra were obtained on an Agilent 1200 liquid chromatography system connected to an Agilent 6100 single quadrupole mass spectrometer. High-resolution mass spectrometry-electrospray ionization (HRMS-ESI) data were obtained on an Agilent 1200 liquid chromatography system connected to an Agilent 6510 QTOF mass spectrometer. Purity was determined by liquid chromatography−mass spectrometry (LCMS) analysis and is >95% for all compounds unless otherwise stated.

Synthesis of 4-oxa substituted quinolinium intermediates.

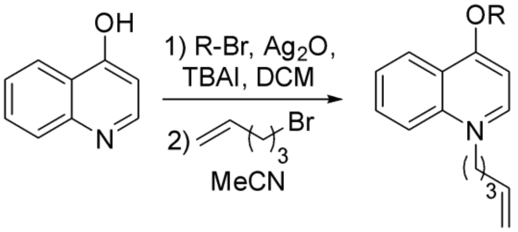

Figure S3. Preparation of 4-oxa substituted quinolinium intermediates.

Alkylation of 4-quinolinol general procedure

Ag_2O (3 eq), TBAI (0.1 eq) and alkyl bromide (2 eq) were added to a solution of 4-quinolinol (1 eq) in DCM and stirred under an atmosphere of N_2, in the absence of light. After being stirred for 2 days the reaction was filtered through Celite, which was washed with DCM and the combined filtrates concentrated *in vacuo*. The residue was purified by flash column chromatography on silica gel, eluting with 5% MeOH in DCM (>95% pure by HPLC).

4-Phenethoxyquinoline

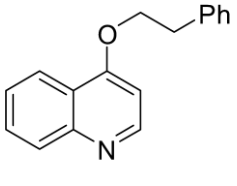

4-quinolinol (500 mg, 3.44 mmol) in DCM (10 mL) was treated with 2-(bromoethyl)benzene (0.92 mL, 6.88 mmol) using the general procedure above to give 4-phenethoxyquinoline (502 mg, 59%) as a colourless oil. Prepared in the literature (*41*).

^{1}H-NMR (600 MHz, $CDCl_3$) δ 8.72 (1H, d, *J* 5.2, 2-H), 8.20 (1H, ddd, *J* 8.3, 1.5, 0.6, 5-H or 8-H), 8.03 (1H, ddd, *J* 8.5, 1.2, 0.6, 5-H or 8-H), 7.69 (1H, ddd, *J* 8.4, 6.9, 1.5, 6-H or 7-H), 7.50 (1H, ddd, *J* 8.2, 6.8, 1.2, 6-

H or 7-H), 7.36 (2H, s, Ar-H), 7.35 (2H, s, Ar-H), 7.27 (1H, dt, *J* 8.9, 4.5), 6.70 (1H, d, *J* 5.2, 3-H), 4.39 (2H, t, *J* 6.8, OC<u>H</u>$_2$CH$_2$Ph), 3.26 (2H, t, *J* 6.8, OCH$_2$C<u>H</u>$_2$Ph).
^{13}C-NMR (150 MHz, CDCl$_3$) δ 161.51, 151.41, 149.31, 137.83, 129.86, 129.13, 128.95, 128.76, 126.89, 125.71, 121.99, 121.53, 100.75, 69.17, 35.59.

4-(3-phenylpropoxy)quinoline

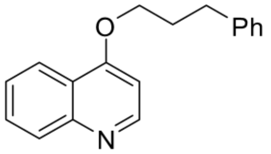

4-quinolinol (500 mg, 3.44 mmol) in DCM (10 mL) was treated with 1-bromo-3-phenylpropane (1.05 mL, 6.88 mmol) using the general procedure above to give the product (567 mg, 63%) as a colourless oil. Reported in the literature (*2*).

^{1}H-NMR (600 MHz, CDCl$_3$) δ 8.72 (1H, d, *J* 5.2, 2-H), 8.23 (1H, ddd, *J* 8.4, 1.5, 0.6, 5-H or 8-H), 8.04 (1H, ddd, *J* 8.4, 1.1, 0.7, 5-H or 8-H), 7.71 (1H, ddd, *J* 8.4, 6.9, 1.5 Hz, 6-H or 7-H), 7.52 (1H, ddd, *J* 8.2, 6.8, 1.2 Hz, 6-H or 7-H), 7.32 – 7.29 (2H, m, Ar-H), 7.25 – 7.20 (3H, m, Ar-H), 6.68 (1H, d, *J* 5.2 Hz, 3H), 4.19 (2H, t, *J* 6.2, OC<u>H</u>$_2$CH$_2$CH$_2$Ph), 2.93 (2H, t, *J* 7.5, OCH$_2$CH$_2$C<u>H</u>$_2$Ph), 2.29 (2H, ddt, *J* 8.4, 7.3, 6.2, OCH$_2$C<u>H</u>$_2$CH$_2$Ph).
^{13}C-NMR (150 MHz, CDCl$_3$) 161.71, 151.50, 149.32, 141.14, 129.90, 129.02, 128.70, 128.66, 126.29, 125.73, 121.98, 121.63, 100.79, 67.43, 32.32, 30.57.

4-(3-(4-methoxyphenyl)propoxy)quinoline

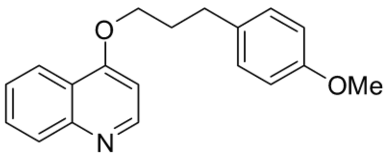

4-quinolinol (200 mg, 1.38 mmol) in DCM (5 mL) was treated with 1-(3-bromopropyl)-4-methoxybenzene (0.48 mL, 2.76 mmol) using the general procedure above to give 4-(3-(4-methoxyphenyl)propoxy)quinoline (211 mg, 52%) as a colourless oil.

^{1}H-NMR (600 MHz, CDCl$_3$) δ 8.72 (1H, d, *J* 5.2 Hz, 2-H), 8.23 (1H, ddd, *J* 8.3, 1.5, 0.6, 5-H or 8-H), 8.04 (1H, ddd, *J* 8.5, 1.2, 0.6, 5-H or 8-H), 7.71 (1H, ddd, *J* 8.4, 6.9, 1.5, 6-H or 7-H), 7.52 (1H, ddd, *J* = 8.2, 6.8, 1.2, 6-H or 7-H), 7.14 (2H, d, *J* 8.8, Ar-H), 6.84 (2H, d, *J* 8.7 Hz, Ar-H), 6.67 (1H, d, *J* 5.2 Hz, 3-H), 4.17 (2H, t, *J*

= 6.2 Hz, OC$\underline{H}_2$CH$_2$CH$_2$Ph), 3.79 (3H, s, OMe), 2.86 (2H, t, *J* 7.5, OCH$_2$CH$_2$C$\underline{H}_2$Ph), 2.27 – 2.20 (2H, m, OCH$_2$C$\underline{H}_2$CH$_2$Ph).
^{13}C-NMR (150 MHz, CDCl$_3$) δ 161.68, 158.09, 151.54, 149.31, 133.10, 129.87, 129.56, 129.01, 125.68, 121.97, 121.60, 114.05, 100.78, 67.34, 55.39, 31.34, 30.74.

4-(benzyloxy)quinoline

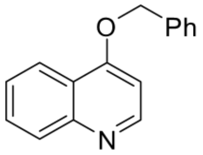

4-quinolinol (200 mg, 1.38 mmol) in DCM (10 mL) was treated with benzyl bromide (0.33 mL, 2.76 mmol) using the general procedure above to give 4-(benzyloxy)quinoline (256 mg, 79%) as a colourless oil. Literature (*42*).

^{1}H-NMR (600 MHz, CDCl$_3$) δ 8.39 (1H, dd, *J* 8.3, 1.6), 7.60 (1H, d, *J* 7.7), 7.45 (1H, ddd, *J* 8.7, 7.0, 1.7), 7.29 – 7.22 (5H, m, Ar-H), 7.09 – 7.06 (2H, m), 6.26 (1H, d, *J* 7.7), 5.26 (2H, C$\underline{H}_2$Ph, s).
^{13}C-NMR (150 MHz, CDCl$_3$) δ 178.43, 144.01, 140.16, 135.29, 132.32, 129.29, 128.35, 127.35, 126.90, 126.18, 123.85, 116.38, 110.31, 56.55.

4-(2-phenoxyethoxy)quinoline

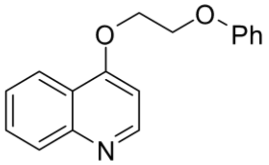

4-quinolinol (200 mg, 1.38 mmol) in DCM (10 mL) was treated with β-bromophenetole (555 mL, 2.76 mmol) using the general procedure above to give 4-(2-phenoxyethoxy)quinoline (75 mg, 21%) as a colourless oil.

^{1}H-NMR (600 MHz, CDCl$_3$) δ 8.76 (1H, d, *J* 5.1 Hz, 2-H), 8.21 (1H, ddd, *J* 8.4, 1.5, 0.6, 5-H or 8-H), 8.04 (1H, ddd, *J* 8.5, 1.1, 0.6, 5-H or 8-H), 7.70 (1H, ddd, *J* 8.4, 6.9, 1.5 Hz, 6-H or 7-H), 7.49 (1H, ddd, *J* 8.2, 6.8, 1.2, 6-H or 7-H), 7.35 – 7.31 (2H, m, Ar-H), 7.03 – 6.98 (3H, m, Ar-H), 6.77 (1H, d, *J* 5.2, 3-H), 4.53 (2H, dd, *J* 5.7, 3.2, OC$\underline{H}_2$), 4.47 (2H, dd, *J* 6.2, 3.5, OC$\underline{H}_2$).
^{13}C-NMR (150 MHz, CDCl$_3$) δ 161.44, 158.62, 151.40, 149.35, 130.02, 129.76, 128.94, 125.84, 122.13, 121.49, 121.44, 114.83, 100.84, 67.11, 66.14.

4-Methoxyquinoline

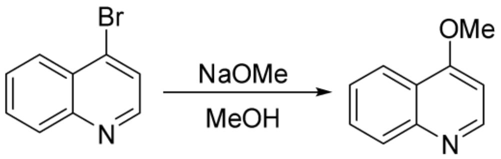

NaOMe (650 mg, 12.02 mmol) was added to a solution of 4-bromoquinoline (500 mg, 2.4 mmol) in MeOH (10 mL) and heated to reflux overnight. The mixture was cooled to RT and the methanol remove *in vacuo*. The residue was taken up in EtOAc (30 mL) and washed with water (30 mL), the combined organic phase was dried over $MgSO_4$, filtered and concentrated *in vacuo* to afford 4-methoxyquinoline (380 mg, 99%) as a white solid. Literature (*43*).

^{1}H-NMR (600 MHz, $CDCl_3$) δ 8.70 (1H, d, *J* 5.2, 2-H), 8.16 (1H, ddd, *J* 8.3, 1.5, 0.7, 5-H or 8H), 8.00 (1H, dt, *J* 8.4, 0.9, 5-H or 8H), 7.65 (1H, ddd, *J* 8.5, 6.9, 1.5, 6-H or 7-H), 7.46 (1H, ddd, *J* 8.2, 6.9, 1.2, 6-H or 7-H), 6.68 (1H, d, *J* 5.2, 3-H), 3.98 (3H, s, OMe).

Quinolinium formation general procedure

Alkyl bromide (1.1 eq) was added to a solution of alkylated quinoline (1 eq) in MeCN and heated to reflux in a sealed vessel for 2 days. The reaction was cooled to RT and concentrated *in vacuo*. The residue was purified by flash column chromatography on silica gel, eluting with 5% MeOH in DCM.

4-Phenethoxyquinoline-1-(pent-4-en-1-yl) quinolinium bromide

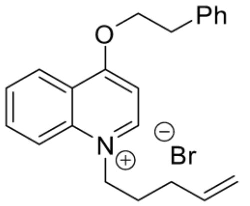

4-penethoxyquinoline (100 mg, 0.40 mmol) in MeCN (1 mL) was treated with 5-bromo-1-pentene (0.052 mL, 0.44 mmol) using the general procedure above to give 4-phenethoxyquinoline-1-(pent-4-en-1-yl)quinolinium bromide (88 mg, 55%) as a colourless oil.

^{1}H-NMR (600 MHz, $CDCl_3$) δ 10.09 (1H, d, *J* 7.1, 2-H), 8.36 (1H, dt, *J* 8.2, 1.1), 8.12 – 8.07 (2H, m), 7.79 (1H, ddd, *J* 8.2, 6.1, 2.0), 7.67 (1H, d, *J* 7.1, 3-H), 7.33 – 7.28 (4H, m, Ar-H), 7.25 – 7.20 (1H, m), 5.76 (1H, ddt, *J* 16.9, 10.2, 6.6, C$\underline{H}$=CH_2), 5.10 – 4.97 (4H, m, CH=C$\underline{H}_2$ and NC$\underline{H}_2$), 4.76 (2H, t, *J* 6.4, OC$\underline{H}_2CH_2$Ph), 3.29 (2H, t, *J* 6.4, OCH_2C$\underline{H}_2$Ph), 2.28 – 2.20 (2H, m, NCH_2C$\underline{H}_2$), 2.14 – 2.05 (2H, m, C$\underline{H}_2$CH=CH_2).

4-(3-phenylpropoxy)-1-(pent-4-en-1-yl)quinolinium bromide.

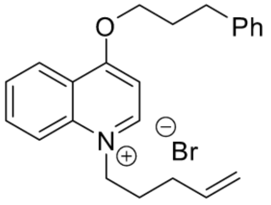

4-(3-phenylpropoxy)quinoline (100 mg, 0.38 mmol) in MeCN (1 mL) was treated with 5-bromo-1-pentene (0.05 mL, 0.42 mmol) using the general procedure above to give 4-(3-phenylpropoxy)-1-(pent-4-en-1-yl)quinolinium bromide (99 mg, 63%) as a colourless oil.

^{1}H-NMR (600 MHz, $CDCl_3$) δ 10.05 (1H, d, *J* 7.1, 2-H), 8.27 (1H, dd, *J* 8.2, 1.3), 8.14 – 8.07 (2H, m), 7.78 (1H, ddd, *J* 8.1, 6.5, 1.4), 7.63 (1H, d, *J* 7.1, 3-H), 7.26 – 7.23 (2H, m, Ar-H), 7.19 – 7.14 (3H, m), 5.76 (1H, ddt, *J* 16.9, 10.2, 6.6, $C\underline{H}=CH_2$), 5.08 – 4.97 (4H, m, $CH=C\underline{H}_2$ and $NC\underline{H}_2$), 4.53 (2H, t, *J* 6.4, $OC\underline{H}_2CH_2CH_2Ph$), 3.29 (2H, t, *J* 7.5, $OCH_2CH_2C\underline{H}_2Ph$), 2.31 (2H, ddt, *J* 12.8, 7.8, 6.3, $NCH_2C\underline{H}_2$), 2.24 (2H, tdd, *J* 7.6, 3.7, 1.4, $OCH_2C\underline{H}_2CH_2Ph$), 2.12 – 2.05 (2H, m, $C\underline{H}_2CH=CH_2$).
^{13}C-NMR (150 MHz, $CDCl_3$) δ 168.10, 152.26, 140.46, 138.47, 136.70, 136.19, 135.49, 128.79, 128.51, 126.44, 124.89, 121.88, 118.02, 116.85, 103.46, 71.66, 55.86, 32.24, 30.40, 28.90, 27.65.

4-(3-(4-methoxyphenyl)propoxy)-1-(pent-4-en-1-yl)quinolinium bromide

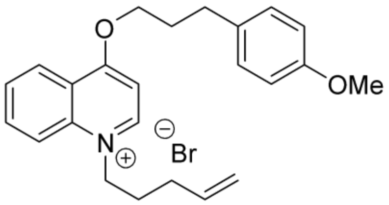

4-(3-(4-methoxyphenyl)propoxy)quinoline (100 mg, 0.34 mmol) in MeCN (1 mL) was treated with 5-bromo-1-pentene (0.044 mL, 0.38 mmol) using the general procedure above to give 4-(3-(4-methoxyphenyl)propoxy)-1-(pent-4-en-1-yl)quinolinium bromide (56 mg, 37%) as a colourless oil.

4-Benzyloxy-1-(pent-4-en-1-yl)quinolinium bromide

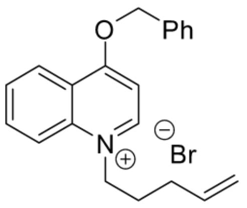

4-Benzyloxyquinoline (200 mg, 0.85 mmol) in MeCN (2 mL) was treated with 5-bromo-1-pentene (0.11 mL, 0.94 mmol) using the general procedure above to give 4-benzyloxyoxyquinoline-1-(pent-4-en-1-yl)quinolinium bromide (268 mg, 83%) as a colourless oil.

^{1}H-NMR (600 MHz, $CDCl_3$) δ 10.36 (1H, d, *J* 6.1, 2H), 8.44 (1H, dd, *J* 8.5, 1.6, 5-H or 8-H), 8.13 – 8.09 (1H, m, 5-H or 8-H), 7.97 (1H, ddd, *J* 8.7, 7.0, 1.5, 6-H or 7-H), 7.77 (1H, dd, *J* 8.5, 7.1, 6-H or 7-H), 7.68 (1H, d, *J* 7.0, 3H), 7.33 – 7.27 (5H, m, Ar-H), 6.31 (2H, s, $OC\underline{H}_2Ph$), 5.86 (1H, ddt, *J* 17.0, 10.2, 6.6, $C\underline{H}=CH_2$), 5.10 (1H, dd, *J* 17.2, 1.7, $CH=C\underline{H}_{trans}$), 5.07 (1H, dd, *J* 9.8, 1.7, $CH=C\underline{H}_{cis}$), 4.57 (2H, t, *J* 6.3, $NC\underline{H}_2$), 2.37 – 2.32 (2H, m, $C\underline{H}_2CH=CH_2$), 2.13 (2H, p, *J* 6.7, $NCH_2C\underline{H}_2$).
^{13}C-NMR (150 MHz, $CDCl_3$) δ 168.61, 153.12, 138.91, 136.64, 135.36, 133.34, 129.51, 128.81, 127.11, 124.77, 122.05, 118.90, 116.50, 103.34, 71.71, 59.78, 50.89, 29.98, 27.67.

4-(2-phenoxyethoxy)-1-(pent-4-en-1-yl)quinolinium bromide

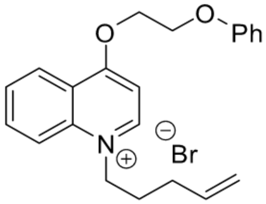

4-(2-phenoxyethoxy)quinoline (65 mg, 0.25 mmol) in MeCN (0.5 mL) was treated with 5-bromo-1-pentene (0.032 mL, 0.27 mmol) using the general procedure above to give 4-(2-phenoxyethoxy)-1-(pent-4-en-1-yl)quinolinium bromide (23 mg, 22%) as a colourless oil.

^{1}H-NMR (600 MHz, $CDCl_3$) δ 10.03 (1H, d, *J* 7.1, 2-H), 8.41 (1H, dd, *J* 8.4, 1.4), 8.13 – 8.06 (2H, m), 7.95 (1H, d, *J* 7.1, 3-H), 7.77 (1H, ddd, *J* 8.2, 6.7, 1.2), 7.28 – 7.24 (2H, m, Ar-H), 6.95 – 6.91 (3H, m, Ar-H), 5.78 (1H, ddt, *J* 16.9, 10.2, 6.7, $C\underline{H}=CH_2$), 5.08 – 4.98 (6H, m, $CH=C\underline{H}_2$, $OC\underline{H}_2CH_2Ph$ and NCH_2), 4.53 (2H, dt, *J* 6.2, 2.0, $OCH_2C\underline{H}_2Ph$), 2.28 – 2.23 (2H, m, $NCH_2C\underline{H}_2$), 2.11 (2H, qd, *J* 8.0, 6.3, $C\underline{H}_2CH=CH_2$).
^{13}C-NMR (150 MHz, $CDCl_3$) δ 167.98, 158.19, 152.08, 138.41, 136.06, 135.37, 129.66, 128.72, 125.12, 121.86, 121.50, 117.82, 116.82, 114.67, 104.03, 70.99, 65.52, 55.83, 30.31, 28.83.

4-methoxy-1-(pent-4-en-1-yl)quinolinium bromide

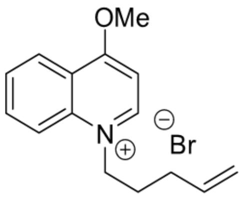

4-Methoxyquinoline (100 mg, 0.63 mmol) in MeCN (1 mL) was treated with 5-bromo-1-pentene (0.11 mL, 0.69 mmol) using the general procedure above to give 4-methoxy-1-(pent-4-en-1-yl)quinolinium bromide (153 mg, 79%) as a colourless oil.

^{1}H-NMR (600 MHz, $CDCl_3$) 10.40 (1H, dd, *J* 7.0, 0.6, 2-H), δ 8.49 (1H , dt, *J* 8.4, 1.1), 8.14 – 8.12 (2H, m), 7.90 – 7.84 (1H, m), 7.54 (1H, d, *J* 7.0, 3-H), 5.86 (1H, ddt, *J* 16.9, 10.2, 6.7, C$\underline{H}$=CH_2), 5.12 – 5.05 (2H, m, CH=C$\underline{H}_2$), 4.70 (3H, s, OMe), 4.55 (2H, t, *J* 6.4, NC$\underline{H}_2$), 2.35 (2H, dtt, *J* 7.9, 6.6, 1.4, C$\underline{H}_2$CH=CH_2), 2.14 (2H, dq, *J* 7.8, 6.4, NCH_2C$\underline{H}_2$).
^{13}C-NMR (150 MHz, $CDCl_3$) δ 168.26, 153.32, 139.48, 136.56, 135.47, 128.98, 124.73, 121.68, 117.95, 116.55, 103.02, 71.47, 44.15, 29.96, 27.64.

Synthesis of the 4-oxa linked quinolinium intermediates.

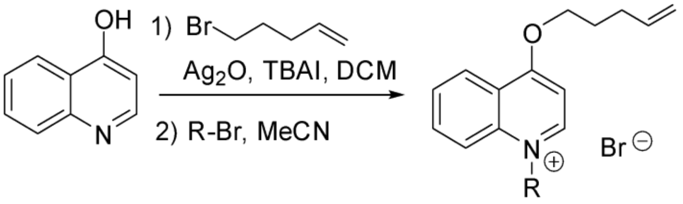

Figure S4. Synthesis of the 4-oxa linked quinolinium intermediates.

4-(pent-4-en-1-yloxy)quinoline

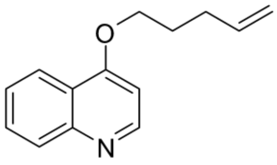

4-quinolinol (200 mg, 1.38 mmol) in DCM (5 mL) was treated with 5-bromo-1-pentene (0.33 mL, 2.76 mmol), Ag_2O (4.14 mmol, 960 mg), TBAI (0.14 mmol, 51 mg) and stirred under an atmosphere of N_2, in the absence of light. After being stirred for 2 days the reaction was filtered through Celite®, which was washed with DCM and the combined filtrates concentrated in vacuo. The residue was purified by flash column chromatography on silica gel, eluting with 5% MeOH/DCM to give 4-(pent-4-en-1-yloxy)quinoline (144 mg, 49%) as a colourless oil.

^{1}H-NMR (600 MHz, $CDCl_3$) δ 8.71 (1H, d, *J* 5.2, 2-H), 8.21 (1H, ddd, *J* 8.3, 1.5, 0.7, 5-H or 8-H), 8.02 (1H, dt, *J* 8.5, 0.9, 5-H or 8-H), 7.68 (1H, ddd, *J* 8.4, 6.9, 1.5, 6-H or 7-H), 7.49 (1H, ddd, *J* 8.2, 6.8, 1.2, 6-H or 7-H), 6.69 (1H, d, *J* 5.3 Hz, 3-H), 5.88 (1H, ddt, *J* 16.9, 10.2, 6.7, $C\underline{H}=CH_2$), 5.08 (1H, dq, *J* 17.1, 1.7, $CH=C\underline{H}_{trans}$), 5.03 (1H, ddt, *J* 10.2, 2.2, 1.2, $CH=C\underline{H}_{cis}$), 4.18 (2H, t, *J* 6.3, $OC\underline{H}_2CH_2CH_2$), 2.33 (2H, tdd, *J* 7.9, 6.1, 1.4, $OCH_2CH_2C\underline{H}_2$), 2.07 – 2.00 (2H, m, $OCH_2C\underline{H}_2CH_2$).
^{13}C-NMR (150 MHz, $CDCl_3$) δ 161.71, 151.44, 149.19, 137.46, 129.84, 128.86, 125.63, 121.95, 121.56, 115.74, 100.73, 67.66, 30.21, 28.11.

4-(pent-4-en-1-yloxy)-1-(phenethyl)quinolinium bromide

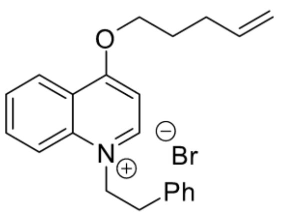

4-(pent-4-en-1-yloxy)quinoline (50 mg, 0.23 mmol) in MeCN (0.5 mL) was treated with 2-(bromoethyl)benzene (0.035 mL, 0.26 mmol) using the general procedure above to give 4-(pent-4-en-1-yloxy)-1-(phenethyl)quinolinium bromide (68 mg, 74%) as a colourless oil.

^{1}H-NMR (600 MHz, $CDCl_3$) δ 9.83 (1H, dd, *J* 7.1, 1.1, 2-H), 8.43 (1H, dd, *J* 8.4, 1.4), 8.17 (1H, d, *J* 8.9 Hz), 8.09 (1H, ddd, *J* 8.8, 7.0, 1.5), 7.81 (1H, ddd, *J* 8.2, 7.0, 0.9), 7.35 (1H, d, *J* 7.1, 3-H), 7.21 – 7.10 (5H, m, Ar-H), 5.83 (1H, ddt, *J* 16.9, 10.2, 6.7, $CH=CH_2$), 5.34 (2H, t, *J* 7.3, NCH_2), 5.09 – 5.02 (2H, m, $CH=CH_2$), 4.47 (2H, t, *J* 6.3, OCH_2), 3.35 (2H, t, *J* 7.2, CH_2Ph), 2.33 – 2.29 (2H, m, OCH_2CH_2), 2.09 (2H, dt, *J* 8.0, 6.5, $CH_2CH=CH_2$).
^{13}C-NMR (150 MHz, $CDCl_3$) δ 168.08, 152.20, 138.53, 136.64, 135.68, 135.44, 129.12, 129.09, 128.78, 127.59, 124.81, 121.76, 118.09, 116.42, 102.75, 71.48, 57.23, 35.96, 29.94, 27.62.

4-(pent-4-en-1-yloxy)-1-(3-phenylpropyl)quinolinium bromide

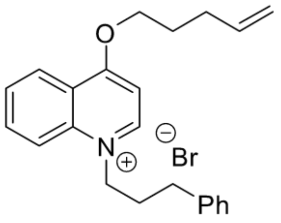

4-(pent-4-en-1-yloxy)quinoline (40 mg, 0.187 mmol) in MeCN (0.5 mL) was treated with 1-bromo-3-phenylpropane (0.031 mL, 0.21 mmol) using the general procedure above to give 4-(pent-4-en-1-yloxy)-1-(3-phenylpropyl)quinolinium bromide (51 mg, 66%) as a colourless oil.

^{1}H-NMR (600 MHz, $CDCl_3$) δ 10.24 (1H, d, *J* 7.1, 2-H), 8.26 (1H, dd, *J* 8.4, 1.4), 7.98 (1H, m), 7.82 (1H, dd, *J* 8.9, 4.5), 7.77 (1H, ddd, *J* 8.1, 6.5, 0.9), 7.53 (1H, d, *J* 7.1, 3-H), 7.30 – 7.27 (1H, m, Ar-H), 7.24 – 7.13 (4H, m), 5.84 (1H, ddt, *J* 16.9, 10.2, 6.7, $C\underline{H}=CH_2$), 5.11 – 5.03 (4H, m, $CH=C\underline{H}_2$ and $NC\underline{H}_2$), 4.53 (2H, t, *J* 6.3, $OC\underline{H}_2CH_2CH_2CH=CH_2$), 2.89 (2H, t, *J* 7.5, $NCH_2CH_2C\underline{H}_2Ph$), 2.39 – 2.30 (4H, m, $NCH_2C\underline{H}_2$ and $OCH_2C\underline{H}_2CH_2$), 2.10 – 2.05 (2H, m, $C\underline{H}_2CH=CH_2$).
^{13}C-NMR (150 MHz, $CDCl_3$) δ 167.98, 152.47, 140.40, 139.94, 138.40, 136.65, 135.28, 128.75, 128.52, 126.57, 124.84, 121.83, 117.85, 116.42, 103.29, 71.54, 55.60, 32.49, 31.20, 30.01, 27.64.

Synthesis of the 4-aza substituted quinolinium intermediates

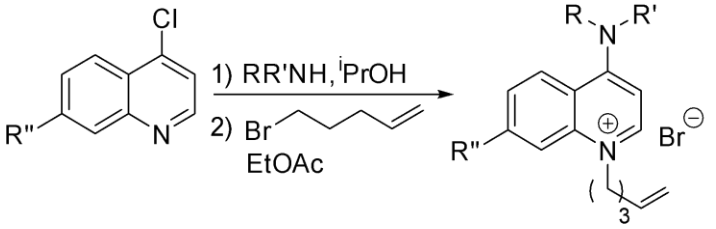

Figure S5. Synthesis of 4-aza substituted quinolinium intermediate.

4-chloroquinoline *N*-substitution general procedure
The secondary amine (2 eq.) was added to a solution of 4-chloroquinoline (1 eq.) in ^{i}PrOH (10 mL) under an atmosphere of N_2, and refluxed for 48 h. After cooling, the solvent was evaporated and the crude product purified by flash column chromatography on silica gel, eluting with EtOAC 0% to 100% in Cy obtaining the final compounds with a final yield between 30% and 70% (>95% pure by HPLC).

N-(2-phenoxyethyl)quinolin-4-amine

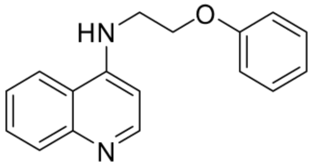

LCMS: expected 264.33, found $[M+H]^+$ 265.22

N-benzyl-N-methylquinolin-4-amine

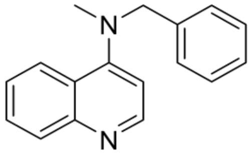

Known compound (*44*).

^{1}H-NMR (600 MHz, CDCl3) δ 8.69 (d, J = 5.0 Hz, 1H), 8.10 (dd, J = 8.5, 0.9 Hz, 1H), 8.08 – 8.06 (m, 1H), 7.64 (ddd, J = 8.3, 6.8, 1.3 Hz, 1H), 7.41 (ddd, J = 11.4, 6.3, 2.9 Hz, 1H), 7.36 – 7.28 (m, 1H), 6.82 (d, J = 5.1 Hz, 1H), 4.51 (s, 2H), 2.90 (s, 3H).

^{13}C-NMR (151 MHz, CDCl3) δ 157.11, 150.64, 149.86, 137.59, 130.11, 129.07, 128.80, 127.66, 127.56, 125.09, 123.94, 123.31, 108.56, 60.45, 40.31.

N-methyl-N-phenethylquinolin-4-amine

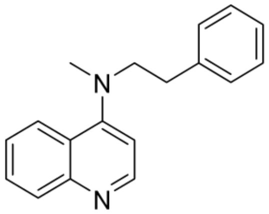

Literature (*44*).

^{1}H-NMR (600 MHz, CDCl3) δ 8.68 (d, J = 5.1 Hz, 1H), 8.07 (dd, J = 8.4, 0.9 Hz, 1H), 7.95 (dd, J = 8.5, 1.0 Hz, 1H), 7.63 (ddd, J = 8.3, 6.8, 1.4 Hz, 1H), 7.42 (ddd, J = 8.2, 6.8, 1.3 Hz, 1H), 7.31 – 7.25 (m, 2H), 7.24 – 7.19 (m, 1H), 7.19 – 7.15 (m, 2H), 6.79 (d, J = 5.1 Hz, 1H), 3.54 – 3.47 (m, 2H), 3.02 (s, 3H), 3.00 – 2.96 (m, 2H).
^{13}C-NMR (151 MHz, CDCl3) δ 157.00, 150.41, 149.73, 139.02, 129.83, 128.89, 128.72, 128.49, 126.38, 124.76, 124.13, 123.43, 108.60, 57.74, 40.70, 33.78.
LCMS: expected 262.36, found $[M+H]^+$ 263.30

N-phenethylquinolin-4-amine

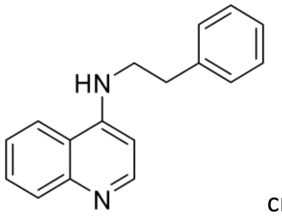

crfk

Literature (*45*).

N,N-dimethyl-7-(trifluoromethyl)quinolin-4-amine

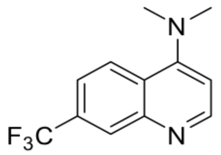

Literature (*46*).

^{1}H-NMR (600 MHz, CDCl3) δ 8.72 (d, J = 6.0 Hz, 1H), 8.32 (s, 1H), 8.16 (d, J = 12.0 Hz, 1H), 7.61 (dd, J= 6.0, J = 12.0, 1H), 6.83 (d, J = 6.0 Hz, 1H), 3.07 (s, 6H).
^{13}C-NMR (600 MHz, CDCl3) δ 156.51, 151.73, 148.93, 131.19 (m), 130.94 (m), 131.68 (m), 130.42 (m) 127.67 (m), 127.65 (m), 127.62 (m), 127.58 (m), 126.03, 125.22, 124.65, 123.05, 120.17 (m), 120.15 (m), 120.12(m), 108.57, 43.97.
LCMS: Expected 240.09, found $[M+H]^+$ 241.10

N,N-dimethylquinolin-4-amine

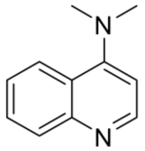

Literature (*47*).

4-(7-(trifluoromethyl)quinolin-4-yl)morpholine

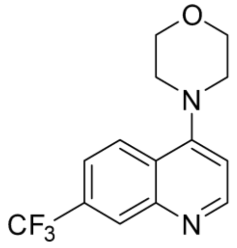

^{1}H-NMR: 1H NMR (600 MHz, CDCl3) δ 8.83 (d, J = 6.0 Hz, 1H), 8.37 (s, 1H), 8.14 (d, J = 6.0 Hz, 1H), 7.66 (dd, J= 6.0, J = 12.0, 1H), 6.95 (d, J = 6.0 Hz, 1H), 4.00 (t, J= 6.0, 4H), 3.25 (t, J= 6.0, 4H).
^{13}C-NMR (600 MHz, CDCl3) δ 156.82, 152.22, 148.73, 131.33 , 131.07, 129.94 (m), 127.91 (m) 125.06, 122.95, 121.22 (m), 121.19 (m), 121.17 (m), 110.28, 66.91, 52.72.
LCMS: Expected 282.10, found $[M+H]^+$ 283.20

4-(quinolin-4-yl)morpholine (*48*)

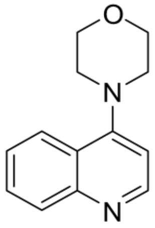

LCMS: Expected 214.27, found $[M+H]^+$ 215.20

4-(pyrrolidin-1-yl)quinoline (*49*).

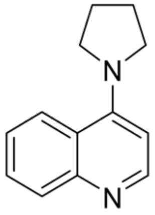

^{1}H-NMR (600 MHz, CDCl3) δ 8.39 (d, J = 8.1 Hz, 1H), 8.33 (d, J = 8.5 Hz, 1H), 8.25 (d, J = 6.8 Hz, 1H), 7.76 – 7.70 (m, 1H), 7.49 (ddd, J = 8.4, 7.0, 1.2 Hz, 1H), 6.52 (d, J = 6.9 Hz, 1H), 3.93 (s, 4H), 2.22 – 2.12 (m, 5H).
LCMS: Expected 198.27, found $[M+H]^+$ 199.20

4-(pyrrolidin-1-yl)-7-(trifluoromethyl)quinoline

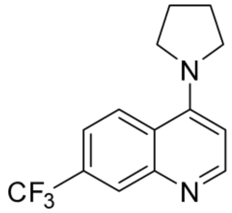

^{1}H-NMR (600 MHz, CDCl3) δ 8.43 (d, J = 8.7 Hz, 2H), 8.34 (d, J = 6.4 Hz, 1H), 7.57 (dd, J = 9.1, 1.6 Hz, 1H), 6.59 (d, J = 6.4 Hz, 1H), 3.88 (t, J = 6.4 Hz, 4H), 2.26 – 2.05 (m, 4H).
^{13}C-NMR (151 MHz, CDCl3) δ 154.06, 145.15, 132.57, 132.35, 127.20, 124.27, 122.46, 122.41, 121.04, 120.65, 120.00, 119.98, 103.28, 53.39, 25.94.
LCMS: Expected 266.27, found $[M+H]^+$ 267.10

4-(piperidin-1-yl)quinoline

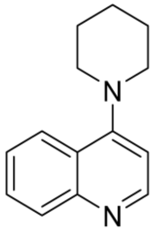

Literature (*50*).

^{1}H-NMR (600 MHz, CDCl3) δ 8.67 (d, J = 5.0 Hz, 1H), 8.02 (d, J = 8.3 Hz, 1H), 7.98 (dd, J = 8.4, 0.8 Hz, 1H), 7.61 (s, 1H), 7.44 (s, 1H), 6.77 (d, J = 5.0 Hz, 1H), 3.14 (br m, 2H), 1.81 (dt, J = 11.1, 5.7 Hz, 2H), 1.70 – 1.62 (m, 1H).
^{13}C-NMR (151 MHz, CDCl3) δ 158.06, 150.83, 149.53, 129.82, 128.94, 125.06, 123.98, 123.77, 108.64, 53.63, 26.13, 24.49.
LCMS: Expected 212.30, found $[M+H]^+$ 213.20

4-(piperidin-1-yl) -7-(trifluoromethyl)quinoline

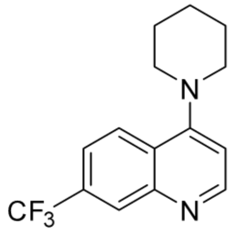

Literature (*51*).

^{1}H-NMR (600 MHz, CDCl3) δ 8.70 (d, J = 5.0 Hz, 1H), 8.29 (s, 1H), 8.05 (d, J = 8.8 Hz, 1H), 7.58 (dd, J = 8.8, 1.6 Hz, 1H), 6.82 (d, J = 5.0 Hz, 1H), 3.29 – 2.91 (m, 4H), 1.92 – 1.70 (m, 4H), 1.65 (dt, J = 11.5, 6.0 Hz, 2H).
^{13}C-NMR (151 MHz, CDCl3) δ 157.87, 152.16, 148.68, 130.53 (m), 127.61 (m), 126.82, 125.47, 125.44, 125.01, 123.21, 121.40, 120.49 (m), 110.04, 53.59, 26.00, 24.33.
LCMS: Expected 280.29, found $[M+H]^+$ 281.20

Quinolinium formation general procedure

The 4-*N*-substituted quinoline (1.0 eq) was dissolved in EtOH and 5-bromopent-1-ene (3.0 eq) was added. The mixture was stirred for 72h at 100 °C. The solvent was removed under reduced pressure and the crude product was purified by flash column chromatography on silica gel (EtOAc 0% to 100% in Cy) obtaining the final product (>95% pure by HPLC).

1-(pent-4-en-1-yl)-4-((2-phenoxyethyl)amino)quinolinium bromide

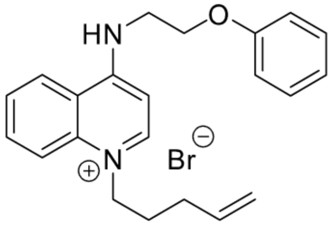

LCMS: expected 333.20, found $[M+H]^+$ 333.20

4-(benzyl(methyl)amino)-1-(pent-4-en-1-yl)quinolinium bromide

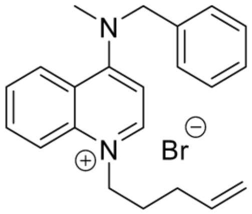

^{1}H-NMR (600 MHz, CDCl3) δ 9.43 – 9.37 (m, 1H), 8.19 – 8.15 (m, 1H), 7.96 – 7.91 (m, 2H), 7.54 (ddd, J = 8.3, 6.3, 1.8 Hz, 1H), 7.43 – 7.39 (m, 2H), 7.37 – 7.33 (m, 1H), 7.28 (t, J = 5.4 Hz, 2H), 7.25 – 7.23 (m, 1H), 5.79 (ddt, J = 16.9, 10.2, 6.6 Hz, 1H), 5.08 – 5.04 (m, 1H), 5.04 – 5.01 (m, 1H), 5.00 (s, 2H), 4.82 – 4.77 (m, 2H), 3.38 (d, J = 4.0 Hz, 3H), 2.24 (q, J = 7.2 Hz, 2H), 2.10 – 2.03 (m, 2H).
^{13}C-NMR (151 MHz, CDCl3) δ 160.77, 147.06, 139.17, 136.38, 134.23, 134.16, 129.56, 129.53, 128.56, 127.31, 126.83, 126.22, 119.84, 117.97, 116.61, 105.21, 60.27, 54.47, 42.31, 30.42, 28.38.
LCMS: Expected 317.20, found $[M+H]^+$ 318.10

4-(methyl(phenethyl)amino)-1-(pent-4-en-1-yl)quinolin-1-ium

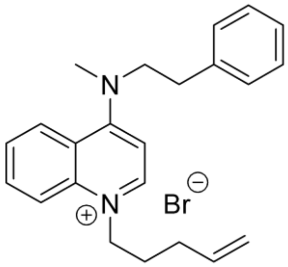

^{1}H-NMR (400 MHz, CDCl3) δ 9.16 (d, J = 7.5 Hz, 1H), 8.07 (dd, J = 8.6, 1.0 Hz, 1H), 7.89 (m, 2H), 7.58 (ddd, J = 8.3, 6.7, 1.3 Hz, 1H), 7.24 – 7.10 (m, 6H), 5.78 (ddt, J = 16.9, 10.2, 6.6 Hz, 1H), 5.10 – 4.99 (m, 2H, terminal alkene

overlapping), 4.81 – 4.49 (m, 2H), 4.05 (t, J = 7.2 Hz, 2H), 3.42 (s, 3H), 3.10 (t, J = 7.2 Hz, 2H), 2.34 – 2.11 (m, 4H), 2.11 – 1.94 (m, 2H).
LCMS: Expected 331.22, found $[M+H]^+$ 332.30

1-(pent-4-en-1-yl)-4-(phenethylamino)quinolin-1-ium

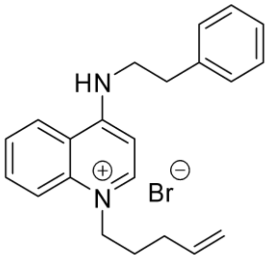

4-(dimethylamino)-1-(pent-4-en-1-yl)-7-(trifluoromethyl)quinolin-1-ium.

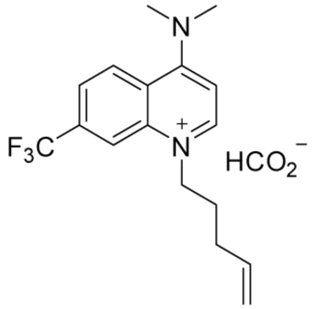

Literature (*52*) .

^{1}H-NMR (600 MHz, CDCl3) δ 9.21 (d, J = 6 Hz, 1H), 8.57 (d, J = 12 Hz, 1H), 8.45 (HCOOH) (s, 1H), 7.98 (s, 1H), 7.83 (d, J = 6 Hz, 1H), 7.17 (d, J = 6 Hz, 1H), 5.77 (ddt, J = 18 Hz, 12, 6 Hz, 1H), 5.09-4.04 (m, 2H), 4.68 (t, J =18 Hz, 6 Hz, 2H), 3.57 (s, 6H), 2.20 (q, J= 6 Hz, 2H), 2.01 (q, J = 6 Hz, 2H).
^{13}C-NMR (600 MHz, CDCl3) δ 166.32 (HCOOH), 159.50, 147.15, 138.80, 135.98, 134.92 (m), 130.32, 123.66, 121.85, 121.52, 121.98, 117.14, 114.53, 104.98, 53.98, 45.19, 30.19, 28.11.
LCMS: Expected 309.16, found $[M+H]^+$ 310.10.

4-(dimethylamino)-1-(pent-4-en-1-yl)quinolin-1-ium.

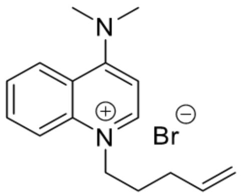

4-morpholino-1-(pent-4-en-1-yl)-7-(trifluoromethyl)quinolinium bromide

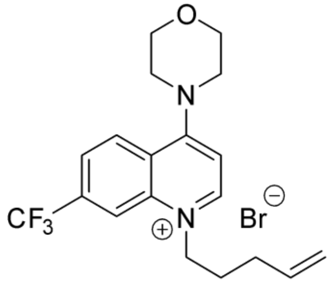

^{1}H-NMR (600 MHz, CDCl3) δ 8.77 (d, J = 5.4 Hz, 1H), 8.44 (s, 1H), 8.11 (d, J = 8.8 Hz, 1H), 7.66 (dd, J = 8.8, 1.5 Hz, 1H), 7.02 (d, J = 5.5 Hz, 1H), 5.71 (ddt, J = 16.9, 10.2, 6.6 Hz, 1H), 5.04 (dd, J = 17.1, 1.5 Hz, 1H), 5.01 (dd, J = 10.2, 1.3 Hz, 1H), 4.02 – 3.93 (m, 5H), 3.43 – 3.36 (m, 5H), 2.99 (dt, J = 25.8, 11.1 Hz, 2H), 2.15 – 2.08 (m, 2H), 2.07 – 1.96 (m, 2H).

^{13}C-NMR (151 MHz, CDCl3) δ 157.97, 149.53, 145.81, 135.74, 132.22 (m), 125.58, 125.34, 125.31, 124.44, 123.93, 122.63, 121.57, 121.55, 116.93, 109.38, 66.63, 63.52, 57.42, 52.60, 51.93, 30.56, 22.16.

LCMS: Expected 351.17, found $[M+H]^+$ 352.00

4-morpholino-1-(pent-4-en-1-yl)quinolinium bromide

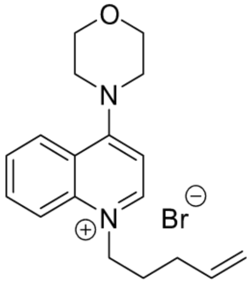

LCMS: Expected 283.18, found [M] 283.30

1-(pent-4-en-1-yl)-4-(pyrrolidin-1-yl)quinolinium bromide

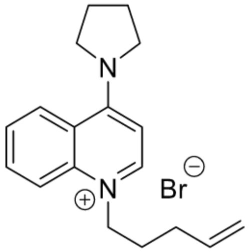

^{1}H-NMR (600 MHz, CDCl3) δ 8.96 (d, J = 7.6 Hz, 1H), 8.46 (d, J = 8.2 Hz, 1H), 7.90 – 7.85 (m, 1H), 7.81 (d, J = 8.6 Hz, 1H), 7.60 (dd, J = 11.5, 4.1 Hz, 1H), 6.89 (d, J = 7.6 Hz, 1H), 5.74 (ddt, J = 16.9, 10.2, 6.6 Hz, 1H), 5.01 (dd, J = 17.1, 1.4 Hz, 1H), 4.97 (d, J = 10.2 Hz, 1H), 4.76 – 4.48 (m, 2H), 2.17 (dd, J = 14.2, 7.1 Hz, 2H), 2.12 (s, 4H), 2.06 – 1.86 (m, 2H).

^{13}C-NMR (151 MHz, CDCl3) δ 155.60, 145.59, 138.86, 136.36, 133.63, 127.90, 125.53, 119.25, 117.18, 116.44, 102.90, 53.95, 30.33, 27.95.

LCMS: Expected 267.19, found $[M+H]^+$ 268.20

1-(pent-4-en-1-yl)-4-(pyrrolidin-1-yl)-7-(trifluoromethyl)quinolinium bromide

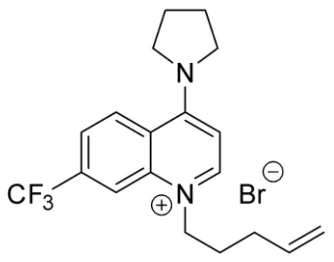

LCMS: Expected 335.17, found $[M+H]^+$ 336.20

1-(pent-4-en-1-yl)-4-(piperidin-1-yl)quinolinium bromide

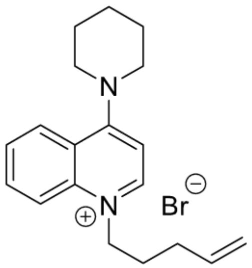

^{1}H-NMR (600 MHz, CDCl3) δ 9.31 (d, J = 7.3 Hz, 1H), 8.00 (d, J = 8.4 Hz, 1H), 7.94 – 7.86 (m, 2H), 7.65 – 7.56 (m, 1H), 7.20 (d, J = 7.3 Hz, 1H), 5.72 (ddt, J = 16.9, 10.2, 6.6 Hz, 1H), 4.99 (dt, J = 12.2, 6.1 Hz, 1H), 4.94 (dd, J = 10.2, 1.2 Hz, 1H), 4.75 – 4.65 (m, 2H), 3.77 – 3.64 (m, 4H), 2.18 (q, J = 7.1 Hz, 2H), 2.06 – 1.94 (m, 2H), 1.86 – 1.69 (m, 6H).
^{13}C-NMR (151 MHz, CDCl3) δ 161.01, 147.05, 139.06, 136.26, 134.18, 127.36, 126.25, 120.43, 117.90, 116.38, 106.36, 54.27, 53.54, 30.27, 28.29, 25.82, 23.65.
LCMS: Expected 281.20, found $[M+H]^+$ 282.20

1-(pent-4-en-1-yl)-4-(piperidin-1-yl)-7-(trifluoromethyl)quinolinium bromide

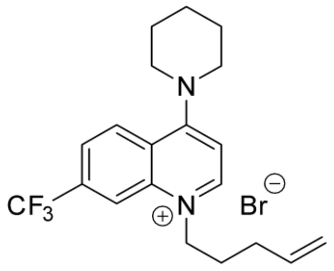

^{1}H-NMR (600 MHz, CDCl3) δ 9.84 (d, J = 7.4 Hz, 1H), 8.19 (t, J = 12.1 Hz, 1H), 8.08 (s, 1H), 7.84 (t, J = 10.3 Hz, 1H), 7.49 (t, J = 27.2 Hz, 1H), 5.84 (ddt, J = 17.0, 10.2, 6.7 Hz, 1H), 5.21 – 5.00 (m, 2H, terminal alkene overlapping), 4.95 – 4.74 (m, 2H), 3.97 – 3.70 (m, 4H), 2.39 – 2.18 (m, 2H), 2.21 – 1.92 (m, 3H), 2.00 – 1.76 (m, 6H).
^{13}C-NMR (151 MHz, CDCl3) δ 160.08, 148.17, 138.79, 135.97, 135.05, 134.83, 129.57, 125.36, 123.54, 121.97, 121.85, 121.73, 119.92, 116.88, 114.89, 107.24, 53.98, 53.77, 30.09, 28.21, 25.91, 23.45.
LCMS: expected 349.19, found $[M+H]^+$ 350.20

1-ethyl-4-morpholino-7-(trifluoromethyl)quinolin-1-ium bromide

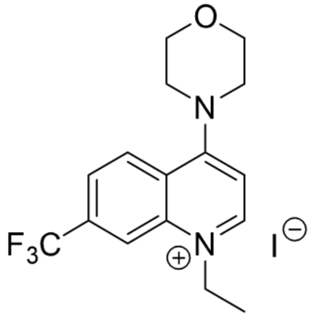

Prepared as in the general method. A sample was crystallized from tBuOMe with a few drops of ethanol. Found to be the tri-iodide salt on crystallography.

^{1}H-NMR (500 MHz, CDCl3) δ 9.32 (d, J = 10 Hz, 1H), 8.39 (d, J= 10 Hz, 1H), 8.12 (s, 1H), 7.90 (d, J = 10 Hz, 1H), 7.55 (d, J = 10 Hz, 1H), 4.86 (q, J = 10, 2H), 3.98 (s, 8H), 1.67 (t, J = 10.2 Hz, 3H).
^{13}C-NMR (500 MHz, CDCl3) δ 160.43, 147.87, 138.95, 135.54 (m), 129.67, 122.81, 122.29, 115.36, 108.56, 66.61, 53.17, 51.16, 34.72, 15.32.
HRMS (ESI-TOF) m/z: $[M+H]^+$ calcd for $C_{16}H_{19}ON_2F_3$ 312.1366, found 312.1388

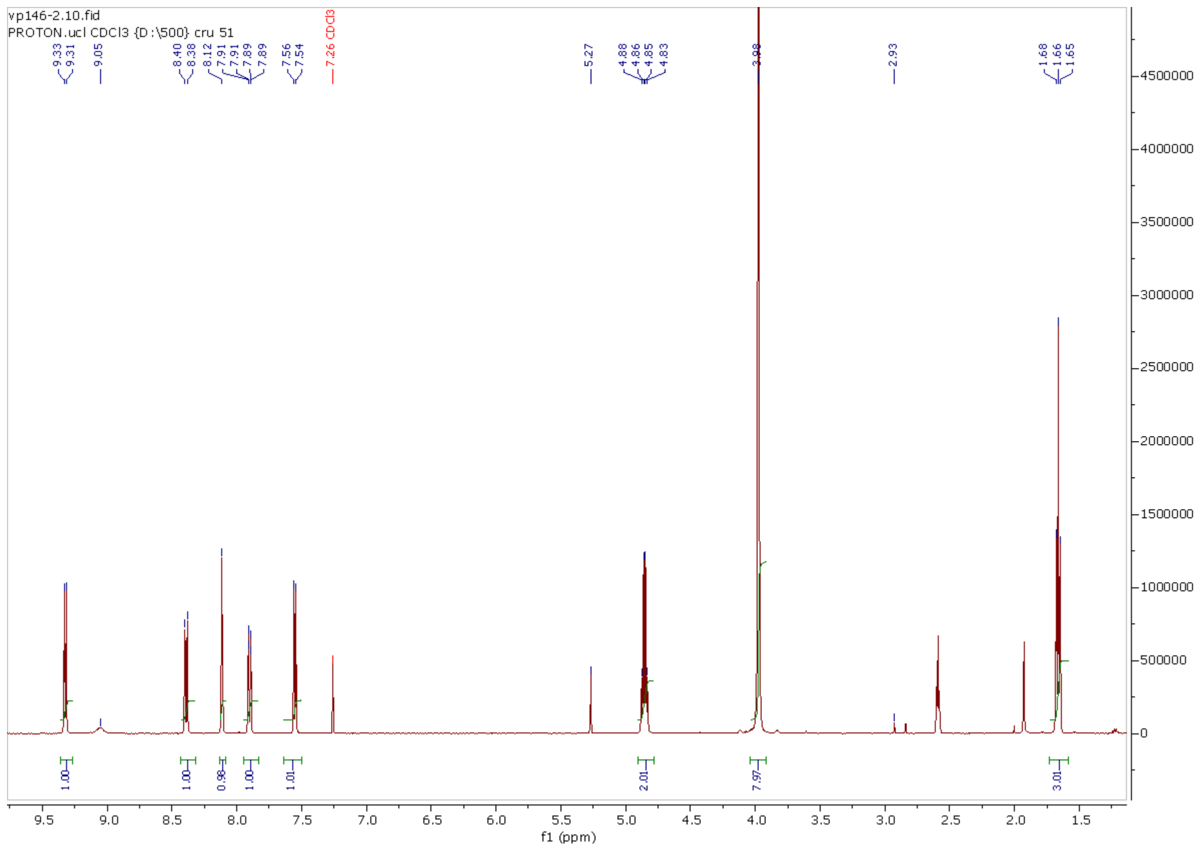

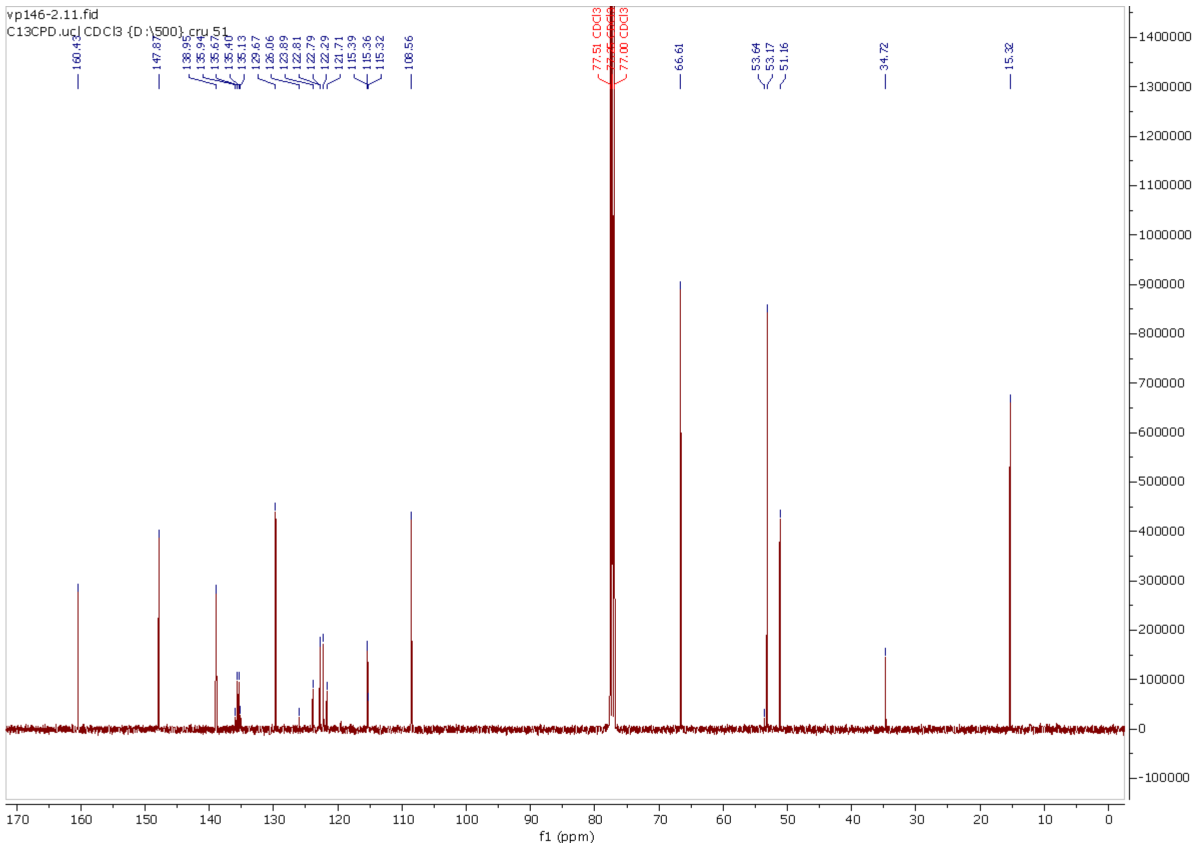

1-ethylquinolin-1-ium iodide DS292

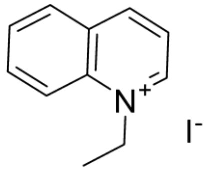

Prepared as in the general method, a sample was crystallized from ethanol.[12]

^{1}H NMR (600 MHz, MeOD) δ 9.50 (dd, *J* = 5.8, 1.5 Hz, 1H), 9.23 (dt, *J* = 8.3, 1.2 Hz, 1H), 8.62 (dd, *J* = 8.9, 1.1 Hz, 1H), 8.45 (dd, *J* = 8.3, 1.4 Hz, 1H), 8.30 (ddd, *J* = 8.8, 7.0, 1.5 Hz, 1H), 8.12 (dd, *J* = 8.4, 5.8 Hz, 1H), 8.06 (ddd, *J* = 8.0, 7.0, 0.9 Hz, 1H), 5.18 (q, *J* = 7.3 Hz, 2H), 1.75 (t, *J* = 7.3 Hz, 3H).
^{13}C NMR (151 MHz, MeOD) δ 150.08, 148.87, 139.25, 137.25, 132.13, 131.71, 131.30, 123.25, 119.72, 54.88, 15.71.

Conjugation of <u>quinolinium cations to cyclosporine by olefin cross-metathesis</u>

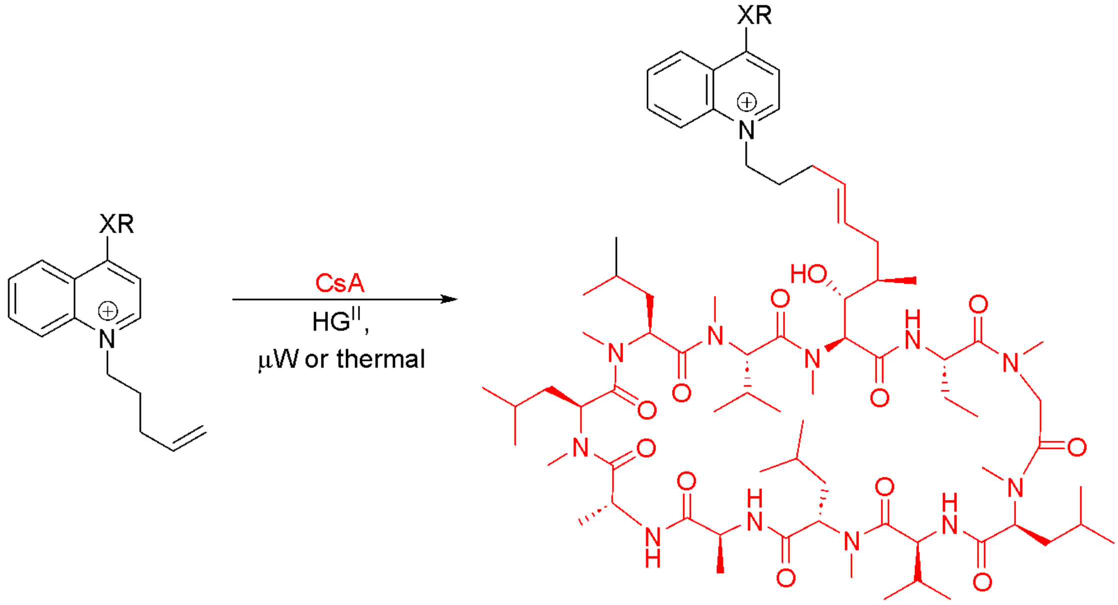

Figure S6. Conjugation of **quinolinium cations to cyclosporine by olefin cross-metathesis.**

Metathesis general procedure

Hoveyda-Grubbs 2nd generation catalyst (17 mol%) was added to a solution of quinolinium (1.2 eq) and CsA (1 eq) in DCM or DCE (3 mL) and heated in a microwave at 90 °C for 90min. After confirming the formation of the desired product by LCMS, the reaction was cooled to RT and stirred with $P(CH_2OH)_3$ overnight. The reaction was washed with water, and the organic layer dried over $MgSO_4$, filtered and concentrated *in vacuo*. The residue was purified by flash column chromatography on silica gel, eluting with 5% MeOH in DCM followed by C18 reverse phase column chromatography, eluting with 0-100% MeCN in H_2O + 0.1% formic acid.

Metathesis procedure for larger scale preparations

Hoveyda-Grubbs 2nd generation catalyst (10 mol%) was added to a solution of quinolinium (3 eq), CsA (1 eq) and 2,6-dichloro-1,4-benzoquinone (0.5 eq) in DCE (1.5 mL) and LiCl/DMF (0.4M, 0.15 mL). The reaction mixture was heated in a microwave at 70 °C for 16h. After confirming the formation of the desired product by LCMS, the reaction was cooled to RT, the solvent was removed under reduced pressure, the crude product was redissolved in MeOH and passed through a Stratospheres PL Thiol MP SPE cartridge (polymer Lab, Varian Inc) to remove the catalyst. The crude product was purified by C4 reverse phase column chromatography, eluting with H_2O 85%, MeOH 15% + 0.1% formic acid and MeCN 85%, MeOH 15% + 0.1% formic acid.

TWH32

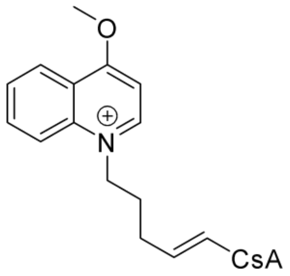

4-Methoxyquinoline-1-(pent-4-en-1-yl) quinolinium bromide (31 mg, 0.10 mmol) and CsA (100 mg, 0.082 mmol) in DCM (3 mL) was treated with Hoveyda-Grubbs 2nd generation catalyst (9 mg, 0.014 mmol) using the general procedure above to give TWH32 (32 mg, 28%) as a white solid.

^{1}H-NMR (600 MHz, $CDCl_3$) 3.46 (3H, s, NMe), 3.37 (3H, s, NMe), 3.22 (3H, s, NMe), 3.10 (3H, s, NMe), 3.08 (3H, s, NMe), 2.69 (3H, s, NMe), 2.66 (3H, s, NMe).

^{13}C-NMR (150 MHz, $CDCl_3$) 39.42, 39.08, 33.87, 31.59, 31.22, 29.93, 29.80 (7 x NMe)

LCMS: $[M+H]^+$ Calcd for $C_{75}H_{124}N_{12}O_{13}$ 1388.93; found 1388.90

TWH30

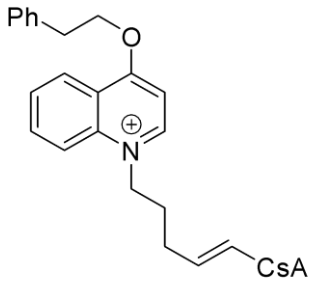

4-Phenethoxyquinoline-1-(pent-4-en-1-yl)quinolinium bromide (40 mg, 0.10 mmol) and CsA (100 mg, 0.082 mmol) in DCM (3 mL) was treated with Hoveyda-Grubbs 2nd generation catalyst (9 mg, 0.014 mmol) using the general procedure above to give TWH30 (19 mg, 16%) as a white solid.

^{1}H-NMR (600 MHz, $CDCl_3$) 3.48 (3H, s, NMe), 3.38 (3H, s, NMe), 3.22 (3H, s, NMe), 3.19 (3H, s, NMe), 3.12 (3H, s, NMe), 2.69 (3H, s, NMe), 2.67 (3H, s, NMe).

^{13}C-NMR (150 MHz, $CDCl_3$) 39.52, 39.10, 33.79, 31.33, 30.04, 29.96, 29.71 (7 x NMe).

LCMS: $[M+H]^+$ Calcd for $C_{81}H_{130}N_{12}O_{13}$ 1478.98; found 1480.00

TWH36

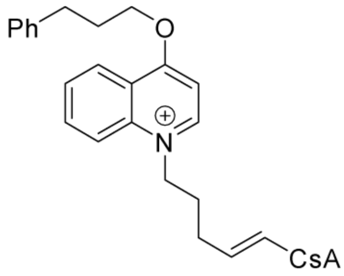

4-(3-phenylpropoxy)-1-(pent-4-en-1-yl)quinolinium bromide (41 mg, 0.10 mmol) and CsA (100 mg, 0.082 mmol) in DCM (3 mL) was treated with Hoveyda-Grubbs 2nd generation catalyst (9 mg, 0.014 mmol) using the general procedure above to give TWH36 (18 mg, 15%) as a white solid.
^{1}H-NMR (600 MHz, $CDCl_3$) 3.48 (3H, s, NMe), 3.37 (3H, s, NMe), 3.19 (3H, s, NMe), 3.12 (3H, s, NMe), 3.11 (3H, s, NMe), 2.70 (3H, s, NMe), 2.68 (3H, s, NMe).
^{13}C-NMR (150 MHz, $CDCl_3$) 39.45, 39.11, 32.30, 31.33, 30.08, 29.97, 29.74 (7 x NMe).
LCMS: $[M+H]^+$ Calcd for $C_{82}H_{133}N_{12}O_{13}$ 1492.99; found 1493.00

TWH44

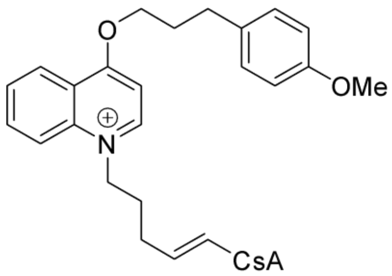

4-(3-(4-methoxyphenyl)propoxy)-1-(pent-4-en-1-yl)quinolinium bromide (44 mg, 0.10 mmol) and CsA (100 mg, 0.082 mmol) in DCM (3 mL) was treated with Hoveyda-Grubbs 2nd generation catalyst (9 mg, 0.014 mmol) using the general procedure above to give TWH44 (14 mg, 11%) as a white solid.
^{1}H-NMR (600 MHz, $CDCl_3$) 3.48 (3H, s, NMe), 3.37 (3H, s, NMe), 3.23 (3H, s, NMe), 3.12 (3H, s, NMe), 3.11 (3H, s, NMe), 2.70 (3H, s, NMe), 2.67 (3H, s, NMe).
^{13}C-NMR (150 MHz, $CDCl_3$) 39.39, 39.12, 33.74, 31.35, 30.29, 29.97, 29.76 (7 x NMe).
LCMS: $[M+H]^+$ Calcd for $C_{83}H_{134}N_{12}O_{14}$ 1523.01; found 1523.00

TWH53

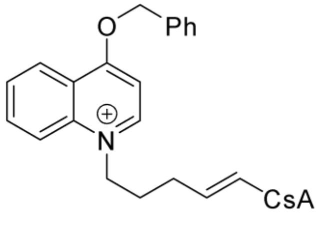

4-benzyloxy-1-(pent-4-en-1-yl)quinolinium bromide (38 mg, 0.10 mmol) and CsA (100 mg, 0.082 mmol) in DCM (3 mL) was treated with Hoveyda-Grubbs 2nd generation catalyst (9 mg, 0.014 mmol) using the general procedure above to give TWH53 (35 mg, 29%) as a white solid.
^{1}H-NMR (600 MHz, $CDCl_3$) 3.48 (3H, s, NMe), 3.38 (3H, s, NMe), 3.24 (3H, s, NMe), 3.11 (3H, s, NMe), 3.10 (3H, s, NMe), 2.70 (3H, s, NMe), 2.67 (3H, s, NMe).
^{13}C-NMR (150 MHz, $CDCl_3$) 39.52, 33.96, 31.46, 31.25, 30.00, 29.82, 29.72 (7 x NMe).
LCMS: $[M+H]^+$ Calcd for $C_{80}H_{128}N_{12}O_{13}$ 1464.96; found 1465.00

TWH68

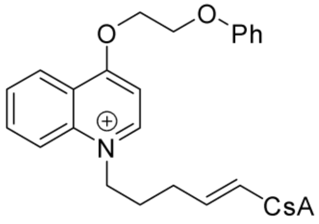

4-(2-phenoxyethoxy)-1-(pent-4-en-1-yl)quinolinium bromide (41 mg, 0.10 mmol) and CsA (100 mg, 0.082 mmol) in DCM (3 mL) was treated with Hoveyda-Grubbs 2nd generation catalyst (9 mg, 0.014 mmol) using the general procedure above to give TWH68 (10 mg, 8%) as a white solid.
^{1}H-NMR (600 MHz, $CDCl_3$) 3.48 (3H, s, NMe), 3.38 (3H, s, NMe), 3.19 (3H, s, NMe), 3.12 (3H, s, NMe), 3.11 (3H, s, NMe), 2.69 (3H, s, NMe), 2.67 (3H, s, NMe).
^{13}C-NMR (150 MHz, $CDCl_3$) 39.50, 33.75, 31.51, 31.34, 30.06, 29.83, 29.72 (7 x NMe).
LCMS: $[M+H]^+$ Calcd for $C_{81}H_{130}N_{12}O_{14}$ 1494.98; found 1494.90

TWH46

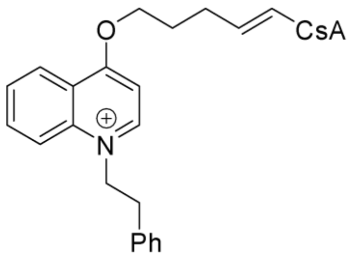

4-(pent-4-en-1-yloxy)-1-(phenethoxy)quinolinium bromide (40 mg, 0.10 mmol) and CsA (100 mg, 0.082 mmol) in DCM (3 mL) was treated with Hoveyda-Grubbs 2nd generation catalyst (9 mg, 0.014 mmol) using the general procedure above to give TWH46 (9 mg, 8%) as a white solid.
^{1}H-NMR (600 MHz, $CDCl_3$) 3.48 (3H, s, NMe), 3.37 (3H, s, NMe), 3.23 (3H, s, NMe), 3.11 (3H, s, NMe), 3.10 (3H, s, NMe), 2.71 (3H, s, NMe), 2.68 (3H, s, NMe).
^{13}C-NMR (150 MHz, $CDCl_3$) 39.38, 39.11, 33.92, 31.24, 30.10, 30.02, 29.97 (7 x NMe).
LCMS: $[M+H]^+$ Calcd for $C_{81}H_{130}N_{12}O_{13}$ 1478.98; found 1480.00 [MH]+.

TWH43

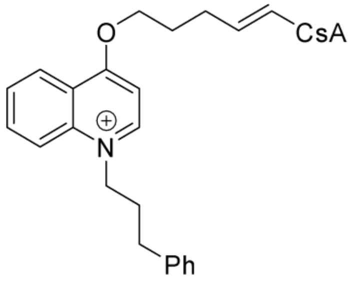

4-(pent-4-en-1-yloxy)-1-(3-phenylpropyl)quinolinium bromide (41 mg, 0.10 mmol) and CsA (100 mg, 0.082 mmol) in DCM (3 mL) was treated with Hoveyda-Grubbs 2nd generation catalyst (9 mg, 0.014 mmol) using the general procedure above to give TWH43 (28 mg, 23%) as a white solid.
^{1}H-NMR (600 MHz, $CDCl_3$) δ 3.47 (3H, s, NMe), 3.36 (3H, s, NMe), 3.23 (3H, s, NMe), 3.11 (3H, s, NMe), 3.09 (3H, s, NMe), 2.70 (3H, s, NMe), 2.67 (3H, s, NMe).
^{13}C-NMR (150 MHz, $CDCl_3$) δ 39.08, 33.94, 31.42, 31.24, 30.00, 29.95, 29.75 (7 x NMe).
LCMS: $[M+H]^+$ Calcd for $C_{82}H_{132}N_{12}O_{13}$ 1492.99; found 1494.00 [MH]+

JP1-068

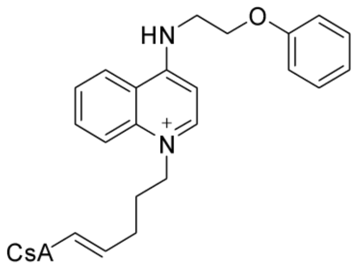

^{1}H-NMR (600 MHz, $CDCl_3$) δ 3.50 (3H, s, NMe), 3.39 (3H, s, NMe), 3.20 (3H, s, NMe), 3.12 (6H, br s, NMe), 2.69 (3H, s, NMe), 2.67 (3H, s, NMe)
^{13}C-NMR (150 MHz, $CDCl_3$) δ 39.14, 33.49, 31.40, 30.25 (2 x CH3), 29.92, 29.70 (7 x NMe).
LCMS: $[M+H]^+$ Calcd for $C_{81}H_{131}N_{13}O_{13}$ 1493.99; found 1495.00 $[MH]^+$

JP1-028

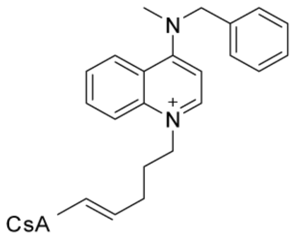

^{1}H-NMR (600 MHz, $CDCl_3$) δ 3.69 (3H, s, NMe (NMe-benzylamine)), 3.48 (3H, s, NMe), 3.37 (3H, s, NMe), 3.19 (3H, s, NMe), 3.11 (3H, s, NMe), 3.10 (3H, s, NMe), 2.70 (3H, s, NMe), 2.67 (3H, s, NMe)
^{13}C-NMR (150 MHz, $CDCl_3$) δ 39.42, 33.80, 31.42, 31.11, 30.27, 30.18, 29.76 (7 x NMe), assigned by HSQC).
LCMS: $[M+H]^+$ Calcd for $C_{81}H_{130}N_{13}O_{12}$ 1478.00; found 1478.00 $[MH]^+$

JP1-037

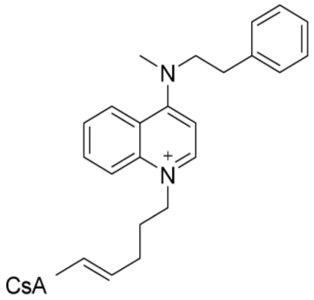

LCMS: $[M+H]^+$ Calcd for $C_{82}H_{132}N_{13}O_{12}$ 1492.01; found 1492.00 $[MH]^+$

JP1-037H

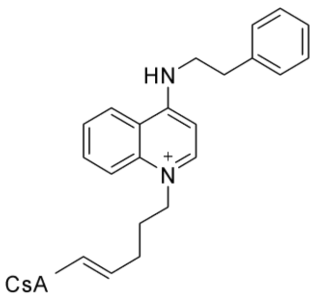

^{1}H-NMR (600 MHz, $CDCl_3$) δ 3.48 (3H, s, NMe), 3.38 (3H, s, NMe), 3.20 (3H, s, NMe), 3.13 (3H, s, NMe), 3.10 (3H, s, NMe), 2.70 (3H, s, NMe), 2.68 (3H, s, NMe).
^{13}C-NMR (151 MHz, CDCl3) δ 39.17, 37.55, 36.25, 33.66, 32.05, 31.58, 29.82 (7 x NMe).

JW76

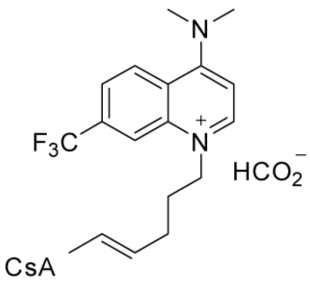

^{1}H-NMR (500 MHz, CDl3, δ ppm) δ 3.47 (s, NMe, 3H), 3.39 (s, NMe, 3H), 3.24 (s, NMe, 3H), 3.12 (s, NMe, 3H), 3.11 (s, NMe, 3H), 2.68 (s, NMe, 3H), 2.66 (s, NMe, 3H).
^{13}C-NMR (600 MHz, CDCl3) δ 173.90 (C=O), 173.77 (C=O), 173.71 (C=O), 173.67 (C=O), 173.66 (C=O), 173.63 (C=O), 173.60(C=O), 173.58 (C=O), 173.55(C=O), 173.53(C=O), 173.46 (C=O).
HRMS (*m/z*): $[MH]^+$ calcd. for $C_{76}H_{125}F_3N_{13}O_{12}$, 1469.95173; found 1469.95508

JP1-180

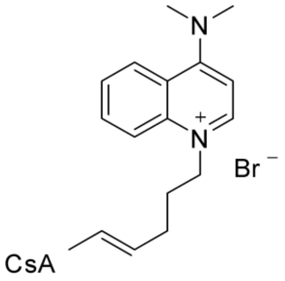

LCMS (*m/z*): $[MH]^+$ calcd. for $C_{75}H_{126}N_{13}O_{12}$, 1400.96; found 1400.97 $[MH]^+$

JP1-138

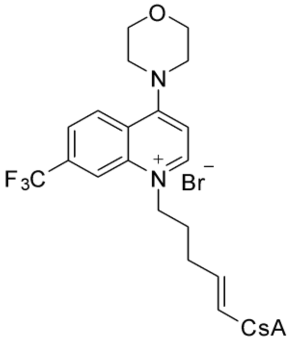

Synthesis on 50 µMole scale, yield 7.0 mg, 9.3% after purification.

^{1}H-NMR (600 MHz, $CDCl_3$) δ 3.47 (3H, s, NMe), 3.39 (3H, s, NMe), 3.23 (3H, s, NMe), 3.12 (3H, s, NMe), 3.10 (3H, s, NMe), 2.67 (3H, s , NMe), 2.66 (3H, s , NMe).
^{13}C-NMR (600 MHz, $CDCl_3$) δ 172.22 (C=O), 172.06 (C=O), 171.98 (C=O), 171.88 (C=O), 171.72 (C=O), 171.58 (C=O), 171.33 (C=O), 171.07 (C=O), 170.95 (C=O), 170.80 (C=O), 170.42 (C=O).
HRMS (*m/z*): $[MH]^+$ calcd. for $C_{78}H_{128}F_3N_{13}O_{13}$, 1510.96229; found 1510.9628.

JP1-141

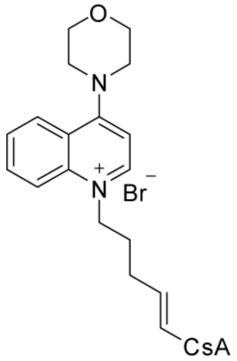

^{1}H-NMR (600 MHz, $CDCl_3$) δ 3.47 (3H, s, NMe), 3.38 (3H, s, NMe), 3.19 (3H, s, NMe), 3.11 (3H, s, NMe), 3.10 (3H, s, NMe), 2.69 (3H, s, NMe), 2.67 (3H, s, NMe)
^{13}C-NMR (150 MHz, $CDCl_3$) δ 39.52, 39.13, 37.55, 34.47, 31.31, 29.95, 29.72 (7 x NMe).

[MH]+ calcd. for $C_{77}H_{129}N_{13}O_{13}$, 1443.97; found 1443.90
HRMS (*m/z*): [MH]+ calcd. for $C_{77}H_{128}N_{13}O_{13}$, 1442.9749; found 1442.9757.

JP1-159

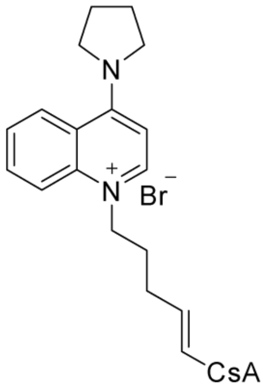

^{1}H-NMR (600 MHz, $CDCl_3$) δ 3.48 (3H, s, NMe), 3.37 (3H, s, NMe), 3.19 (3H, s, NMe), 3.12 (3H, s, NMe), 3.10 (3H, s, NMe), 2.69 (3H, s, NMe), 2.67 (3H, s, NMe)
^{13}C-NMR (150 MHz, $CDCl_3$) δ 39.44, 33.77, 31.44, 31.29, 30.03, 29.95, 29.74 (7 x NMe).
HRMS [MH]+ calcd. for $C_{77}H_{128}N_{13}O_{12}$, 1426.9800; found 1426.9800

JP1-140

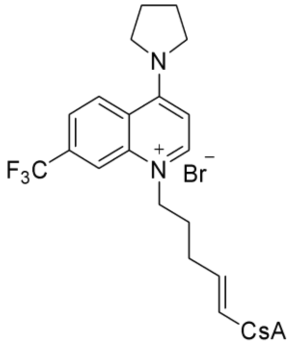

^{1}H-NMR (600 MHz, $CDCl_3$) δ 3.46 (3H, s, NMe), 3.42 (3H, s, NMe), 3.21 (3H, s, NMe), 3.12 (3H, s, NMe), 3.08 (3H, s, NMe), 2.69 (3H, s, NMe), 2.66 (3H, s, NMe)
^{13}C-NMR (150 MHz, $CDCl_3$) δ 39.39, 33.55, 31.55, 31.41, 30.09, 29.87, 29.78 (7 x NMe).[1]
HRMS [MH]+ calcd. for $C_{78}H_{128}F_3N_{13}O_{12}$, 1494.9674; found 1494.9673

[1] assigned by HSQC

JP1-166

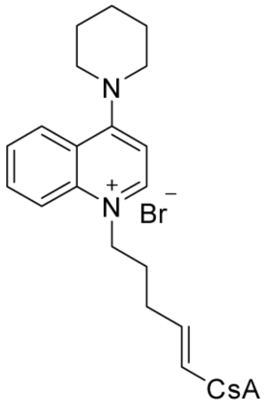

HRMS $[MH]^+$ calcd. for $C_{78}H_{130}N_{13}O_{12}$, 1440.9956; found 1441.0042

JP1-164

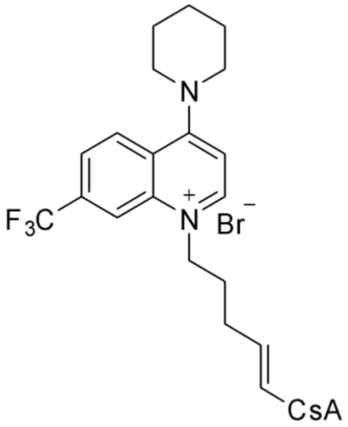

HRMS $[MH]^+$ calcd. for $C_{79}H_{129}N_{13}O_{12}F_3$, 1508.9830; found 1508.9833

^{1}H-NMR, ^{13}C-NMR and HRMS of the key compounds

JW76

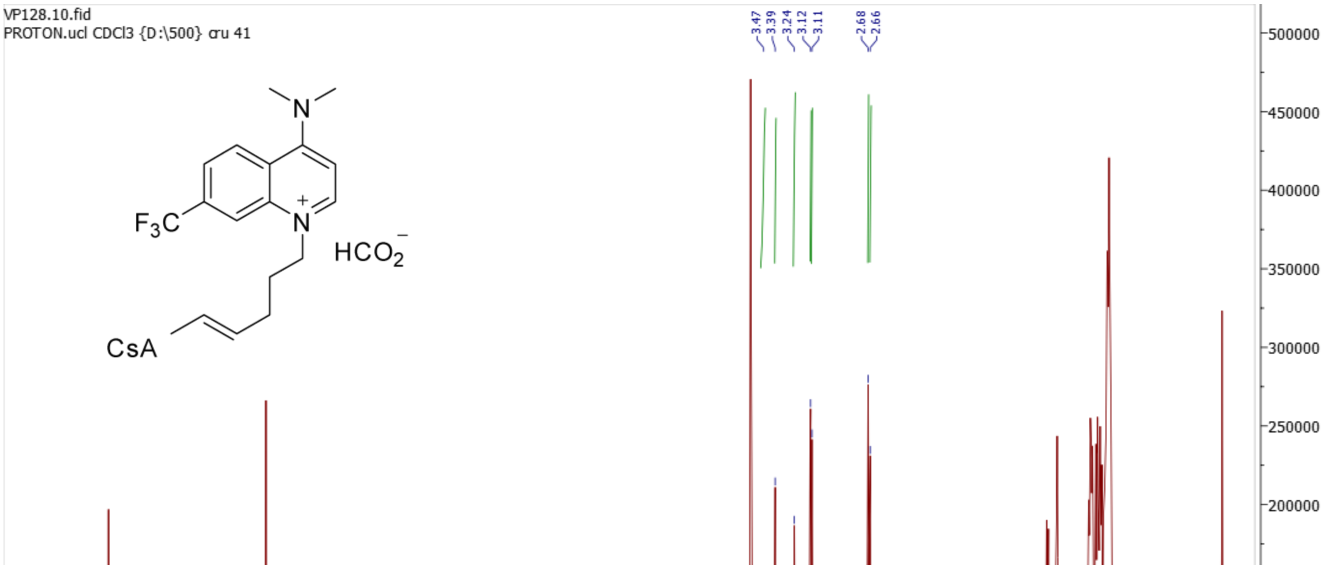

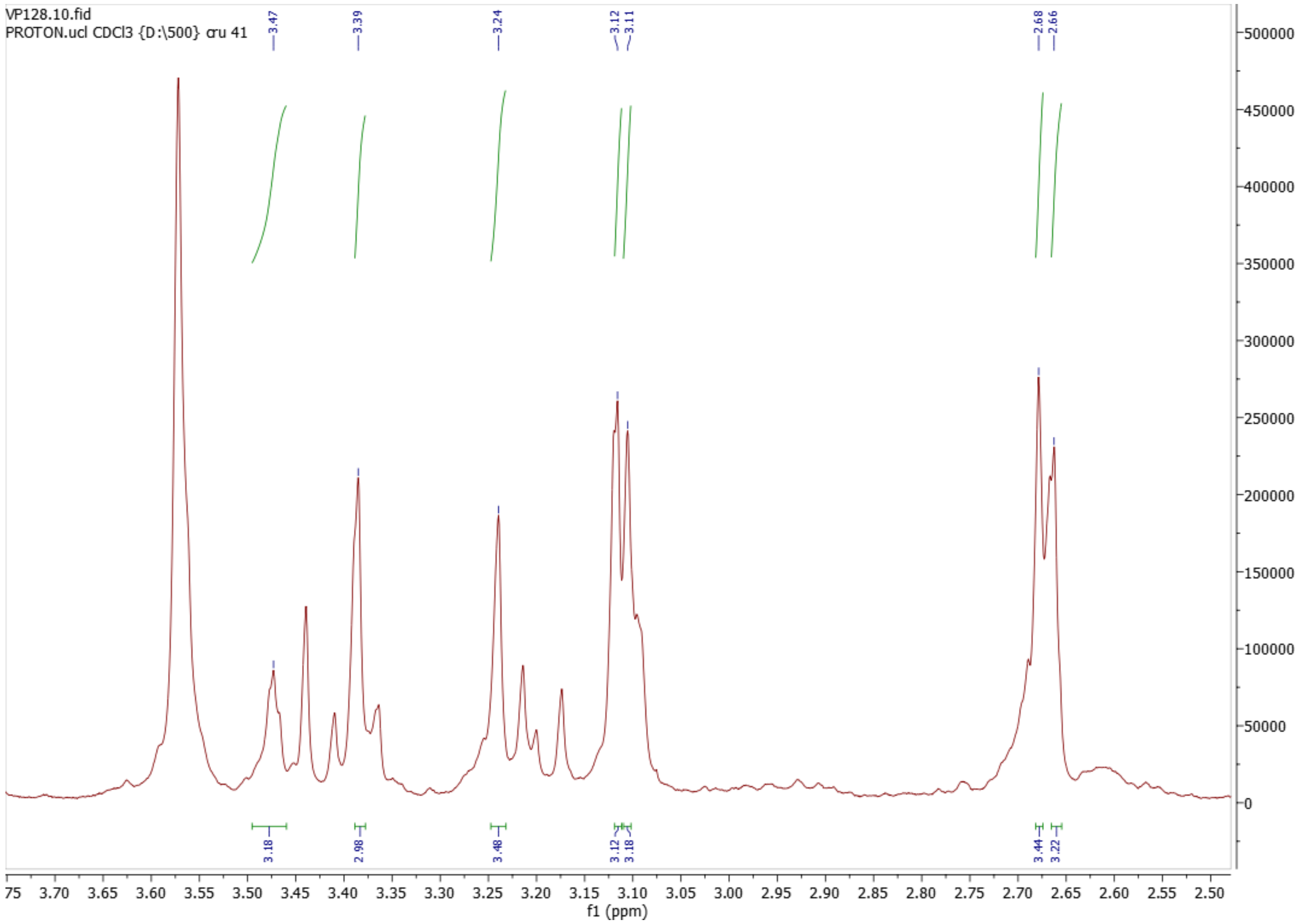

VP128.10.fid
PROTON.ucl CDCl3 {D:\500} cru 41
3.47
3.39
3.24
3.12
3.11
2.68
2.66
3.18
2.98
3.48
3.12
3.18
3.44
3.22
f1 (ppm)

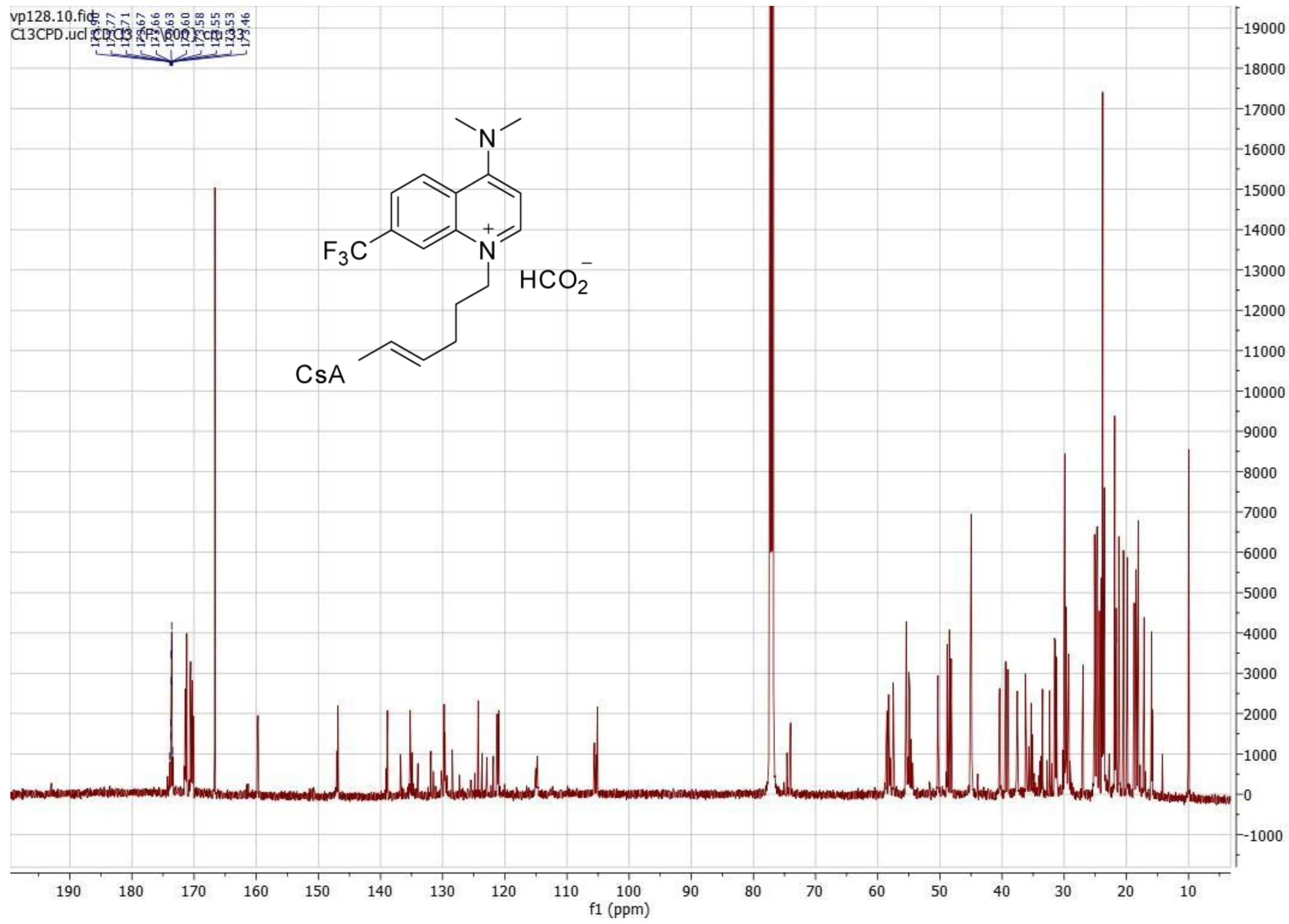

vp128.10.fid
C13CPD.ucl CDCl3
F3C
N
HCO2
CsA
f1 (ppm)

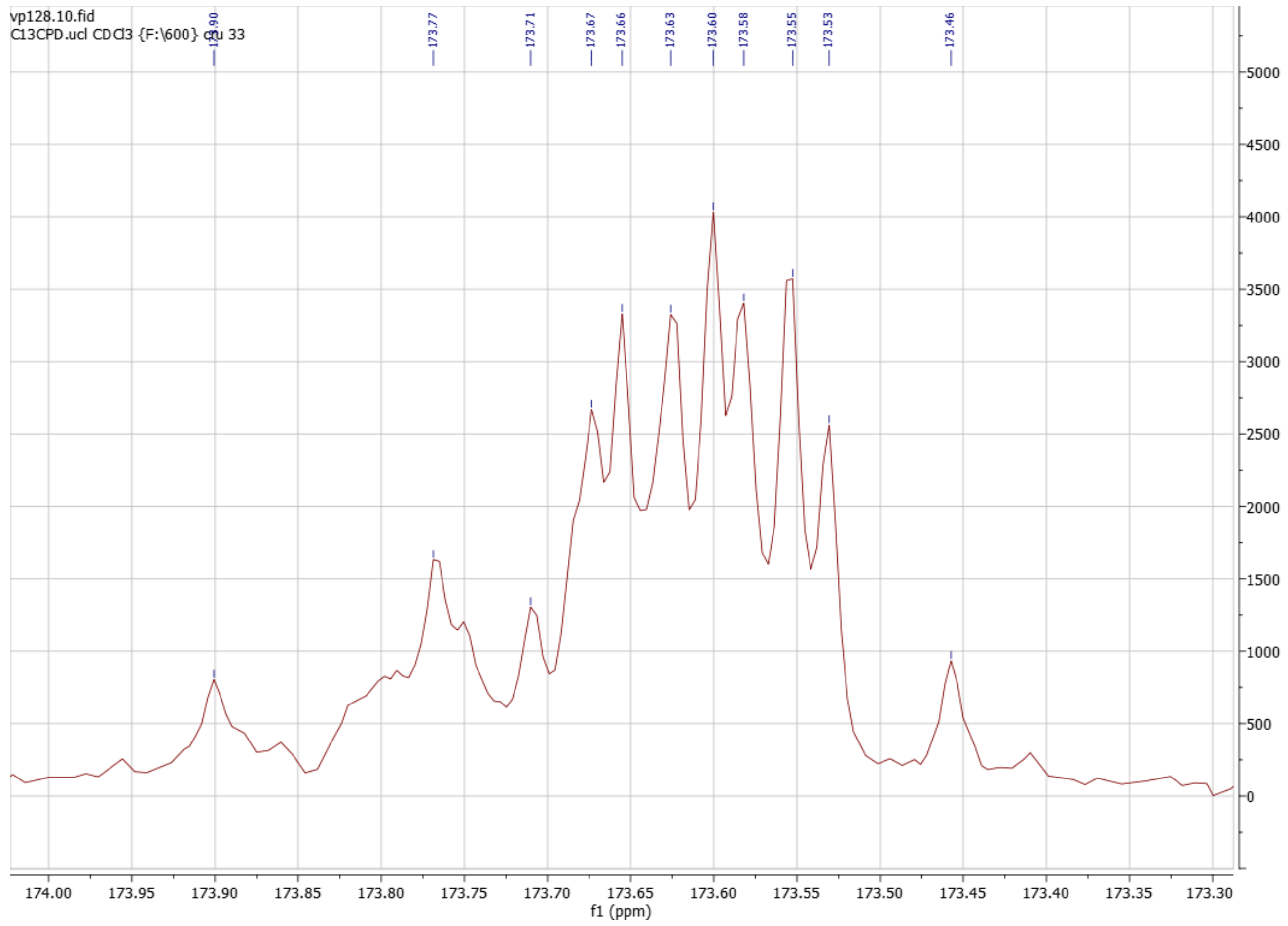

vp128.10.fid
C13CPD.ucl CDCl3 {F:\600} cru 33
173.90
173.77
173.71
173.67
173.66
173.63
173.60
173.58
173.55
173.53
173.46
f1 (ppm)

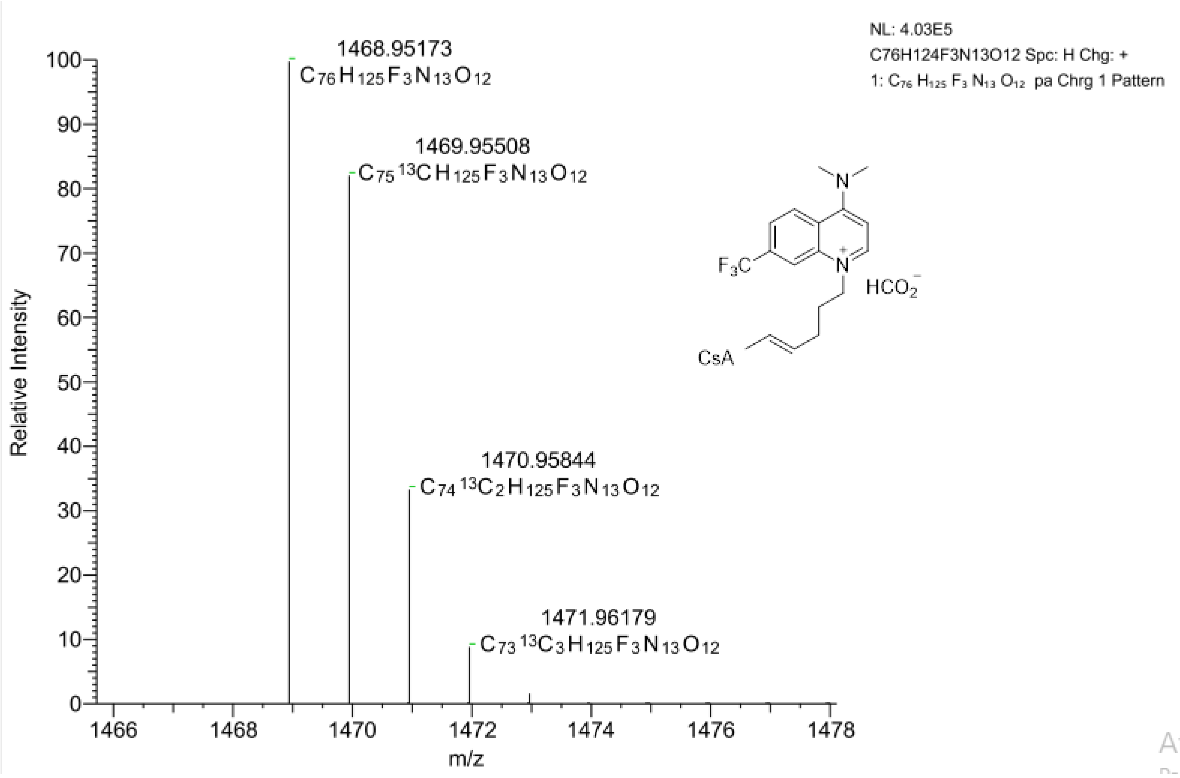

NL: 4.03E5
C76H124F3N13O12 Spc: H Chg: +
1: C76 H125 F3 N13 O12 pa Chrg 1 Pattern
1468.95173
C76 H125 F3 N13 O12
1469.95508
C75 13C H125 F3 N13 O12
1470.95844
C74 13C2 H125 F3 N13 O12
1471.96179
C73 13C3 H125 F3 N13 O12
Relative Intensity
m/z
F3C
N
HCO2
CsA

At

JP1-138

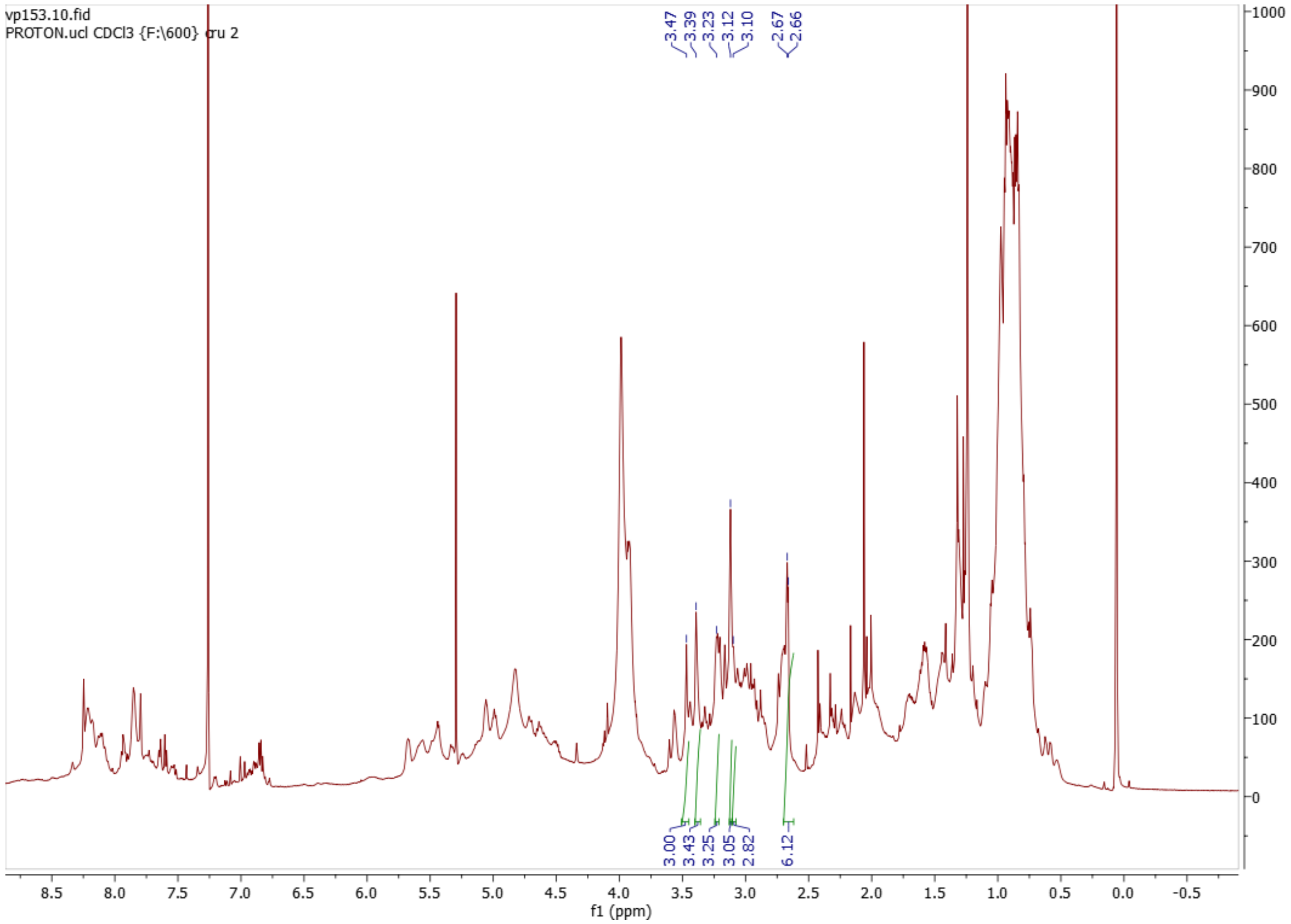

vp153.10.fid
PROTON.ucl CDCl3 {F:\600} cru 2
3.47
3.39
3.23
3.12
3.10
2.67
2.66
3.00
3.43
3.25
3.05
2.82
6.12
f1 (ppm)

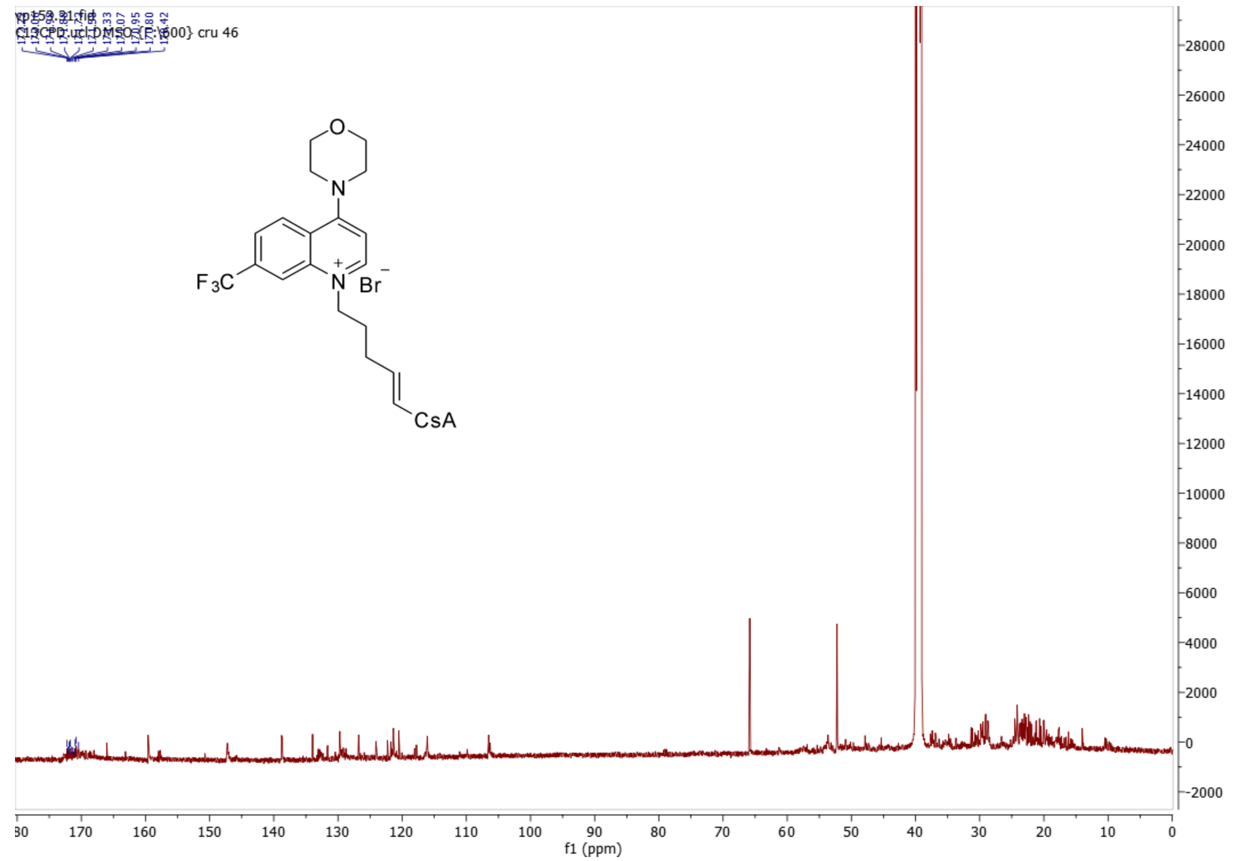

N
O
F3C
N
Br
CsA
f1 (ppm)

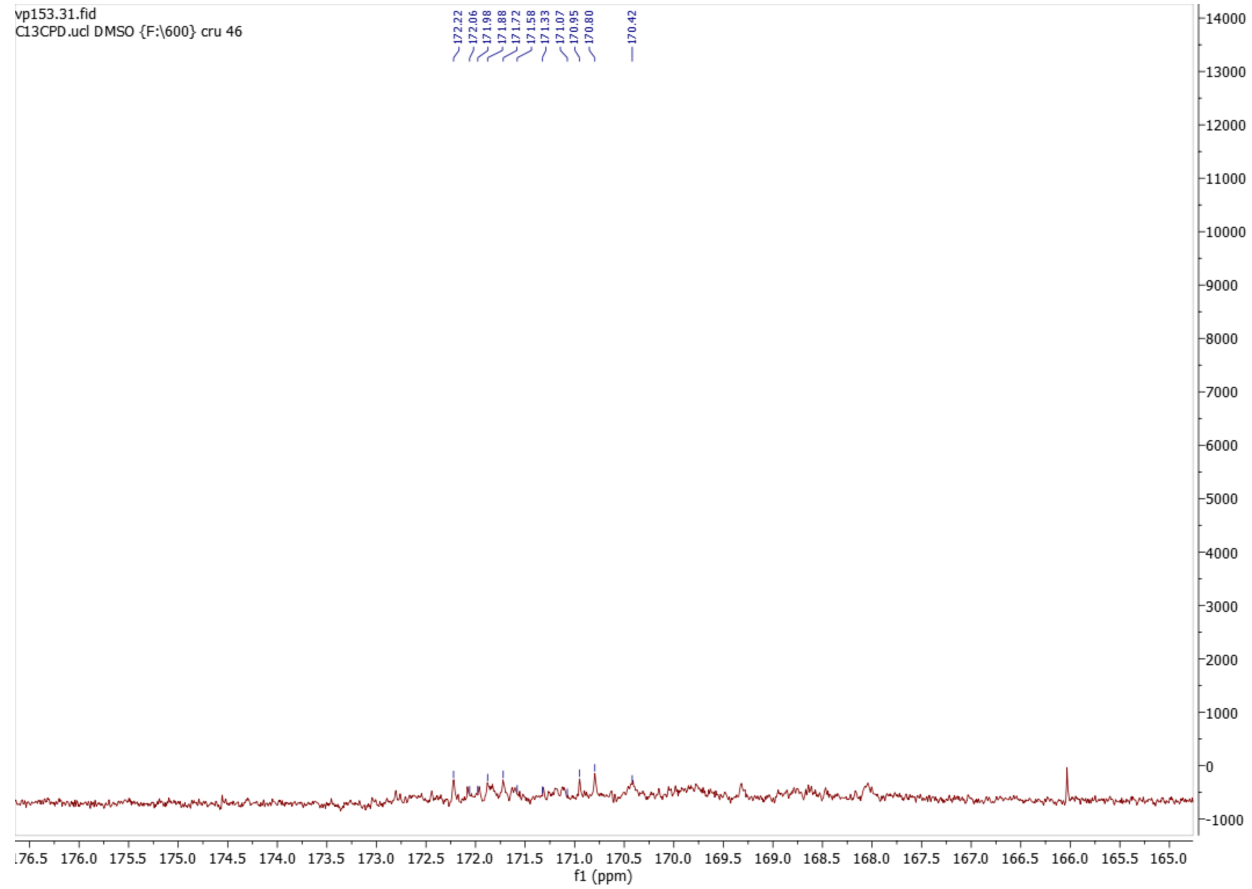

vp153.31.fid
C13CPD.ucl DMSO {F:\600} cru 46
172.22
172.06
171.98
171.88
171.72
171.58
171.33
171.07
170.95
170.80
170.42
f1 (ppm)

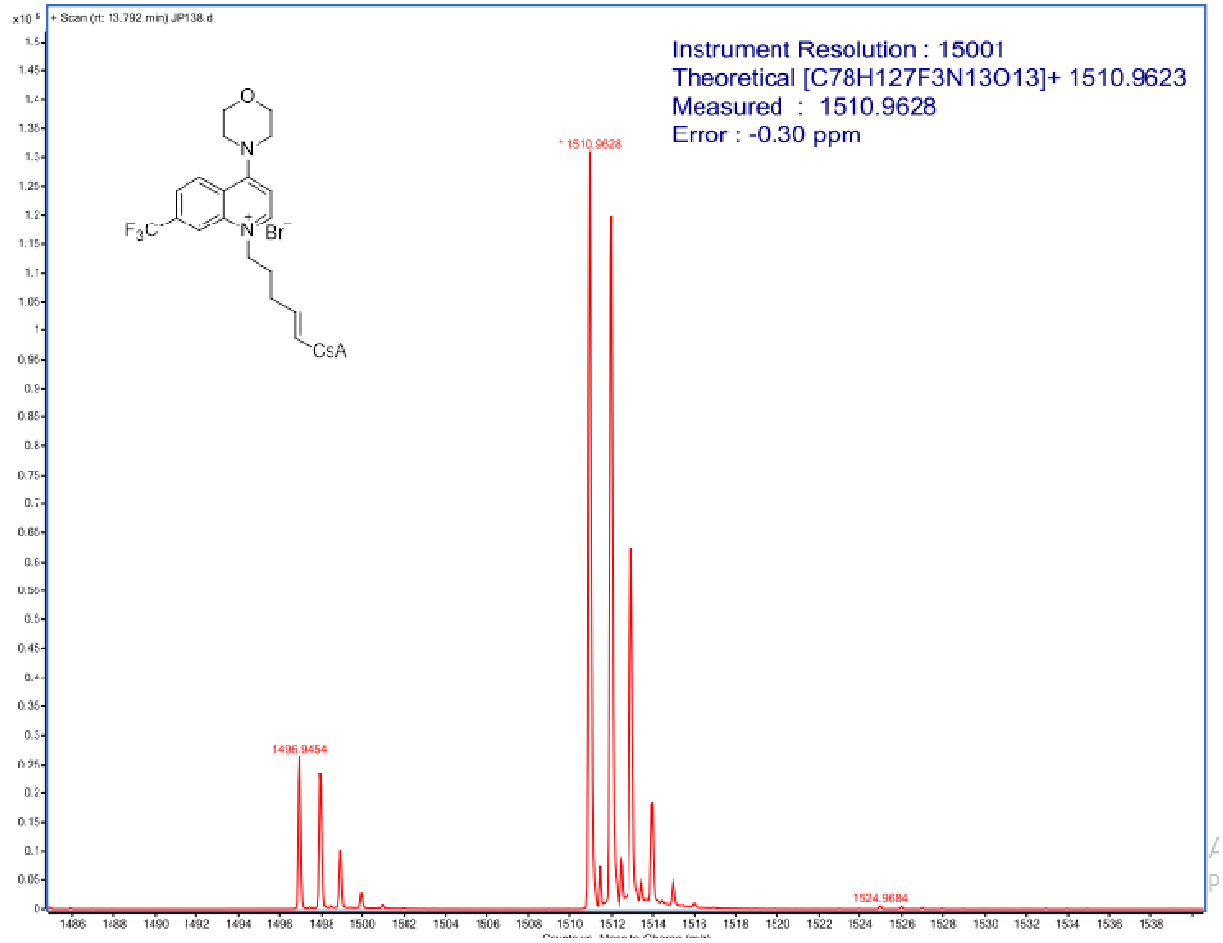
x10 5
+ Scan (rt: 13.792 min) JP138.d
Instrument Resolution : 15001
Theoretical [C78H127F3N13O13]+ 1510.9623
Measured : 1510.9628
Error : -0.30 ppm
1510.9628
1496.9454
1524.9684
O
N
F3C
N
Br
CsA

JP1-140

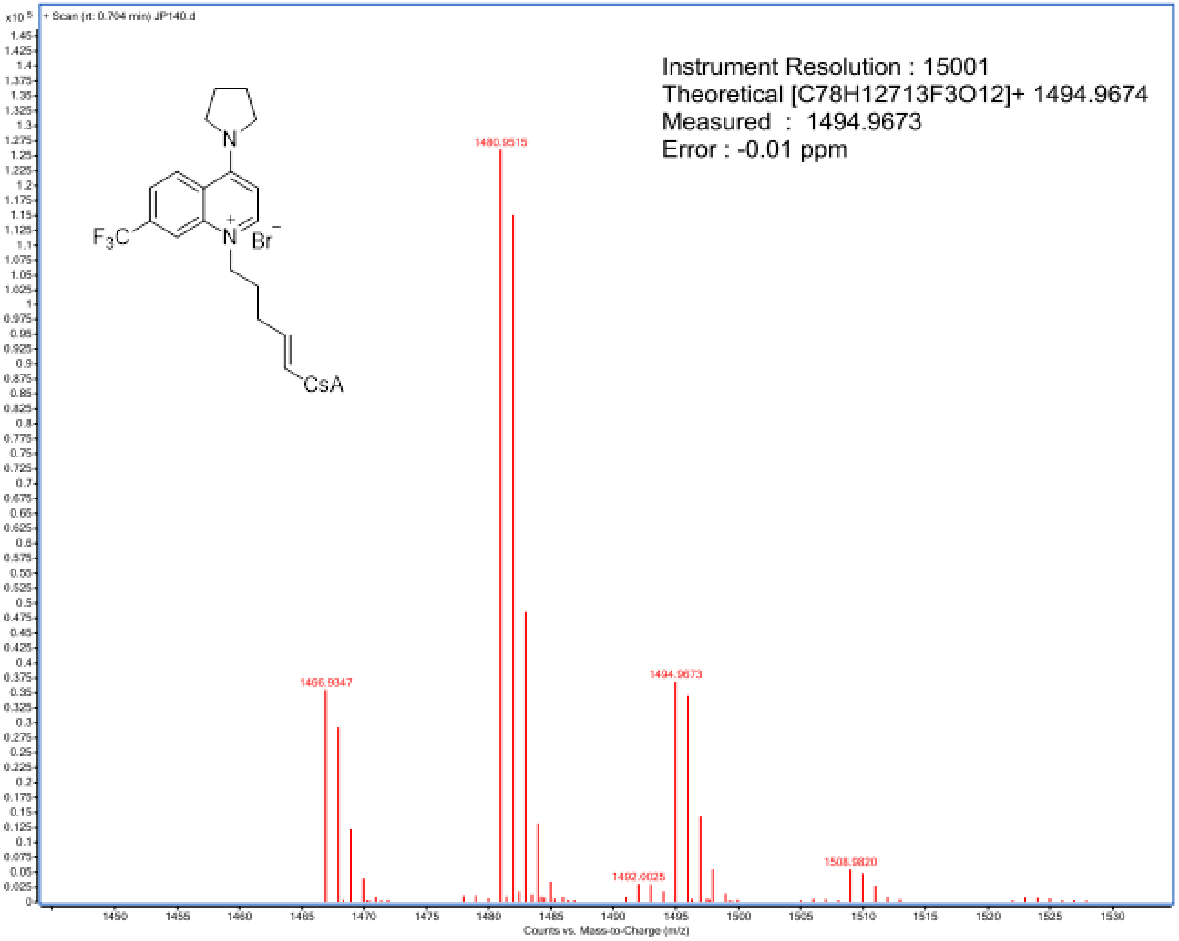

22/01/2018

Agilent LC system connected to Agilent
6510 Q TOF mass spectrometer

JP1-141

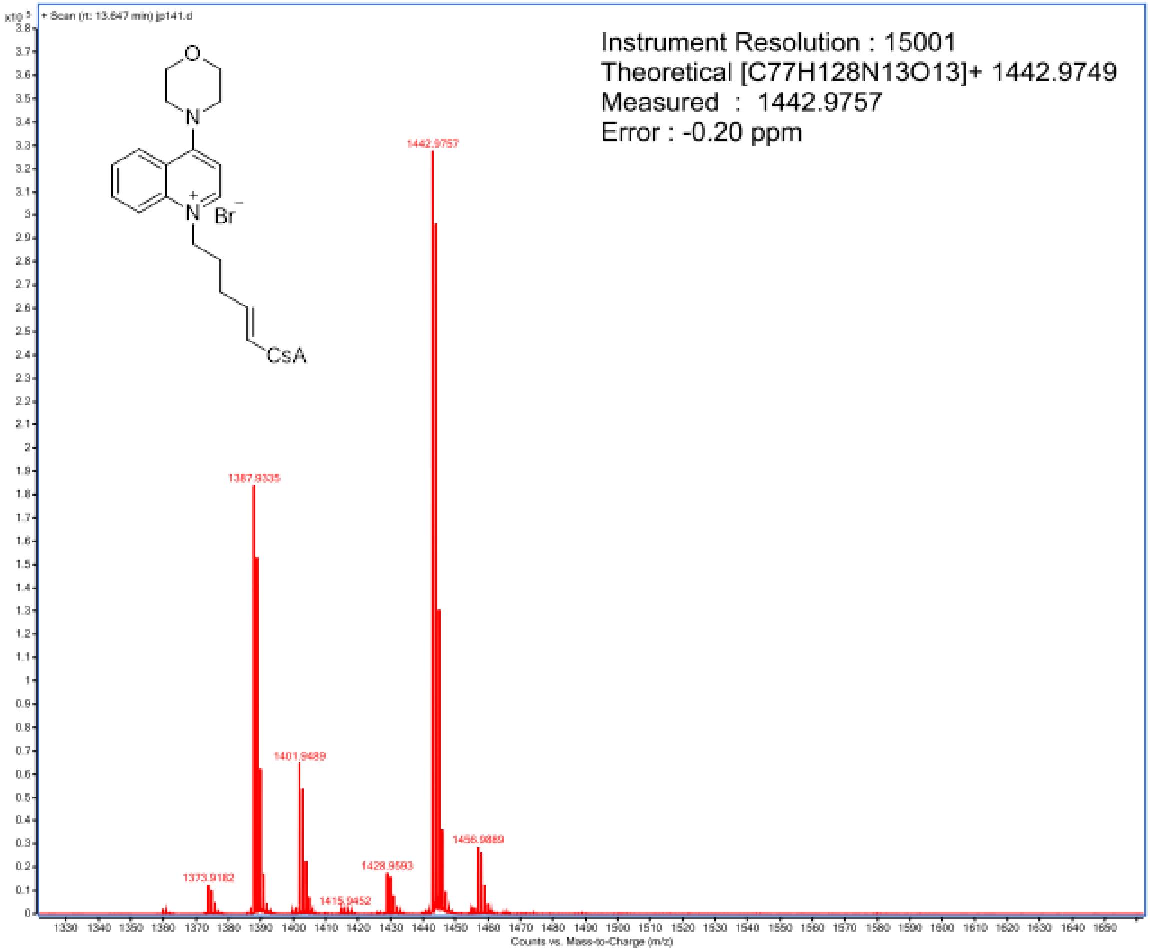

30/01/2018 Agilent LC system connected to Agilent 6510 Q TOF mass spectrometer

JP1-159

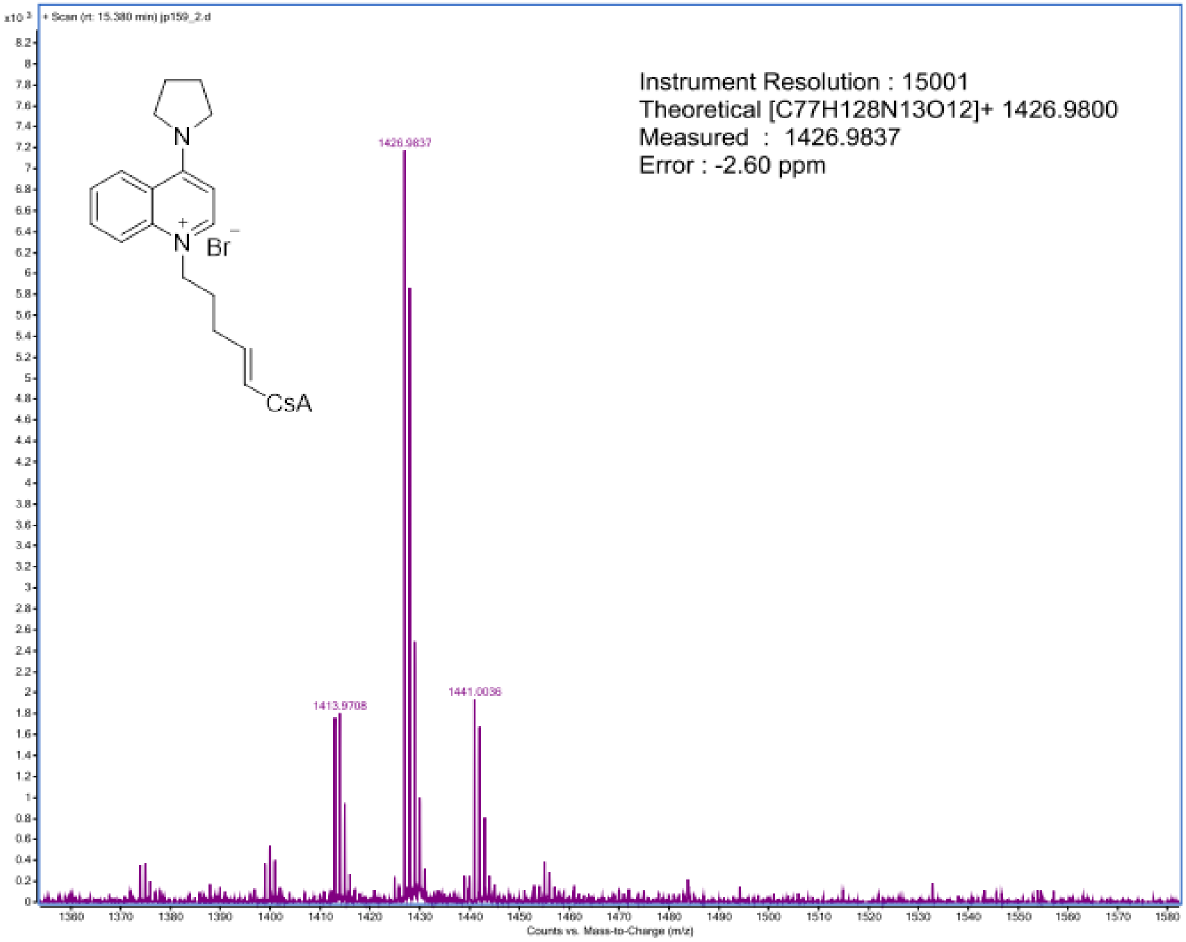
+ Scan (rt: 15.380 min) jp159_2.d
Instrument Resolution : 15001
Theoretical [C77H128N13O12]+ 1426.9800
Measured : 1426.9837
Error : -2.60 ppm
N
N
Br
CsA
1426.9837
1413.9708
1441.0036
Counts vs. Mass-to-Charge (m/z)

JP1-164

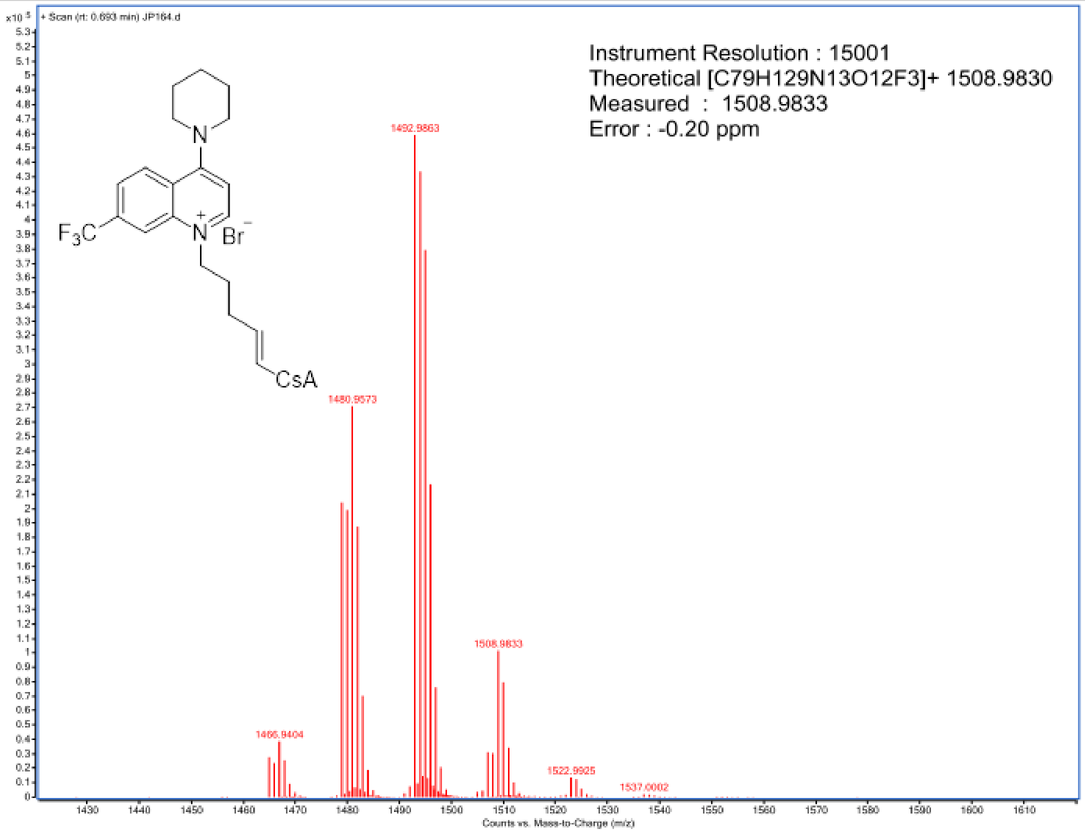

x10 5
+ Scan (rt: 0.693 min) JP164.d
Instrument Resolution : 15001
Theoretical [C79H129N13O12F3]+ 1508.9830
Measured : 1508.9833
Error : -0.20 ppm
N
F3C
N+
Br−
CsA
1492.9863
1480.9573
1508.9833
1466.9404
1522.9925
1537.0002
Counts vs. Mass-to-Charge (m/z)

JP1-166

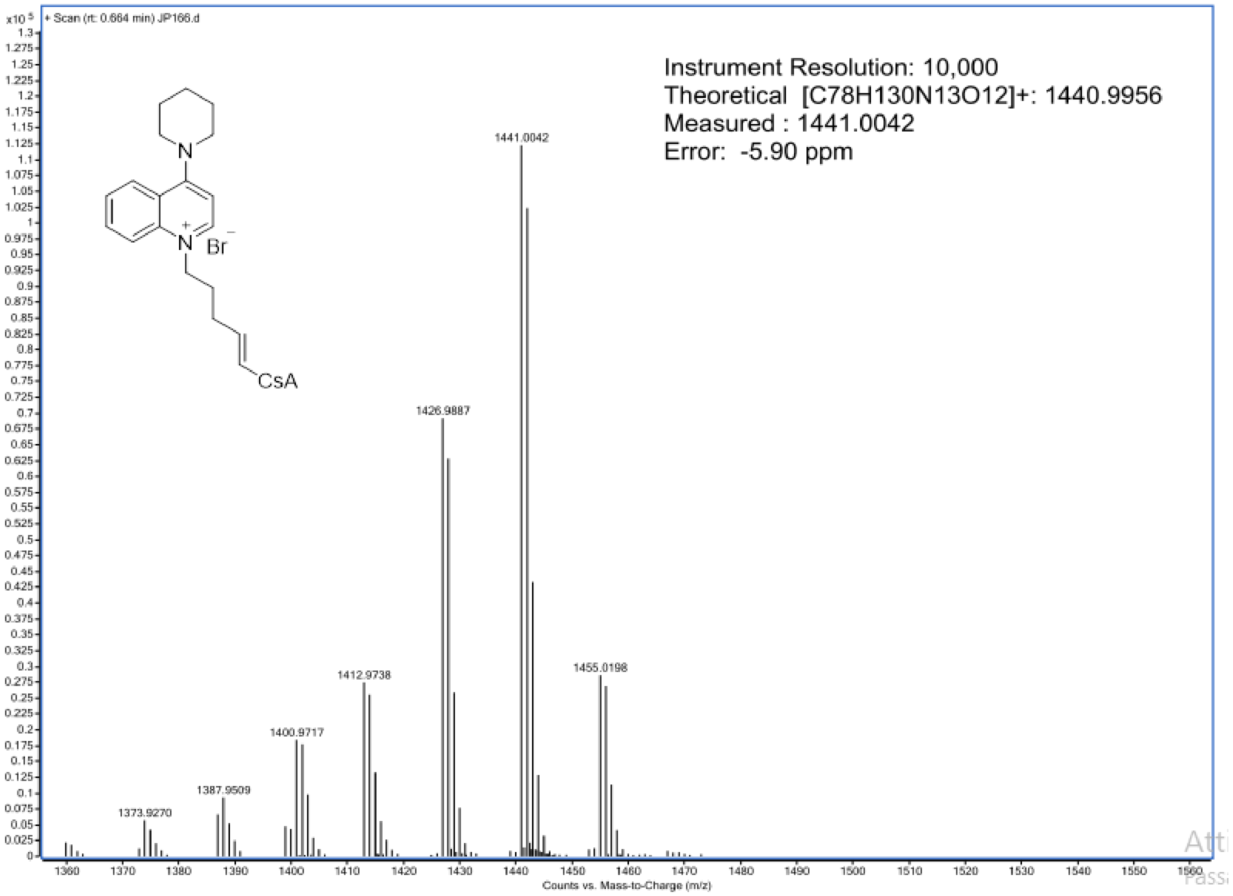

24/01/2018 Agilent LC system connected to Agilent 6510 Q TOF mass spectrometer 3

JP1-180

UCL Chemistry Mass Spectrometry Facility

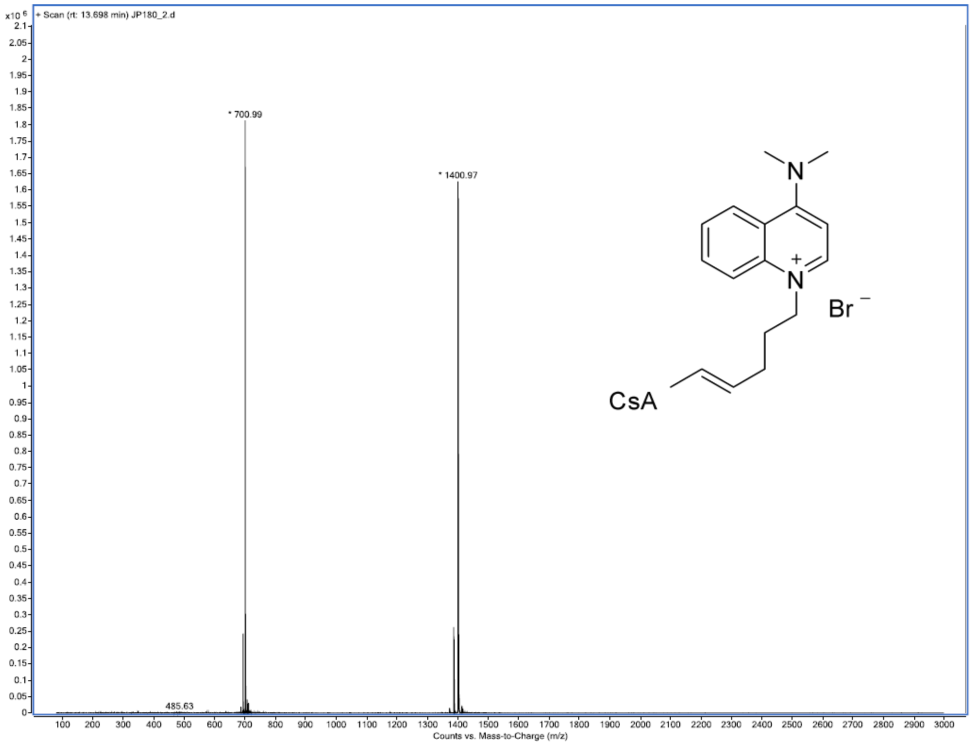

24/01/2018 Agilent LC system connected to Agilent 6510 Q TOF mass spectrometer

X-ray crystallographic data

X-ray crystallographic data for VP146

A red block-shaped-shaped crystal with dimensions 0.18×0.11×0.07 mm^3 was mounted on a MITIGEN holder in oil. X-ray diffraction data were collected using a Rigaku 007HF equipped with Varimax confocal mirrors and a UG2 goniometer and HyPix 6000 detector equipped with an Oxford Cryosystems low-temperature device, operating at T = 100(2) K.

Data were measured using profile data from ω-scans of ° per frame for s using Cu K_α radiation (Rotating-anode X-ray tube, 40.0 kV, 30.0 mA). The total number of runs and images was based on the strategy calculation from the program CrysAlisPro 1.171.42.89a (Rigaku Oxford Diffraction, 2023). The maximum resolution achieved was Θ = 76.620°.

Cell parameters were retrieved using the CrysAlisPro 1.171.42.89a (Rigaku Oxford Diffraction, 2023) software and refined using CrysAlisPro 1.171.42.89a (Rigaku OD, 2023) on 28754 reflections, 73 % of the observed reflections. Data reduction was performed using the CrysAlisPro 1.171.42.89a (Rigaku Oxford Diffraction, 2023) software which corrects for Lorentz polarisation. The final completeness is 99.80 % out to 76.620° in Θ.

An analytical absorption correction was performed using CrysAlisPro 1.171.42.89a (Rigaku Oxford Diffraction, 2023) Analytical numeric absorption correction using a multifaceted crystal model based on expressions derived by R.C. Clark & J.S. Reid (*53*). Empirical absorption correction using spherical harmonics, implemented in SCALE3 ABSPACK scaling algorithm. The absorption coefficient μ of this material is 36.799 mm^{-1} at this wavelength (λ = 1.54184Å) and the minimum and maximum transmissions are 0.022 and 0.269.

The structure was solved in the space group $P2_1/n$ (# 14) by using dual methods using the ShelXT 2018/2 (*54*) (*55*) structure solution program and refined by full matrix least squares minimisation on $\boldsymbol{F^2}$ using version 2018/3 of ShelXL 2018/3 (*55*). All non-hydrogen atoms were refined anisotropically. Hydrogen atom positions were calculated geometrically and refined using the riding model.

_exptl_absorpt_process_details: CrysAlisPro 1.171.42.89a (Rigaku Oxford Diffraction, 2023) Analytical numeric absorption correction using a multifaceted crystal model based on expressions derived by R.C. Clark & J.S. Reid (*13*). Empirical absorption correction using spherical harmonics, implemented in SCALE3 ABSPACK scaling algorithm.

There is a single molecule in the asymmetric unit, which is represented by the reported sum formula. In other words: Z is 4 and Z' is 1.

A summary of cell parameters, data collection, structure solution, and refinement for this crystal structure is given in the table. The corresponding crystallographic data were deposited with the Cambridge Crystallographic Data Centre with Deposition Number 2297286. The data can be obtained free of charge via https://www.ccdc.cam.ac.uk/.

Table: Crystal data for VP146.

**Compound**	**2023NCS0386_1a**
Formula	$C_{16}H_{18}F_3I_3N_2O$
$D_{calc.}$/ g cm^{-3}	2.277
μ/mm^{-1}	36.799
Formula Weight	692.02
Colour	red
Shape	block-shaped
Size/mm^3	0.18×0.11×0.07
T/K	100(2)
Crystal System	monoclinic
Space Group	$P2_1/n$
a/Å	8.54703(5)
b/Å	17.83235(10)
c/Å	13.24323(8)
α/°	90
β/°	90.1051(5)
γ/°	90
V/Å^3	2018.45(2)
Z	4
Z'	1
Wavelength/Å	1.54184
Radiation type	Cu K_α
Θ_{min}/°	4.158
Θ_{max}/°	76.620
Measured Refl's.	39221
Indep't Refl's	4079
Refl's I≥2 σ(I)	3961
R_{int}	0.0619
Parameters	227
Restraints	0
Largest Peak	0.872
Deepest Hole	-1.338
GooF	1.093
wR_2 (all data)	0.0699
wR_2	0.0691
R_1 (all data)	0.0287
R_1	0.0278

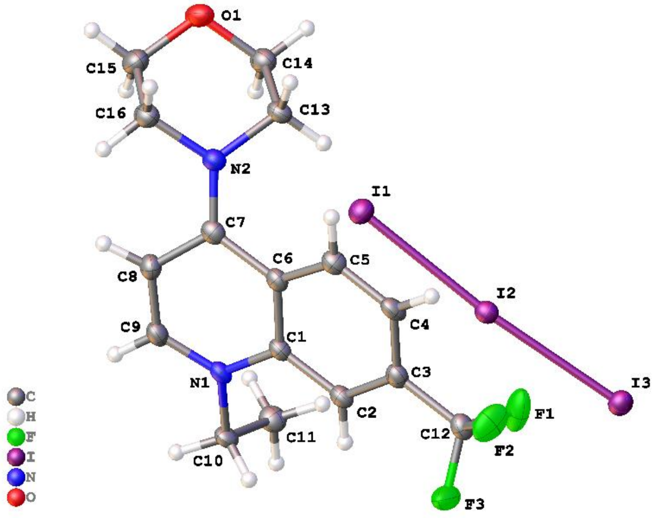

*ORTEP drawing of **VP146.***

X-ray crystallographic data for DS292A

A yellow block-shaped-shaped crystal with dimensions 0.210×0.100×0.070 mm^3 was mounted on a MITIGEN holder in oil. X-ray diffraction data were collected using a ROD, Synergy Custom system, HyPix-Arc 100 equipped with an Oxford Cryosystems low-temperature device, operating at T = 100.00(10) K. Data were measured using ω scans of 0.2 ˚ per frame for 0.0 s using Cu K_α radiation (Rotating-anode X-ray tube, 40.0 kV, 30.0 mA). The total number of runs and images was based on the strategy calculation from the program CrysAlisPro 1.171.42.94a (Rigaku Oxford Diffraction, 2023). The maximum resolution achieved was Θ = 72.110˚.

Cell parameters were retrieved using the CrysAlisPro 1.171.42.94a (Rigaku Oxford Diffraction, 2023) software and refined using CrysAlisPro 1.171.42.94a (Rigaku Oxford Diffraction, 2023) on 23205 reflections, 87 % of the observed reflections. Data reduction was performed using the CrysAlisPro 1.171.42.94a (Rigaku Oxford Diffraction, 2023) software which corrects for Lorentz polarisation. The final completeness is 99.90 % out to 72.110˚ in Θ.

A sphere absorption correction was performed using CrysAlisPro 1.171.42.94a (Rigaku Oxford Diffraction, 2023) Spherical absorption correction using equivalent radius and absorption coefficient. Empirical absorption correction using spherical harmonics, implemented in SCALE3 ABSPACK scaling algorithm. The absorption coefficient μ of this material is 23.074 mm^{-1} at this wavelength (λ = 1.54184Å) and the minimum and maximum transmissions are 0.013 and 0.086.

The structure was solved in the space group $P2_1/c$ (# 14) by using dual methods using the ShelXT 2018/2 (*14*) (*16*) structure solution program and refined by full matrix least squares minimisation on $\boldsymbol{F^2}$ using version 2018/3 of ShelXL 2018/3 (*15*). All non-hydrogen atoms were refined anisotropically. Hydrogen atom positions were calculated geometrically and refined using the riding model.

_exptl_absorpt_process_details: CrysAlisPro 1.171.42.94a (Rigaku Oxford Diffraction, 2023) Spherical absorption correction using equivalent radius and absorption coefficient. Empirical absorption correction using spherical harmonics, implemented in SCALE3 ABSPACK scaling algorithm.

There is a single formula unit in the asymmetric unit, which is represented by the reported sum formula.

In other words: Z is 4 and Z' is 1. The moiety formula is I, C_{11} H_{12} N.

A summary of cell parameters, data collection, structure solution, and refinement for this crystal structure are given in the table **S2**. The corresponding crystallographic data were deposited with the Cambridge Crystallographic Data Centre with Deposition Number 2297285. The data can be obtained free of charge via https://www.ccdc.cam.ac.uk/.

Table**: Crystal data for **DS292A.

**Compound**	**2023NCS0509_1b**
Formula	$C_{11}H_{12}IN$
$D_{calc.}$/ g cm^{-3}	1.766
μ/mm^{-1}	23.074
Formula Weight	285.12
Colour	yellow
Shape	block-shaped
Size/mm^3	0.210×0.100×0.070
T/K	100.00(10)
Crystal System	monoclinic
Space Group	$P2_1/c$
a/Å	7.46140(10)
b/Å	16.8307(3)
c/Å	8.60640(10)
α/°	90
β/°	97.079(2)
γ/°	90
V/Å^3	1072.56(3)
Z	4
Z'	1
Wavelength/Å	1.54184
Radiation type	Cu K_α
Θ_{min}/°	5.809
Θ_{max}/°	72.110
Measured Refl's.	26771
Indep't Refl's	2119
Refl's I≥2 σ(I)	2113
R_{int}	0.0349
Parameters	119
Restraints	0
Largest Peak	1.345
Deepest Hole	-0.528
GooF	1.126
wR_2 (all data)	0.0944
wR_2	0.0943
R_1 (all data)	0.0364
R_1	0.0364

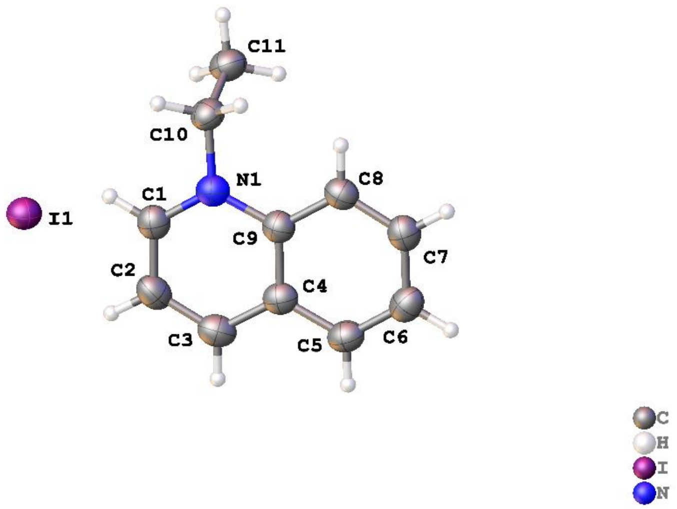

ORTEP drawing of DS292A.

Biology

Fluorescence Polarization assay.

This assay was performed as described previously (*12*). Briefly, titration of a single probe concentration against different enzyme concentrations was used to determine the dissociation constant (Kd). From this we also determined the enzyme concentration that would give a high enough polarization signal to measure binding affinities. The inhibitor constants (Ki), were calculated with the equation below (*56*).

$$Ki = \frac{[I]50}{\frac{[L]50}{Kd}+\frac{[P]0}{Kd}+1}$$

Where [I]50 is the concentration of the unlabelled compound at 50% inhibition, [L]50 is the concentration of the free probe-cyclophilin enzyme at 50% inhibition, [P]0 is the concentration of the free protein at 0% inhibition, and Kd is the dissociation constant of the probe-protein complex

Assays were conducted in 384-black low flange non-binding microtiter plates (Corning, Tewksbury, MA, USA). A total solution of 80 µL was used consisting of 3 components, fluorescent cyclosporine probe (FP-CsA) 45 nM, enzyme 40 nM, inhibitor (10-10000 nM). At least 3 replicates were used for each experiment. DMSO% in total solution should remain lower than 1%. FP-probe. Enzyme, inhibitor and FP-CsA are incubated in the OmegaFluostar at room temperature for 30 minutes, FP-CsA (40 µL) was added last. Measurements were taken after gain adjustment of control sample and 30 min incubation, with a xenon flash light with filter settings for 485 excitation and 520 emission.

Mitochondrial isolation

Adult C57BL/6 mice were sacrificed by cervical dislocation and the livers were extracted. The livers were first washed in PBS and then minced in isolation buffer (250 mM D-mannitol, 0.5 mM EGTA, 5 mM HEPES (ph7.4)) supplemented with 1 mM PMSF. The tissue was homogenised using a glass homogeniser with a Teflon pestle and a drill. Mitochondria were obtained from the homogenate by differential centrifugation, first at 800g to obtain the post-nuclear supernatant and then at 10,300g to obtain the crude mitochondrial pellet. The entire isolation protocol was carried out on ice or at 4˚C. The mitochondrial pellet was resuspended in isolation buffer. Protein concentrations were determined using the BCA assay (Pierce™ BCA Protein Assay Kit).

Calcium retention capacity assay

Calcium retention capacity was measured as described previously (*19*). Briefly, Ca^{2+} uptake measurements in isolated mitochondria (0.5mg/ml) were performed in MSK buffer (75 mM D-mannitol, 25 mM sucrose, 5 mM KH_2PO_3, 20 mM Tris-HCl, 100 mM KCl, and 0.1% BSA fatty acids free, pH 7.4) supplemented with 10 mM succinate and 1 µM rotenone to energise the mitochondria. 100 µl of the mitochondrial suspension was plated in a clear 96 well plate in triplicate for each condition. Extramitochondrial Ca^{2+} levels were quantified by measuring fluorescence intensity of the Ca^{2+} -sensitive dye Fluo-5N at 1µM concentration (Molecular Probes, F-14203). The measurements were obtained

using a plate reader (Fluostar Optima, BMG Labtech) at 30°C using the following filters; ex/em: 480 nm/520 nm. Ca^{2+} additions were achieved using integrated syringe injectors, where subsequent 10µl additions of 100 µM $CaCl_2$ were added for a total of 12 injections. The area under the curve was used as a measure of extramitochondrial Ca^{2+}, which was expressed as a proportion of total Ca^{2+} added (Ca^{2+} free condition was used for background subtraction). This value could be used to calculate the proportion of buffered Ca^{2+}, and subsequent percentage inhibitions were calculated compared to untreated.

Assay for cellular CypA selectivity

This assay was carried out as previously described (*12*). VSV-G pseudotyped GFP-encoding HIV-1 vector was prepared by triple plasmid transfection of HEK 293T cells as follows. 293T cells were transfected with 1 µg p8.91 (HIV-1 gag-pol expression vector) (*57*) + 1 µg pMDG (VSV-G expression vector) (*58*) + 1.5 µg pCSGW (lentiviral expression vector encoding enhanced GFP protein) (*59*) in 10 cm^2 dishes using 10 µL Fugene 6 transfection reagent (Promega) according to the manufacturer's instructions. The transfection mix was incubated for 15 mins at room temperature before adding dropwise to HEK 293 (*59*) T cells. Media was replaced after 24 hrs, and supernatants containing lentiviral vector were collected at 48 hrs and 72 hrs, filtered (0.45 µm), combined and stored at -80˚C.

To generate CRFK cells stably expressing N-terminally HA-tagged TRIM-CypA from an EXN-based vector, murine leukemia virus (MLV) vector was prepared by transfection of 293T cells as above. 293T cells were transfected with CMVi MLV gag-pol expression vector, pMDG, and γ-retroviral expression vector encoding a fusion protein comprising human CypA downstream of owl monkey TRIM5 RBCC (EXN-TRIM-CypA) (*60*). To prepare the control vector, empty γ-retroviral expression vector (EXN) was transfected. CRFK cells (*61*), which are null for TRIM5α activity, were then transduced with MLV vector (MLV EXN or MLV EXN-TRIM-CypA), followed by selection of cells in 1 mg/mL G418 (Invitrogen).

To test for the ability of compounds to rescue HIV-1 infectivity in the presence of TRIM-CypA, CRFK or CRFK-TRIMCypA cells (5 x 10^4 cells/well, 24 well plate) were treated with serial dilutions of DMSO, TWH106, JW76 or JP1-138 (0.5-20 µM) and infected with a single dose of GFP-encoding HIV-1 vector that infected 20% of CRFK cells (MOI 0.2). After 48 hrs, cells were fixed in 4 % formaldehyde in PBS and the % GFP-positive (infected) cells were determined using a NovoSampler Pro (Agilent) flow cytometer.

To determine toxicity of tested compounds, 3-4-5-dimethylthiazol-2-yl-2,5-diphenyltetrazolium bromide (MTT) assay (Sigma) was performed. CRFK and CRFK-TRIMCypA cells (1.25 x 10^4 cells/well, 96 well plate) were treated with serial dilutions of compounds. After 48 hrs, 10 % v/v MTT (stock at 5 mg/mL in PBS) was added and cells were incubated for 1-2 hrs at 37˚C. 100 µL solubilisation solution (10 % SDS 0.01 M HCl) was added and after overnight incubation at 37˚C, absorbance was measured at 570 nm.

In vivo T cell proliferation

ABH mice were sensitized on the ear with 25 µL of 2.5% oxazolone in acetone:olive oil [4:1]. Animals were treated daily from day 0-2 with various doses of either vehicle, cyclosporin A (CSA) or JPI-138 intraperitoneally in 50% DMSO:phosphate buffered saline in 0.1mL. Draining auricular lymph nodes from

3 mice were pooled on day 3 and 5 x 10*5 lymph node cells were cultured overnight in RMPI-1640 medium**.** Cell proliferation was assessed using a colorimetric XTT Tetrazolium assay as per manufacturers instructions (Roche).The results are the mean and standard error of triplicate samples.

Study approval. All animal procedures were approved by the local ethical review processes and Government Inspectors in accordance with UK Animals (Experimental Procedures) Act 1986, which incorporates directive 2010/63/EU. Experimental details, including: randomisation; powering and blinding, to conform with the ARRIVE (Animals in Research: Reporting In Vivo Experiments) guidelines have been reported previously (*62*).

Pharmacokinetic analysis.
ABH mice (n=4) were injected intraperitoneally with 0.1 ml of test compound. Animals were euthanised at the stated time with CO_2 overdose and blood was immediately collected from the heart following death and added to Microtainer (BD, Oxford, UK) tubes, centrifuged using an Eppendorf microfuge and plasma collected. Following the removal of blood the brain was rapidly (<30s) dissected from the skull and stored at -80°C prior to analysis. The analysis was conducted by a Contract Research Organisation (Cyprotex UK) using LCMSMS.

Computational study
Conformation generation and analysis

Conformation generation was performed using MOE 2022.2 using a "LowModeMD Search". This method uses a short molecular dynamics simulation using velocities with little kinetic energy on the high-frequency vibrational modes. 27 conformations were generated. Two distances were measured N1-C1, and C1-C2 for each conformer. dE is defined as the strain energy of the conformation relative to the lowest energy conformation with the same stereochemistry configuration. These three parameters were used to plot the 3D conformation plots in Figure 1B.

REFERENCES AND NOTES

1. T. T. Wager, X. Hou, P. R. Verhoest, A. Villalobos, Moving beyond rules: The development of a central nervous system multiparameter optimization (CNS MPO) approach to enable alignment of druglike properties. *ACS Chem. Nerosci.* **1**, 435–449 (2010).

2. A. Talevi, Central nervous system multiparameter optimization desirability, in *The ADME Encyclopedia: A Comprehensive Guide on Biopharmacy and Pharmacokinetics* (Springer International Publishing, 2021), pp. 1–8.

3. C. A. Lipinski, F. Lombardo, B. W. Dominy, P. J. Feeney, Experimental and computational approaches to estimate solubility and permeability in drug discovery and development settings. *Adv. Drug Deliv. Rev.* **46**, 3–26 (2001).

4. B. C. Doak, B. Over, F. Giordanetto, J. Kihlberg, Oral druggable space beyond the rule of 5: Insights from drugs and clinical candidates. *Chem. Biol.* **21**, 1115–1142 (2014).

5. M. Tyagi, V. Poongavanam, M. Lindhagen, A. Pettersen, P. Sjö, S. Schiesser, J. Kihlberg, Toward the design of molecular chameleons: Flexible shielding of an amide bond enhances macrocycle cell permeability. *Org. Lett.* **20**, 5737–5742 (2018).

6. R. D. Readnower, W. B. Hubbard, O. J. Kalimon, J. W. Geddes, P. G. Sullivan, Genetic approach to elucidate the role of cyclophilin d in traumatic brain injury pathology. *Cells* **10**, 199 (2021).

7. T. Briston, D. L. Selwood, G. Szabadkai, M. R. Duchen, Mitochondrial permeability transition: A molecular lesion with multiple drug targets. *Trends Pharmacol. Sci.* **40**, 50–70 (2019).

8. S. A. Barrientos, N. W. Martinez, S. Yoo, J. S. Jara, S. Zamorano, C. Hetz, J. L. Twiss, J. Alvarez, F. A. Court, Axonal degeneration is mediated by the mitochondrial permeability transition pore. *J. Neurosci.* **31**, 966–978 (2011).

9. J. L. Hazelton, M. Petrasheuskaya, G. Fiskum, T. Kristián, Cyclophilin D is expressed predominantly in mitochondria of gamma-aminobutyric acidergic interneurons. *J. Neurosci. Res.* **87**, 1250–1259 (2009).

10. M. Forte, B. G. Gold, G. Marracci, P. Chaudhary, E. Basso, D. Johnsen, X. Yu, J. Fowlkes, M. Rahder, K. Stem, P. Bernardi, D. Bourdette, Cyclophilin D inactivation protects axons in experimental autoimmune encephalomyelitis, an animal model of multiple sclerosis. *Proc. Natl. Acad. Sci. U.S.A.* **104**, 7558–7563 (2007).

11. F. D. Lublin, S. C. Reingold, J. A. Cohen, G. R. Cutter, P. S. Sørensen, A. J. Thompson, J. S. Wolinsky, L. J. Balcer, B. Banwell, F. Barkhof, B. Bebo Jr, P. A. Calabresi, M. Clanet, G. Comi, R. J. Fox, M. S. Freedman, A. D. Goodman, M. Inglese, L. Kappos, B. C. Kieseier, J. A. Lincoln, C. Lubetzki, A. E. Miller, X. Montalban, P. W. O'Connor, J. Petkau, C. Pozzilli, R. A. Rudick, M. P. Sormani, O. Stüve, E. Waubant, C. H. Polman, Defining the clinical course of multiple sclerosis: The 2013 revisions. *Neurology* **83**, 278–286 (2014).

12. J. Warne, G. Pryce, J. M. Hill, X. Shi, F. Lennerås, F. Puentes, M. Kip, L. Hilditch, P. Walker, M. I. Simone, A. W. E. Chan, G. J. Towers, A. R. Coker, M. R. Duchen, G. Szabadkai, D. Baker, D. L. Selwood, Selective inhibition of the mitochondrial permeability transition pore protects against neurodegeneration in experimental multiple sclerosis. *J. Biol. Chem.* **291**, 4356–4373 (2016).

13. T. A. Trendeleva, E. I. Sukhanova, A. G. Rogov, R. A. Zvyagilskaya, I. I. Seveina, T. M. Ilyasova, D. A. Cherepanov, V. P. Skulachev, Role of charge screening and delocalization for lipophilic cation permeability of model and mitochondrial membranes. *Mitochondrion* **13**, 500–506 (2013).

14. S. O. Kelley, K. M. Stewart, R. Mourtada, Development of novel peptides for mitochondrial drug delivery: Amino acids featuring delocalized lipophilic cations. *Pharm. Res.* **28**, 2808–2819 (2011).

15. P. G. Finichiu, A. M. James, L. Larsen, R. A. J. Smith, M. P. Murphy, Mitochondrial accumulation of a lipophilic cation conjugated to an ionisable group depends on membrane

potential, pH gradient and pK(a): Implications for the design of mitochondrial probes and therapies. *J. Bioenerg. Biomembr.* **45**, 165–173 (2013).

16. S. J. Coles, D. R. Allan, C. M. Beavers, S. J. Teat, S. J. W. Holgate, C. A. Tovee, Leading edge chemical crystallography service provision and its impact on crystallographic data science in the twenty-first century, in *21st Century Challenges in Chemical Crystallography I: History and Technical Developments,* D. M. P. Mingos, P. R. Raithby, Eds. (Springer International Publishing, 2020), pp. 69–140.

17. O. V. Dolomanov, L. J. Bourhis, R. J. Gildea, J. A. K. Howard, H. Puschmann, OLEX2: A complete structure solution, refinement and analysis program. *J. Appl. Cryst.* **42**, 339–341 (2009).

18. P. Atkins, *Shriver and Atkins' Inorganic Chemistry* (OUP Oxford, 2010).

19. K. N. Robertson, T. S. Cameron, O. Knop, Polyhalide anions in crystals. Part 2. I_3^-asymmetry and N—H … I bonding: Triiodides of the $Me_2NH_2^+$, Ph_2I^+, tropanium,N,N,N′,N′-Me_4–1,2-ethanediammonium,N,N,N′,N′-Me_4–1,3-propanediammonium,N-Me-piperazinium(2+), andN,N′-Me2-piperazinium(2+) cations, and Me_2NH_2I. *Can. J. Chem.* **74**, 1572–1591 (1996).

20. A. Bouzide, G. Sauvé, Highly selective silver(I) oxide mediated monoprotection of symmetrical diols. *Tetrahedron Lett.* **38**, 5945–5948 (1997).

21. S. H. Hong, D. P. Sanders, C. W. Lee, R. H. Grubbs, Prevention of undesirable isomerization during olefin metathesis. *J. Am. Chem. Soc.* **127**, 17160–17161 (2005).

22. J. Kong, C.-Y. Chen, J. Balsells-Padros, Y. Cao, R. F. Dunn, S. J. Dolman, J. Janey, H. Li, M. J. Zacuto, Synthesis of the HCV protease inhibitor Vaniprevir (MK-7009) using ring-closing metathesis strategy. *J. Org. Chem.* **77**, 3820–3828 (2012).

23. J. A. Bing, J. N. Johnston, Enantioselective synthesis of *cis*- and *trans*-cycloheptyl β-fluoro amines by sequential aza-Henry addition/ring-closing metathesis. *Org. Lett.* **25**, 950–955 (2023).

24. T. L. Davis, J. R. Walker, V. Campagna-Slater, P. J. Finerty, R. Paramanathan, G. Bernstein, F. MacKenzie, W. Tempel, H. Ouyang, W. H. Lee, E. Z. Eisenmesser, S. Dhe-Paganon, Structural and biochemical characterization of the human cyclophilin family of peptidyl-prolyl isomerases. *PLOS Biol.* **8**, e1000439 (2010).

25. G. Bhosale, M. R. Duchen, Investigating the mitochondrial permeability transition pore in disease phenotypes and drug screening. *Curr. Protoc. Pharmacol.* **85**, e59 (2019).

26. P. Czodrowski, hERG me out. *J. Chem. Inf. Model.* **53**, 2240–2251 (2013).

27. C. Gathmann, L. S. Newton, S. Ridewood, R. J. Smith, T. W. Hornsby, A.-K. Reuschl, A. Wijaya, K. L. Morling, Y. Y. Tan, L. G. Thorne, A. Ciulli, C. Jolly, G. J. Towers, D. L. Selwood, Synthetic PROTACs based on a depsipeptide macrocycle selectively degrade cyclophilin A and inhibit HIV-1. *Res. Sq.* 10.21203/rs.3.rs-2639894/v1 (2023).

28. A. H. Schinkel, E. Wagenaar, L. van Deemter, C. A. Mol, P. Borst, Absence of the mdr1a P-Glycoprotein in mice affects tissue distribution and pharmacokinetics of dexamethasone, digoxin, and cyclosporin A. *J. Clin. Invest.* **96**, 1698–1705 (1995).

29. E. Palma, T. Tiepolo, A. Angelin, P. Sabatelli, N. M. Maraldi, E. Basso, M. A. Forte, P. Bernardi, P. Bonaldo, Genetic ablation of cyclophilin D rescues mitochondrial defects and prevents muscle apoptosis in collagen VI myopathic mice. *Hum. Mol. Genet.* **18**, 2024–2031 (2009).

30. L. J. Martin, S. Semenkow, A. Hanaford, M. Wong, Mitochondrial permeability transition pore regulates Parkinson’s disease development in mutant α-synuclein transgenic mice. *Neurobiol. Aging* **35**, 1132–1152 (2014).

31. L. J. Martin, B. Gertz, Y. Pan, A. C. Price, J. D. Molkentin, Q. Chang, The mitochondrial permeability transition pore in motor neurons: Involvement in the pathobiology of ALS mice. *Exp. Neurol.* **218**, 333–346 (2009).

32. F. Du, Q. Yu, R. H. Swerdlow, C. L. Waites, Glucocorticoid-driven mitochondrial damage stimulates Tau pathology. *Brain* **146**, 4378–4394 (2023).

33. J. Kuo, S. S. Serrano, A. Grönberg, R. Massoumi, M. J. Hansson, P. Gallay, Cyclophilin inhibitor NV556 reduces fibrosis and hepatocellular carcinoma development in mice with non-alcoholic steatohepatitis. *Front. Pharmacol.* **10**, 1129 (2019).

34. C. L. Ahlbach, K. W. Lexa, A. T. Bockus, V. Chen, P. Crews, M. P. Jacobson, R. S. Lokey, Beyond cyclosporine A: Conformation-dependent passive membrane permeabilities of cyclic peptide natural products. *Future Med. Chem.* **7**, 2121–2130 (2015).

35. R. D. Betterton, T. P. Davis, P. T. Ronaldson, Organic cation transporter (OCT/OCTN) expression at brain barrier sites: Focus on CNS drug delivery. *Handb. Exp. Pharmacol.* **266**, 301–328 (2021).

36. M. Lemaire, A. Bruelisauer, P. Guntz, H. Sato, Dose-dependent brain penetration of SDZ PSC 833, a novel multidrug resistance-reversing cyclosporin, in rats. *Cancer Chemother. Pharmacol.* **38**, 481–486 (1996).

37. M. Marino, C. Gardana, A. Scialpi, G. Giorgini, P. Simonetti, C. Del Bo' An in vitro approach to study the absorption of a new oral formulation of berberine. *PharmaNutrition* **18**, 100279 (2021).

38. X.-S. Tan, J.-Y. Ma, R. Feng, C. Ma, W.-J. Chen, Y.-P. Sun, J. Fu, M. Huang, C.-Y. He, J.-W. Shou, W.-Y. He, Y. Wang, J.-D. Jiang, Tissue distribution of berberine and its metabolites after oral administration in rats. *PLOS ONE* **8**, e77969 (2013).

39. H.-M. Cui, Q.-Y. Zhang, J.-L. Wang, J.-L. Chen, Y.-L. Zhang, X.-L. Tong, Poor permeability and absorption affect the activity of four alkaloids from Coptis. *Mol. Med. Rep.* **12**, 7160–7168 (2015).

40. T. J. Tucker, M. W. Embrey, C. Alleyne, R. P. Amin, A. Bass, B. Bhatt, E. Bianchi, D. Branca, T. Bueters, N. Buist, S. N. Ha, M. Hafey, H. He, J. Higgins, D. G. Johns, A. D. Kerekes, K. A. Koeplinger, J. T. Kuethe, N. Li, B. Murphy, P. Orth, S. Salowe, A. Shahripour, R. Tracy, W. Wang, C. Wu, Y. Xiong, H. J. Zokian, H. B. Wood, A. Walji, A series of novel, highly

potent, and orally bioavailable next-generation tricyclic peptide PCSK9 inhibitors. *J. Med. Chem.* **64**, 16770–16800 (2021).

41. C. A. Olsen, M. Witt, J. W. Jaroszewski, H. Franzyk, Diols as building blocks in solid-phase synthesis of polyamine toxins by Fukuyama-Mitsunobu alkylation. *Synlett* **2004** 473–476 (2004).

42. S. T. A. Shah, K. M. Khan, A. A. Heinrich, M. I. Choudhary, W. Voelter, An efficient approach towards syntheses of ethers and esters using CsF-Celite as a solid base. *Tetrahedron Lett.* **43**, 8603–8606 (2002).

43. S. M. Guo, B. Chan, Y. J. Xie, C. G. Xia, H. M. Huang, Copper-catalyzed oxidative amination of Benzoxazoles via C-H and C-N bond activation: A new strategy for using tertiary amines as nitrogen group sources. *Org. Lett.* **13**, 522–525 (2011).

44. G. G. Fan, B. W. Jiang, W. Sang, H. Cheng, R. Zhang, B. Y. Yu, Y. Yuan, C. Chen, F. Verpoort, Metal-free synthesis of heteroaryl amines or their hydrochlorides via an external-base-free and solvent-free C-N coupling protocol. *J. Org. Chem.* **86**, 14627–14639 (2021).

45. L. Zhang, C. Cheng, J. Li, L. L. Wang, A. A. Chumanevich, D. C. Porter, A. Mindich, S. Gorbunova, I. B. Roninson, M. Q. Chen, C. McInnes, A selective and orally bioavailable quinoline-6-carbonitrile-based inhibitor of CDK8/19 mediator kinase with tumor-enriched pharmacokinetics. *J. Med. Chem.* **65**, 3420–3433 (2022).

46. M. I. Matheu, R. Echarri, S. Castillon, Stereoselective synthesis of nucleosides by metallocene-promoted activation of glycosyl fluorides. *Tetrahedron Lett.* **33**, 1093–1096 (1992).

47. J. P. Wolfe, S. Wagaw, J. F. Marcoux, S. L. Buchwald, Rational development of practical catalysts for aromatic carbon-nitrogen bond formation. *Acc. Chem. Res.* **31**, 805–818 (1998).

48. J. Kwak, M. Kim, S. Chang, Rh(NHC)-catalyzed direct and selective arylation of quinolines at the 8-position. *J. Am. Chem. Soc.* **133**, 3780–3783 (2011).

49. J. M. Anderson, N. D. Measom, J. A. Murphy, D. L. Poole, Bridge heteroarylation of bicyclo[1.1.1]pentane derivatives. *Org. Lett.* **25**, 2053–2057 (2023).

50. M. Balkenhohl, B. Heinz, T. Abegg, P. Knochel, Amination of phosphorodiamidate-substituted pyridines and related N-heterocycles with magnesium amides. *Org. Lett.* **20**, 8057–8060 (2018).

51. R. J. Steffan, M. A. Ashwell, J. C. Pelletier, W. R. Solvibile, E. M. Matelan, Substituted 2-(S)-hydroxy-3-(piperidin-4-yl-methylamino)-propyl ethers and substituted 2-aryl-2-(R)-hydroxy-1-(piperidin-4-yl-methyl)-ethylamine β-3 adrenergic receptor agonists. US Patent 6,506,901 (2001).

52. D. Selwood, D. Baker, G. Szabadkai, M. R. Duchen, J. M. Hill, J. N. D. Warne, Quinolium conjugates of cyclosporin. US Patent WO2016027089A1 (2015).

53. R. C. Clark. J. S. Reid, The analytical calculation of absorption in multifaceted crystals. *Acta Crystallogr. A Found. Crystallogr.* **51**, 887–897 (1995).

54. I. Usón, G. M. Sheldrick, An introduction to experimental phasing of macromolecules illustrated by SHELX; new autotracing features. *Acta Crystallogr. D Struct. Biol.* **74**, 106–116 (2018).

55. G. M. Sheldrick, SHELXT–integrated space-group and crystal-structure determination. *Acta Crystallogr. A Found. Adv.* **71**, 3–8 (2015).

56. Z. Nikolovska-Coleska, R. Wang, X. Fang, H. Pan, Y. Tomita, P. Li, P. P. Roller, K. Krajewski, N. G. Saito, J. A. Stuckey, S. Wang, Development and optimization of a binding assay for the XIAP BIR3 domain using fluorescence polarization. *Anal. Biochem.* **332**, 261–273 (2004).

57. R. Zufferey, D. Nagy, R. J. Mandel, L. Naldini, D. Trono, Multiply attenuated lentiviral vector achieves efficient gene delivery in vivo. *Nat. Biotechnol.* **15**, 871–875 (1997).

58. L. Naldini, U. Blomer, P. Gallay, D. Ory, R. Mulligan, F. H. Gage, I. M. Verma, D. Trono, In vivo gene delivery and stable transduction of nondividing cells by a lentiviral vector. *Science* **272**, 263–267 (1996).

59. J. W. Bainbridge, C. Stephens, K. Parsley, C. Demaison, A. Halfyard, A. J. Thrasher, R. R. Ali, In vivo gene transfer to the mouse eye using an HIV-based lentiviral vector; efficient long-term transduction of corneal endothelium and retinal pigment epithelium. *Gene Ther.* **8**, 1665–1668 (2001).

60. L. M. J. Ylinen, A. J. Price, J. Rasaiyaah, S. Hué, N. J. Rose, F. Marzetta, L. C. James, G. J. Towers, Conformational adaptation of asian macaque TRIMCyp directs lineage specific antiviral activity. *PLOS Pathog.* **6**, e1001062 (2010).

61. W. A. McEwan, T. Schaller, L. M. Ylinen, M. J. Hosie, G. J. Towers, B. J. Willett, Truncation of TRIM5 in the *Feliformia* explains the absence of retroviral restriction in cells of the domestic cat. *J. Virol.* **83**, 8270–8275 (2009).

62. S. Al-Izki, G. Pryce, J. K. O’Neill, C. Butter, G. Giovannoni, S. Amor, D. Baker, Practical guide to the induction of relapsing progressive experimental autoimmune encephalomyelitis in the Biozzi ABH mouse. *Mult. Scler. Relat. Disord.* **1**, 29–38 (2012).
